# ChromGene: gene-based modeling of epigenomic data

**DOI:** 10.1186/s13059-023-03041-5

**Published:** 2023-09-07

**Authors:** Artur Jaroszewicz, Jason Ernst

**Affiliations:** 1 Bioinformatics Interdepartmental Program, University of California, Los Angeles, Los Angeles, CA 90095 USA; 2 Department of Biological Chemistry, University of California, Los Angeles, Los Angeles, CA 90095 USA; 3 Eli and Edythe Broad Center of Regenerative Medicine and Stem Cell Research, University of California, Los Angeles, Los Angeles, CA 90095 USA; 4 Computer Science Department, University of California, Los Angeles, Los Angeles, CA 90095 USA; 5 Computational Medicine Department, University of California, Los Angeles, Los Angeles, CA 90095 USA; 6 Jonsson Comprehensive Cancer Center, University of California, Los Angeles, Los Angeles, CA 90095 USA; 7 Molecular Biology Institute, University of California, Los Angeles, Los Angeles, CA 90095 USA

**Keywords:** Chromatin, Machine learning, Hidden Markov models, Histone modifications, Epigenomics

## Abstract

Various computational approaches have been developed to annotate epigenomes on a per-position basis by modeling combinatorial and spatial patterns within epigenomic data. However, such annotations are less suitable for gene-based analyses. We present ChromGene, a method based on a mixture of learned hidden Markov models, to annotate genes based on multiple epigenomic maps across the gene body and flanks. We provide ChromGene assignments for over 100 cell and tissue types. We characterize the mixture components in terms of gene expression, constraint, and other gene annotations. The ChromGene method and annotations will provide a useful resource for gene-based epigenomic analyses.

**Supplementary Information:**

The online version contains supplementary material available at 10.1186/s13059-023-03041-5.

## Background

Genome-wide maps of epigenomic marks, such as histone modifications from ChIP-seq experiments and chromatin accessibility from DNase-seq or ATAC-seq experiments, provide valuable information for annotating the genome in a cell type-specific manner [1–7]. Notably, approaches have been developed for annotating the genome into “chromatin states” based on the combinatorial and spatial patterns of epigenomic marks inferred de novo from the data. These different chromatin states can correspond to different classes of genomic elements, including enhancers, promoters, and repressive regions [8–10]. Annotations from these methods have been used for a diverse range of applications, including understanding gene regulation and genetic variants associated with disease [2, 11, 12].

Typically, chromatin states annotate the genome on a per-position basis. However, for some applications with epigenomic data, it is desirable to conduct gene-based analyses, as is common with transcriptomic data [13–15], but taking full advantage of epigenomic data to generate gene-based annotations is less straightforward than for per-position annotations. The challenge with gene-based annotations is that the combination of epigenomic marks will vary along a gene in a position-dependent manner. Furthermore, protein-coding genes differ vastly in length, ranging from a few hundred base pairs to over 2 Mb (median 30 kb) [16]. One strategy for gene-based annotations is to focus on the chromatin state at the transcription start site (TSS) [8, 17]. While such an approach is largely independent of varying gene lengths, it ignores potentially important information throughout the gene body. A simple alternative strategy would be based on averaging each mark’s signal across the entire gene [18]. However, such an approach loses information about which marks co-occur along the gene, and it could be heavily confounded by gene length.

Another strategy has been to partition genes into regions and cluster the genes based on mark signal in those regions. For example, one study partitioned genes into five regions: the 500-bp region upstream of the TSS, the 500-bp region downstream of the TSS, and the remaining gene body into equal thirds [19]. The study then averaged the per-mark signal within each region, which were then clustered with the *k*-medoids algorithm. However, such an approach is dependent on the specific choices of the partition and loses information due to averaging within each partition.

Other work has solved related, but different, problems. EPIGENE used hidden Markov models (HMMs) to identify transcription units [20], but is not designed for clustering them. Hierarchical or multi-tiered HMMs have been proposed to capture broader domains, but are not designed to provide a single annotation per gene in a cell or tissue type [21–23]. (Henceforth, we will use the term “cell type” instead of “cell or tissue type” for ease of presentation.) EpiAlign was proposed to align chromatin states between two regions, such as genes, and identify corresponding regions [24], but is also not designed for de novo gene-based annotations.

There is thus a need for a principled, model-based method that can be used to generate de novo annotations of known genes based on the combinatorial and spatial information in data of multiple epigenomic marks. To address this, we introduce ChromGene. ChromGene uses a mixture of HMMs to model the combinatorial and spatial information of epigenomics maps throughout a gene body and flanking regions. Furthermore, ChromGene can learn a common model across multiple cell types and use it to generate per-gene annotations for each. ChromGene is distinguished from other methods in that its focus is assigning a single annotation for each gene, which can span an arbitrary length, as opposed to an annotation for each position in the genome.

Here, we apply ChromGene to ChIP-seq data of histone marks and DNase-seq data from over 100 cell types and produce per-gene annotations for each one. We describe these annotations with respect to their mark emissions and relate them to gene expression data and other external data, including genes with high probability of loss-of-function (LoF) intolerance (pLI) [25]. We show that ChromGene annotations have better agreement with gene expression and stronger Gene Ontology (GO) and cancer gene set enrichments than other methods. We expect the ChromGene annotations we have produced will be a resource for gene-based epigenomic analyses, and that the methodological approach will be useful for applications to other epigenomic data.

## Results

### Overview of ChromGene method

**Fig. 1:**
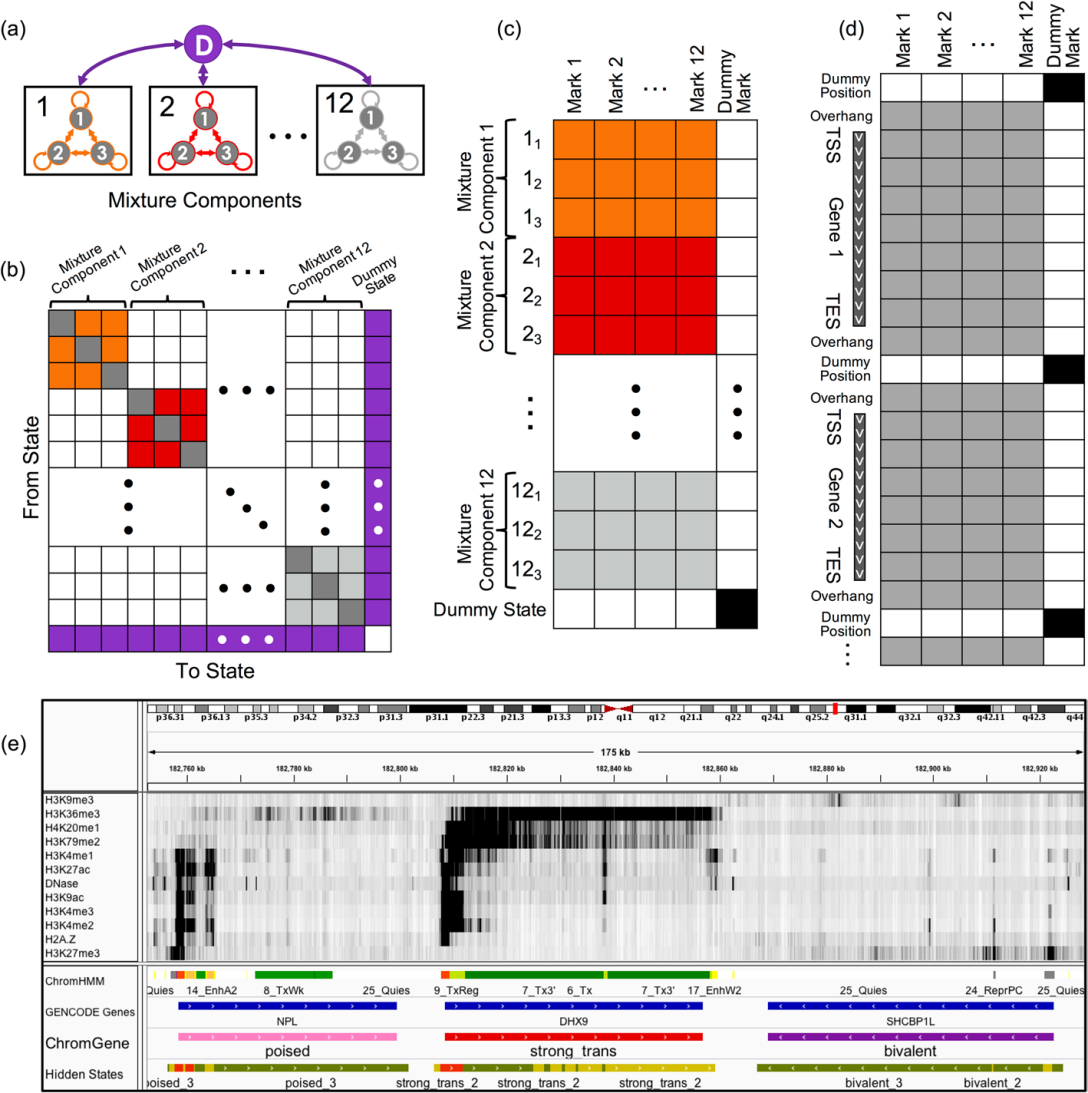
Overview of ChromGene. **a** ChromGene Model. Each gene is assumed to be generated by one of *M* = 12 mixture components, each of which is defined by *S* = 3 hidden states. Transitions within mixture components are allowed, but transitions between mixture components are only allowed by transitioning through the “dummy state” (purple), which only occurs between genes. **b** Transition matrix. The states within a component have learned probabilities of transition between them (colored and gray “self-transition” cells), but transitions between states in different components are disallowed (white). All states within components are allowed to transition to the dummy state (purple, right column), and the dummy state can transition to any state within any component (purple, bottom row). **c** Emission matrix. Each state has a separate emission probability for each input mark (colored and gray cells), corresponding to Bernoulli random variable parameters, and the probability of a set of observed marks is modeled using a product of those Bernoulli random variables. States within mixture components are enforced to never emit the “dummy mark” (white, right column), while the dummy state (bottom row) is enforced to never emit input marks (white) and always emit the dummy mark (black). **d** Data matrix. Input data across all genes and flanking regions is concatenated, with a single observation of “dummy position” between genes. Input data may be emitted within gene body or flanking regions (gray), but not at dummy positions (white cells). The dummy mark (right column) is only emitted at dummy positions (black cells). **e** IGV browser track [26] view using 12 input marks, “ChromHMM” annotations [27], “GENCODE Gene” track, mixture components (“ChromGene”), and components’ hidden states (“Hidden States”; red: state 1, yellow: state 2, green: state 3). Importantly, components’ hidden states are not comparable across different components and are not used in any analysis in this study

ChromGene models the set of epigenomic data across genes with a mixture of HMMs [9] (“Methods,” Fig. 1). The set of epigenomic data for each gene, along with a flanking region at each end (default 2 kb), is binarized at fixed-width bins (default 200 bp), indicating observations of each epigenomic mark, before being input to ChromGene. The data for a given gene in a cell type is modeled as being generated by one of *M* HMMs, each with *S* “hidden states” (or simply “states”) (black boxes, Fig. 1a). There are no constraints on allowed transitions between states of one HMM, but transitions between states of different HMMs are not allowed (Fig. 1b). There is also an initial state distribution, or the probability of seeing a state at the first position of the gene flanking region, over states of the HMMs. We note that otherwise, ChromGene does not directly model gene position information. The prior probability that a gene belongs to a specific mixture component, that is, an individual HMM, corresponds to the sum of initial probabilities of the states of that component. The emission distribution is modeled with a product of independent Bernoulli random variables following ChromHMM [9] (Fig. 1c). For given values of *M* and *S*, the parameters of the model are learned from the data using an expectation–maximization approach aimed to maximize the likelihood of the model parameters given the data (Fig. 1d). Once a model is trained, ChromGene computes the posterior probability of each of *M* mixture components generating each gene’s data in a given cell type. Finally, each gene is then assigned to the component for which it has the greatest posterior probability (Fig. 1e). We note that while ChromGene also assigns individual bins to hidden states based on the per-state posterior probability assignments (Fig. 1e), they are not our focus here, as there are effective model-based methods for position-level annotations.

To model data from multiple cell types, ChromGene can be applied analogously to the “concatenated” approach of ChromHMM [2, 28], which gives cell type-specific assignments based on a common model. To do this, ChromGene treats the same gene in different cell types as if it were different genes in the same cell type when learning the model. Using this common model, a gene is independently assigned to a mixture component for each cell type.

To develop ChromGene, we first noted that a mixture of HMMs can be equivalently expressed using a single, specially constructed HMM. In this single HMM, there is a “dummy state” that is associated with the emission of a “dummy mark,” which is only present before or after genes and their included flanking regions (Fig. 1a, “Methods”). All states within mixture components are initialized to allow transitions to and from this dummy state and between states of the same mixture component, but transitions between different mixture components are disallowed by enforcing those transition probabilities to be 0. Given the equivalence to a single HMM, we trained the ChromGene model by using ChromHMM after making enhancements to ChromHMM to better handle some technical numerical stability issues in this setting (“Methods”). We note that this strategy of reducing a mixture of HMMs to a single per-position HMM is general, and it could in principle be used to also provide other region-based annotations or to extend other software designed for per-position annotations, which may differ from ChromHMM in modeling assumptions.

### ChromGene generates distinct gene-level chromatin annotations

We applied ChromGene to imputed data for ten histone modifications (H3K9me3, H3K36me3, H4K20me1, H3K79me2, H3K4me1, H3K27ac, H3K9ac, H3K4me3, H3K4me2, and H3K27me3), histone variant H2A.Z, and DNase-seq data from 127 cell types, binarized at 200 bp resolution [1, 27] across 19,919 protein-coding genes with 2 kb flanking regions [16] using the “concatenated” approach. We focused our analysis on a model with *M* = *12* mixture components and *S* = *3* states per component to balance model expressivity with having meaningful distinctions between individual components and states within a component (“Methods,” Additional File 1: Fig. S1).

**Fig. 2:**
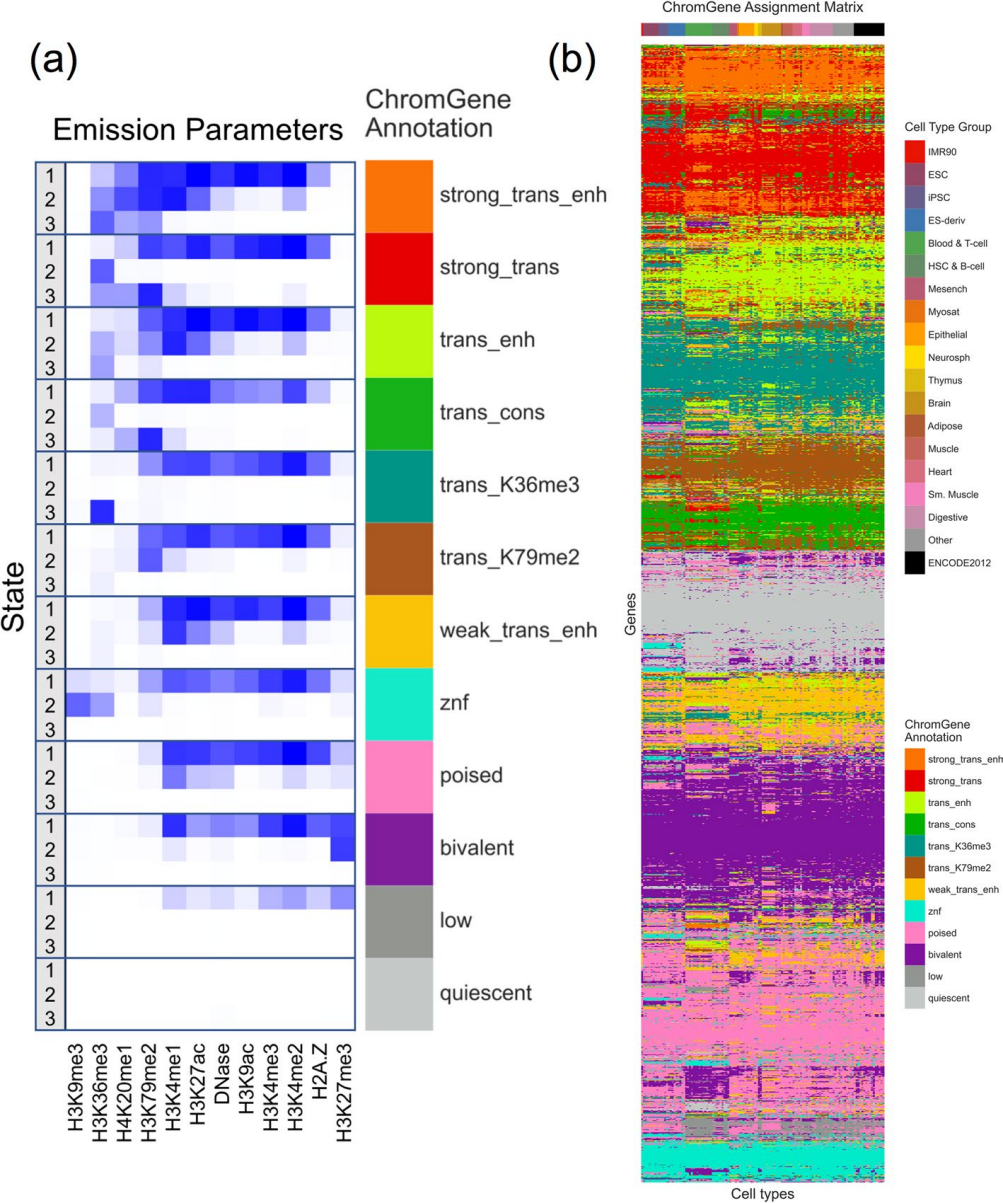
The emission parameters and assignment of genes to ChromGene annotations. **a** Heatmap of emission parameters with blue corresponding to a higher probability and white a lower. The ChromGene annotations are labeled on the right and the states within each annotation are labeled on the left. Annotations are ordered from top to bottom by decreasing expression, and states within each annotation are ordered by decreasing enrichment at the gene TSS. Marks are ordered from left to right as previously done [27]. Transition probabilities are pictured (Additional File 1: Fig. S2); per-state emission probabilities and enrichments, along with transition probabilities, are also reported (Additional File 2: Table S1). **b** Graphical representation of the ChromGene assignment matrix. Columns correspond to cell types, which are ordered as previously done [1], and their tissue group is indicated by the top colorbar (upper right legend). Rows correspond to 2000 subsampled genes (approximately 10% of all genes). Rows were ordered by hierarchical clustering (“Methods”). Each cell is colored by ChromGene annotation for the corresponding cell type and gene (lower right legend)

**Fig. 3:**
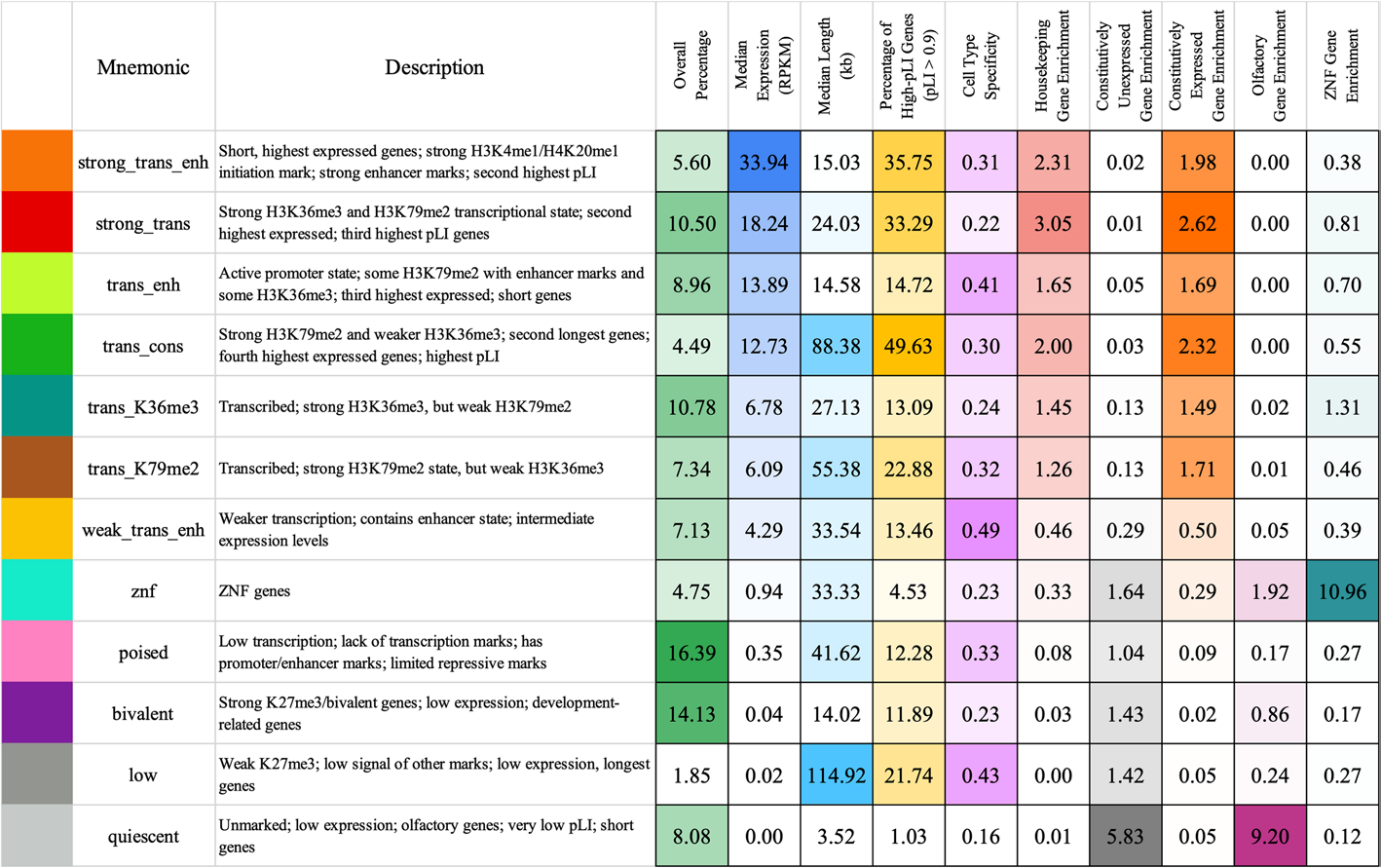
Brief description of and statistics on each ChromGene annotation. Rows correspond to ChromGene annotations. The columns are as follows: color used for ChromGene annotation; “Mnemonic”—abbreviated name used for annotation; “Description” of each ChromGene annotation based on mark emissions, expression, length, pLI, and other enrichments. Subsequent columns describe summary statistics of ChromGene annotations: “Overall Percentage”, “Median Expression (RPKM)”, and “Median Length (kb)” of genes assigned to annotation; “Percentage of High-pLI Genes (pLI ≥ 0.9)”—percentage of genes assigned to annotation with ≥ 0.9 pLI (probability of Loss of function Intolerance); “Cell Type Specificity”—metric of variability of annotation across cell types; “Housekeeping Gene Enrichment,” “Constitutively Unexpressed Gene Enrichment,” “Constitutive Expressed Gene Enrichment,” “Olfactory Gene Enrichment,” “ZNF Gene Enrichment”—fold enrichment of gene category within annotation compared to “Overall Percentage”

Based on the emission parameters of the model (Fig. 2a), along with the relationship of the components to external data not used in model learning (discussed below), we gave each component a candidate annotation (Fig. 3, Additional File 2: Table S1), which we will use to refer to these components henceforth. Eight of the annotations (“strong_trans_enh,” “strong_trans,” “trans_enh,” “trans_cons,” “trans_K36me3,” “trans_K79me2,” “weak_trans_enh,” and “znf”) (“trans”: “transcribed”, “enh”: “enhancer”, “cons”: “constrained”, “K36me3”: “H3K36me3”, “K79me2”: “H3K79me2”) had at least one state with H3K36me3 or H3K79me2, both transcription-associated histone modifications, present at > 31% of positions. All of these annotations had at least one state associated with high frequency of promoter-associated H3K4me3 or H3K4me2 (> 73%) and limited detection of the repressive mark H3K27me3 (< 15%). For four of these annotations (“strong_trans_enh”, “strong_trans”, “trans_enh”, and “trans_cons”), all three of its corresponding states had a high frequency (> 28%) of at least one mark. In contrast, the annotations “trans_K36me3”, “trans_K79me2”, “weak_trans_enh”, and “znf” all had one state with low frequency of all marks (< 7%). Annotation “znf” is notable in that H3K36me3 co-occurs with the repressive mark H3K9me3. This annotation shows 11-fold enrichment for zinc finger named (ZNF) genes on average. This is consistent with previous findings that ZNF genes enrich for per-position chromatin states associated with this combination of marks [8, 27], but in contrast to previous work, we also provide a direct model-based annotation of all genes associated with an epigenomic pattern highly enriched in ZNF genes. Three of the annotations (“strong_trans_enh”, “trans_enh”, and “weak_trans_enh”) had a state in which the H3K4me1 mark had the greatest frequency (> 78% in all cases) and limited H3K4me3 (< 5% of positions), consistent with previously described putative enhancers [8, 27, 29], suggesting these ChromGene annotations contain intragenic enhancers. Annotations “trans_K36me3” and “trans_K79me2” both had a state with a high frequency of active marks typically found at promoters or enhancers, a state with a low frequency of all marks, and a state dominated by H3K36me3 and H3K79me2, respectively.

The four remaining annotations (“poised”, “bivalent”, “low”, “quiescent”) lacked any state with the transcription-associated marks, H3K36me3 or H3K79me2, at a high frequency (< 12% for all states). Annotations “poised” and “bivalent” had states with high frequencies of other marks. Notably, the “bivalent” annotation had a state associated with the high presence of the repressive mark H3K27me3 in combination with H3K4me1/2/3, along with another state associated with H3K27me3 alone. Annotation “low” only had one state associated with moderate levels of epigenomic marks, none of which were associated with transcription. No state of the “quiescent” annotation had any detected modifications (all emissions < 1%).

The assignments for all 19,919 genes across 127 cell types are provided (Additional File 3) [30, 31]. To visualize the assignments, we sampled 2000 genes and clustered them based on assignment to ChromGene annotations across all cell types (“Methods,” Fig. 2b).

### ChromGene relationship with gene expression levels

**Fig. 4:**
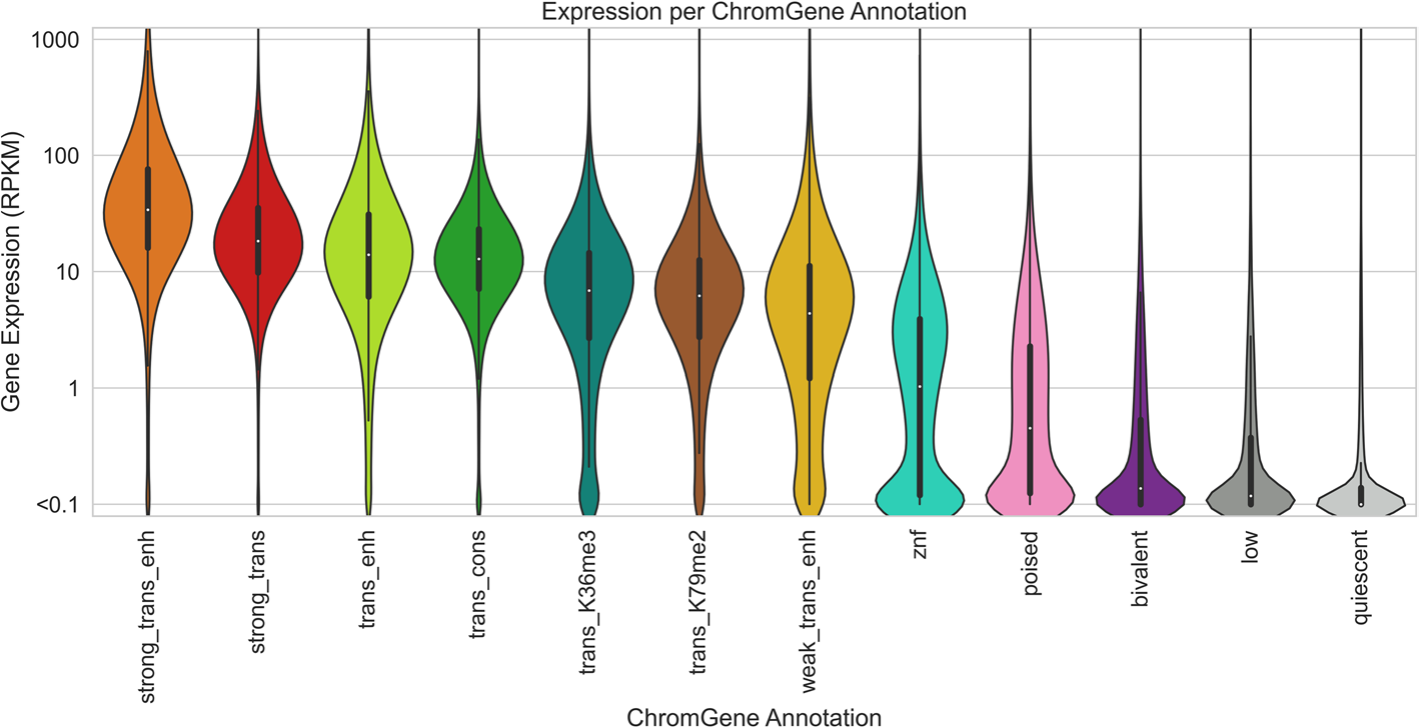
Gene expression distribution of ChromGene annotations. The gene expression distribution for each ChromGene annotation across 56 cell types. Gene expression values are in RPKM after adding a pseudocount of 0.1, and then log_10_ transforming for visualization

We first investigated how a gene's assignment to a ChromGene annotation relates to its expression level. We compared ChromGene annotations to matched gene expression data for 56 cell types [1] (“Methods”). We separated cell type-gene combinations by their ChromGene annotation and analyzed the distribution of gene expression values (RPKM) for each annotation. We observed that genes assigned to different annotations had varying levels of expression (Fig. 4). The two annotations with the highest expression (“strong_trans_enh” and “strong_trans”) both had states with a high frequency of H3K36me3 and H3K79me2. The four annotations with the lowest expression (“poised,” “bivalent,” “low,” and “quiescent”) each had a median RPKM of less than 1. Notably, the “quiescent” and “bivalent” annotations had a large overlap in distribution of gene expression levels, despite the lack of the former with any state with substantial frequency of epigenomic marks, and the association of the latter with epigenomic marks in multiple states. This highlights how ChromGene provides additional information about genes not captured by gene expression.

For each ChromGene annotation, we also determined how consistent average gene expression levels were across cell types. Specifically, for each annotation and cell type, we calculated the median log_10_(RPKM + 0.1) expression and analyzed the medians as a function of annotation (Additional File 1: Fig. S3). We then calculated the mean within-annotation variance across cell types (0.026) and the total variance of the computed medians across cell types and annotations (0.783). We found that the median expression variances within annotations were significantly smaller than across annotations (*p*-value < 10^−4^, permutation test), suggesting that ChromGene annotations are informative of expression regardless of cell type.

We also quantified how predictive ChromGene assignments are of gene expression and compared it to several baselines. To do this, we first separated genes into two groups: “expressed” genes with RPKM ≥ 1 and “unexpressed” genes with RPKM < 1. We held out genes on one chromosome at a time as a test set and used genes on all other chromosomes as a training set. We calculated the median expression for each ChromGene annotation for genes in the training set and used this as a predictor for the expression of genes in the held-out chromosome, and we found that the ChromGene assignment was a strong predictor of whether genes were expressed or unexpressed (across 56 cell types: mean AUROC = 0.893, standard deviation = 0.021). We also calculated the mean squared error (MSE) between the predicted and observed log_10_(RPKM + 0.1) expression values across all cell types, and we found that predicted expression values were close to the true expression (across 56 cell types: mean MSE = 0.418, standard deviation = 0.050; Pearson *r* = 0.763). We repeated the evaluations for the following three baseline models (“Methods”).

#### TSS model

The TSS model clusters genes based only on epigenomic mark information at the TSS. This model only considers one position per gene, and thus does not capture additional information throughout the gene.

#### Gene average model

The gene average model clusters genes based on the average epigenomic mark binarized values throughout the gene, which is equivalent to using ChromGene with only one state per HMM. This model has two key disadvantages: First, it does not take advantage of spatial information; instead, it treats heterogeneous spatial data as if it were homogeneous, eliminating potentially useful information. Second, it is more likely to be biased by gene length because epigenomic marks preferentially associate with specific genic regions that scale differently with gene length (see below).

#### Collapsed model

The collapsed model clusters genes using a single state per mixture component, as in the gene average model, but here the single state is found by first training a normal multi-state ChromGene model, then “collapsing” each multi-state HMM into a single-state HMM by taking the weighted average of the states in that component. This is meant to show that the differences between ChromGene and a single-state model are likely due to incorporating spatial information of epigenomic marks instead of different instantiations of the models.

We implemented each baseline method to have an identical number of clusters as our ChromGene model. ChromGene assignments were significantly more predictive of gene expression than the three baseline methods (Table S2, AUROC = 0.893 vs 0.818, 0.889, and 0.888; MSE = 0.418 vs 0.685, 0.429, and 0.438; Pearson *r* = 0.763 vs 0.601, 0.757, and 0.753; *p* < 10^−4^ for all comparisons, binomial test, for ChromGene vs TSS, gene average, and collapsed models, respectively, “Methods”).

### ChromGene reduces association of gene length with clusters

**Fig. 5:**
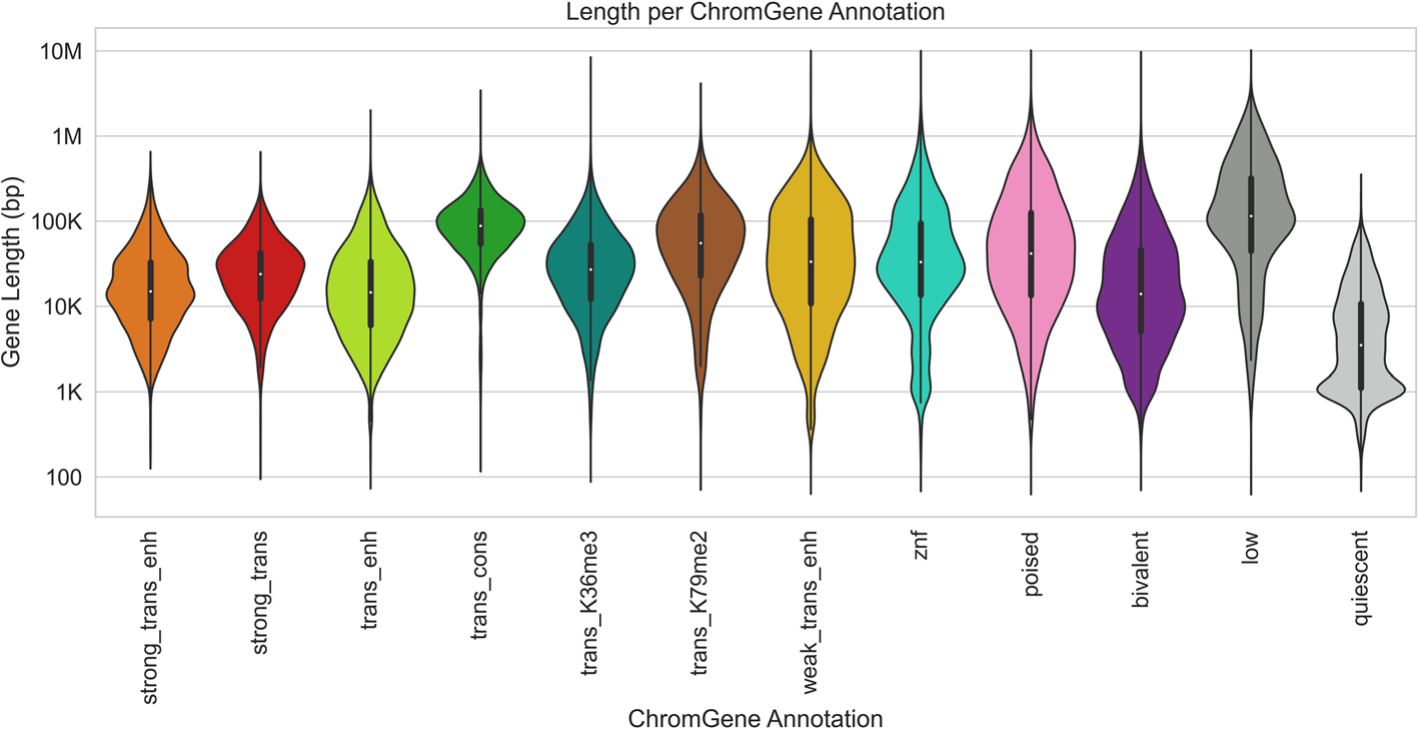
Lengths of genes by ChromGene annotation. Length distribution of different ChromGene annotations. Length axis is log-transformed for visualization

We next analyzed the relationship between the ChromGene annotations and the lengths of genes assigned to them (Fig. 5). We found that the length distribution of most annotations largely overlapped and were concentrated between 10 and 100 kb, but there were a few exceptions. Notably, “low” and “trans_cons” had median gene lengths around 100 kb, while “quiescent” was mostly composed of genes shorter than 10 kb. This “quiescent” annotation was strongly enriched (9.2 fold) for olfactory genes, which have a median length of 1034 bp and are expressed at low levels in most cell types (mean expression = 0.04 RPKM, median expression = 0).

Because gene length distributions varied across ChromGene annotations, we wanted to ensure that ChromGene provided substantial information beyond gene length. We thus evaluated the amount of information shared between ChromGene annotations and gene length, and for comparison, conducted the same evaluations for annotations from the three baseline models. Specifically, for each model and each cell type, we calculated the mutual information of the gene’s assignment and its length. We expected that the baseline TSS model would share the least information with gene length, as it only incorporates data from the TSS and not the whole gene, while among the models that incorporate data from the whole gene, ChromGene would share the least information. Indeed, we found that across cell types, the TSS model had the lowest mutual information (mean of 0.10), and ChromGene had significantly lower mutual information (mean of 0.31) than the gene average and collapsed models (mean of 0.46 and 0.48, respectively, *p* < 10^–30^, binomial test, “Methods,” Additional File 1: Fig. S4). This indicates that of the models that use information throughout the gene, ChromGene annotations are least likely to simply reflect gene length.

### Characterizing ChromGene assignments across cell types

**Fig. 6:**
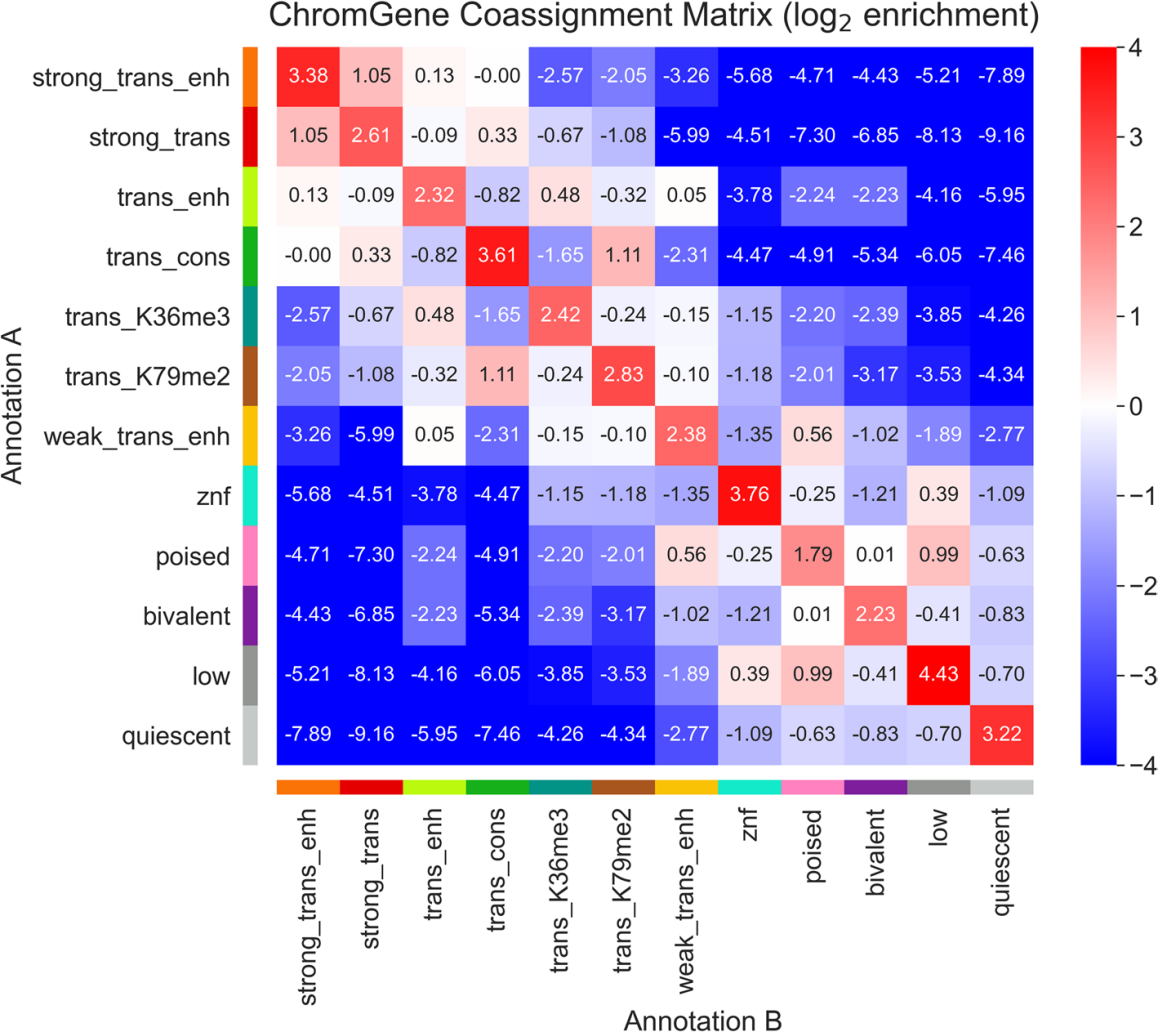
ChromGene co-assignment matrix enrichment. The log_2_ enrichment of combinations of ChromGene assignments over all pairs of non-replicate cell types. Enrichment corresponds to an increased likelihood of two cell types having the corresponding ChromGene assignments for a given gene, relative to randomly choosing based on ChromGene cluster sizes (“Methods”)

We hypothesized that for a given gene, some ChromGene annotations were more likely to be consistently assigned across cell types, while other annotations were more likely to co-occur with other annotations. To test this, we conducted enrichment analyses for the co-occurrence of ChromGene assignments across cell types (excluding those that could be considered essentially biological replicates) (Fig. 6, “Methods”). We found that certain pairs of ChromGene annotations are strongly depleted for a given gene across pairs of cell types. For example, genes assigned in one cell type to “trans_cons,” which is highly enriched with housekeeping genes, are strongly depleted for the “quiescent” annotation in other cell types (176 fold depletion compared to expected, corresponding to a −7.46 log_2_ ratio), consistent with the “quiescent” annotation’s enrichment for olfactory genes. On the other hand, we also found certain pairs of annotations enriched for the same gene across cell types. For example, genes assigned to “poised” in some cell type were enriched for both “weak_trans_enh” and “low” annotations in others, reflecting this annotation’s association with genes that can be activated or repressed in other cell types [32].

As assignments of genes to different annotations across cell types could be expected based on technical variability, we also estimated a confusion matrix for pairs of “replicate” cell types (Additional File 1: Fig. S5a, “Methods”). We found 81.1% concordance in assignments between replicate cell types, as compared to 10.2% expected by the number of genes assigned to each annotation. We next followed a similar process to calculate a contingency table for non-replicates—the probability of a given gene being assigned to one annotation for one cell type given it was assigned to another annotation in another cell type (Additional File 1: Fig. S5b). We found that between pairs of randomly chosen non-replicate cell types, ChromGene assignments were 57.1% concordant, which is substantially lower than between replicates (81.1%) and substantially higher than random assignment (10.2%).

We further defined the “cell type specificity” of each ChromGene annotation by dividing the diagonal of the contingency table (across non-replicate cell types) by the confusion matrix diagonal (the probability biological replicates would be assigned to the same annotation), then subtracting this value from 1 to obtain a “cell type specificity score” so that higher values correspond to more cell type-specific annotations. We found that the annotations that varied the least across cell types were “quiescent,” “strong_trans,” “znf,” “bivalent,” and “trans_K36me3” with cell type specificity scores from 0.16 to 0.24 (Fig. 3, Additional File 2: Table S1). The most cell type-specific annotations were “weak_trans_enh,” “low,” and “trans_enh” with scores of 0.49, 0.43, and 0.41 respectively. “Weak_trans_enh” having the highest cell type specificity score is consistent with this annotation being primarily associated with enhancers (Additional File 1: Fig. S6), which are known to be highly cell type-specific [2, 33]. The “low” annotation also had high cell type specificity despite having similarly low expression as the “quiescent” annotation, and it was often assigned to the annotation “poised” in other cell types (Fig. 6).

We next evaluated the extent to which changes in a gene’s ChromGene assignment across pairs of cell types were reflected (on average) in a change in expression of that gene (Additional File 1: Fig. S7a). We found that for most pairs of ChromGene annotations, changes in expression were largely consistent with their overall individual expression patterns (Fig. 4, Additional File 1: Fig. S7c,e). For example, if a gene was assigned in cell type *i* to a low-expression annotation, such as “quiescent,” and a high-expression annotation in cell type *j*, such as “strong_trans_enh,” then the expression of that gene in cell type *j* would typically be substantially higher than in *i* (“Methods”). Interestingly, we found a few pairs of ChromGene annotations for which expression did not vary as much as expected based on individual patterns (Additional File 1: Fig. S7c,e). Together, these results show that while changes in ChromGene assignment across cell types are typically reflected in expression changes for their associated annotations, some genes do not follow this pattern.

### ChromGene annotations are differentiated by gene set enrichments

We next analyzed the enrichment of ChromGene annotations with respect to various gene sets (Additional File 2: Table S1). These gene sets included ZNF-named genes, constitutively unexpressed genes, housekeeping genes, olfactory genes, “biological processes” Gene Ontology (GO) terms, and cancer-related gene sets [1, 16, 34–38].

ZNF genes had the highest enrichment for the “znf” annotation (11.0 fold, median *p* < 10^−200^, hypergeometric test, “Methods”). Constitutively unexpressed genes (RPKM < 1 in all 56 cell types with matched expression available) [1] were most enriched in the “quiescent” annotation (5.8 fold, *p* < 10^−300^). In contrast, a set of previously defined housekeeping genes, based on broad and constant expression levels [39], was most enriched in the “strong_trans” annotation (3.0 fold, *p* < 10^−300^), which had a 131 fold depletion for constitutively unexpressed genes. For olfactory genes [34], we observed the strongest enrichment in the “quiescent” annotation (9.2 fold enrichment, *p* < 10^−200^), which contained 75.1% of all olfactory genes.

**Fig. 7:**
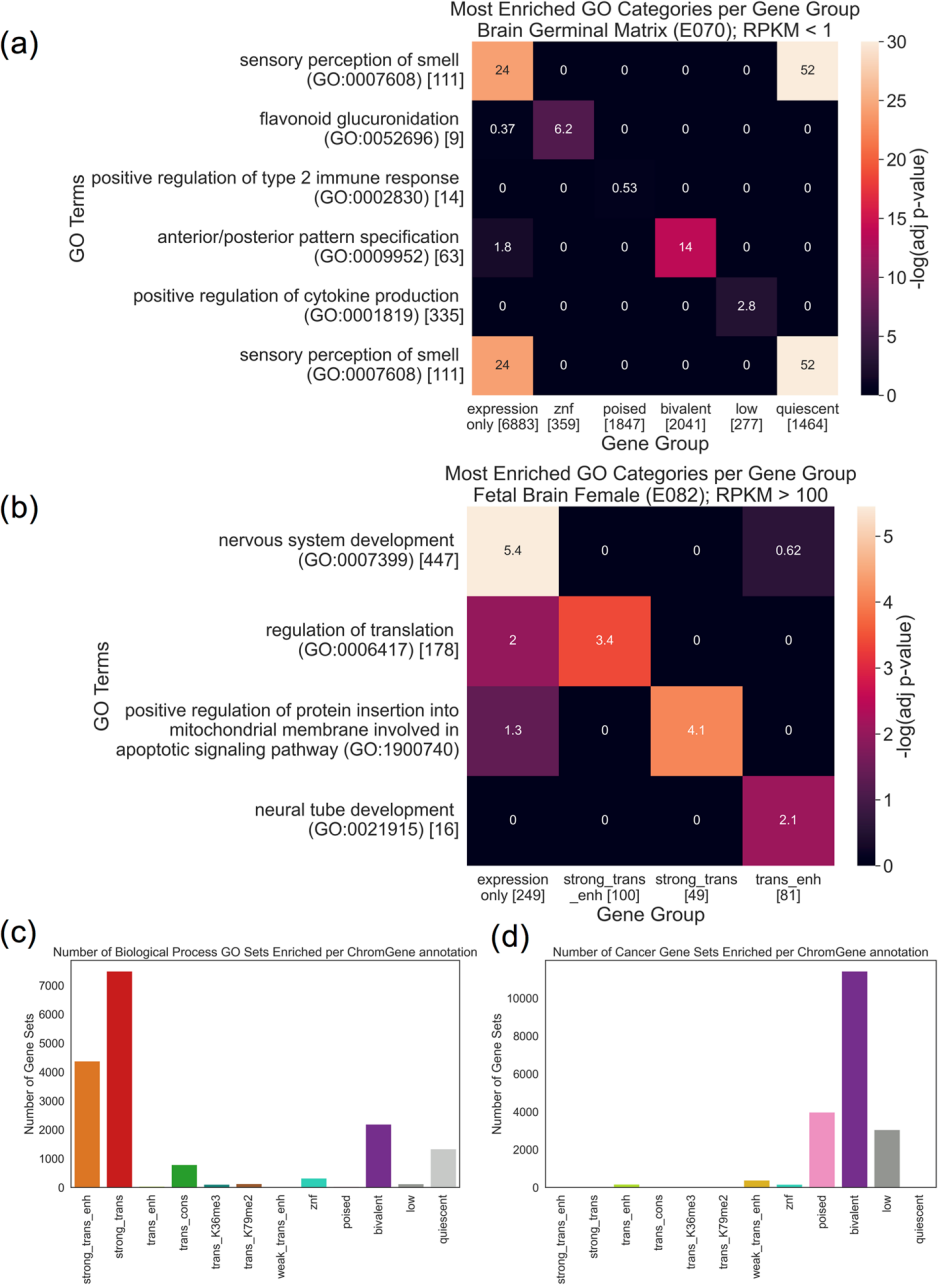
GO term and cancer gene set enrichment. **a** Heatmap showing enrichment −log_10_(*p*-values) of top GO terms (rows) for six gene groups (columns): all “inactive genes” (< 1.0 RPKM), and subsets of those genes assigned to each of five ChromGene annotations associated with lower expression. Asterisks denote significant enrichments after multiple testing correction (“Methods”). Square brackets denote the number of genes in each GO term or gene group. The first row corresponds to the most enriched GO term based on expression only. The second through sixth rows correspond to the most enriched GO term for each of the subsets corresponding to the ChromGene annotations listed in the second through sixth columns, respectively. “Sensory perception of smell” is listed twice since it was most enriched based on both expression only and the subset with the “quiescent” ChromGene annotation. **b** As in **a**, but for highly expressed genes (> 100 RPKM) and using the three ChromGene annotations with the highest overall expression, “strong_trans_enh,” “strong_trans,” and “trans_enh.” **c** Count of how often each ChromGene annotation yielded significant enrichment *p*-values (adjusted *p* < 0.01, Bonferroni corrected for the number of combinations of 127 cell types, 12 ChromGene annotations, and gene sets) across 6036 “biological process” GO term gene sets. **d** As in **c**, but for 967 cancer gene sets

For the GO term enrichments [35], we calculated an adjusted enrichment *p*-value for each GO term gene set for each cell type and ChromGene annotation (hypergeometric test, Bonferroni corrected for the number of combinations of 12 ChromGene annotations and 6036 GO terms). For each ChromGene annotation, we identified GO terms that were enriched in the majority of cell types (adjusted *p* < 0.01) (“Methods,” Additional File 1: Fig. S8a, Additional File 2: Table S1). The ChromGene annotations “strong_trans_enh” and “strong_trans,” which had the highest expressed genes on average, had the greatest number of such GO terms, constituting 26 and 44% of the significant enrichments, respectively (Fig. 7c). The most significant terms for “strong_trans_enh” and “strong_trans” included core regulatory and metabolic processes terms such as “SRP-dependent cotranslational protein targeting to membrane” and “cytoplasmic translation” for “strong_trans_enh” and “mRNA splicing, via spliceosome” and “mRNA processing” for “strong_trans.” The ChromGene annotation “bivalent” had several neuron- and development-related enriched GO terms, including “neuron differentiation” and “anterior/posterior pattern specification.” In contrast, the “quies” term, also associated with low expression, showed significant enrichments for terms such as “sensory perception of smell” consistent with its enrichment for olfactory genes. The different GO enrichments for annotations with similar expression levels suggest ChromGene annotations provide information beyond expression.

To more directly investigate whether ChromGene annotations provides information beyond expression in the context of GO term enrichments, we compared GO term enrichments for gene sets defined based on only gene expression to those defined by both their gene expression and ChromGene assignment. Specifically, we first computed GO term enrichment for unexpressed or minimally expressed genes (“inactive genes”, RPKM < 1.0) in “Brain Germinal Matrix” using the same procedure as above (“Methods”), then found the most enriched term. Then, for each of the ChromGene annotations associated with lower average expression (“znf,” “poised,” “bivalent,” “low,” and “quiescent”), we took the subset of the inactive genes that also had the ChromGene annotation. For each subset, we repeated the GO term enrichment analysis and found the most enriched GO term. We took the most enriched GO term for each of these sets, then analyzed the enrichment *p*-values in all the other sets considered in this analysis (Fig. 7a). We found that the GO term most enriched in inactive genes, “sensory perception of smell (GO:0007608),” was substantially more enriched when subsetting to “quiescent” genes despite having fewer genes in the set (2.5 fold vs 9.1 fold, adjusted *p* < 10^−24^ vs *p* < 10^−52^, Bonferroni corrected, “Methods”). This subset of “quiescent” genes was also enriched in this GO term when using the set of all inactive genes as the background (3.1 fold, adjusted *p* < 10^−24^). Additionally, we found that for each of the other ChromGene annotation subsets, its most enriched GO term had a more significant *p*-value than for all inactive genes. For example, the “bivalent” subset was significantly enriched in “anterior/posterior pattern specification (GO:0009952)” (5.4 fold, adjusted *p* < 1^−14^), consistent with the role of bivalent genes in development [40], but the group of all inactive genes was only marginally enriched (1.8 fold, adjusted *p* = 0.016).

We repeated the analysis with highly expressed genes (RPKM > 100) using the three ChromGene annotations with the highest average expression (“strong_trans_enh,” “strong_trans,” and “trans_enh”), and again found increased significance of notable GO terms when using ChromGene annotations to subset these expressed genes. For example, in the “Fetal Brain Female” cell type, we found the ChromGene annotation “trans_enh” was significantly enriched for “neural tube development (GO:0021915)” (61.5 fold, adjusted *p* < 0.01), but was not significantly enriched using expression only (13.2 fold, adjusted *p* = 1.0) (Fig. 7b). When using a background of highly expressed genes instead of all genes, we observed a marginally significant enrichment for this GO term in “trans_enh” (4.7 fold enrichment, *p* < 0.002).

To determine whether ChromGene annotations provide additional information beyond gene expression regardless of cell type, we compared the number of GO terms enriched when splitting genes by ChromGene assignments to splitting genes randomly. We found that for both the unexpressed (< 1 RPKM) and highly expressed (> 100 RPKM) genes, for all 56 cell types tested, splitting genes by ChromGene assignments yielded more enriched GO terms (mean of 51.6 vs 1.8 for RPKM < 1, mean of 25.1 vs 5.2 for RPKM > 100, *p* < 10^−16^, binomial test) (Additional File 1: Fig. S8b,c, “Methods”). As previous studies have assumed that increased GO significance corresponds to greater biological significance [41, 42], these results would suggest increased biological relevance of using ChromGene annotations. However, we note that it is possible a method’s annotations could yield more significant GO enrichments while not necessarily being more biologically relevant, which would be difficult to determine without inherent ground truth. Together, these results suggest that using ChromGene in tandem with expression information can be used to identify subsets of genes with more specific biological roles than by expression alone. Further, they show that ChromGene can provide useful biological information even in the absence of significant expression.

Across all “biological process” GO terms and all ChromGene annotations, we saw an average of 133 enriched GO terms per cell type (adjusted *p* < 0.01). This was substantially more than for the three baseline methods (TSS, gene average, collapsed), where we saw an average of 53, 109, and 82 gene sets significantly enriched (adjusted *p* < 0.01), respectively. These results demonstrate that ChromGene annotations yield more “biological process” GO term enrichments than the baseline methods.

We next took 967 cancer-related gene sets, produced by the Cancer Cell Line Encyclopedia and implicated in various types of cancer [37], and, as before, performed a gene set enrichment analysis for each ChromGene annotation. Interestingly, we found that unlike for the “biological process” GO terms, significant enrichment for cancer gene sets was most likely to be found in lowly expressed annotations, particularly “poised,” “bivalent,” and “low,” which constituted the majority of enrichments, with 21, 60, and 16%, respectively (Fig. 7d). The enrichment of the “poised” and “bivalent” annotations is consistent with previous observations that misregulation of poised and bivalent chromatin regions are associated with cancer [43, 44]. In total, we found that ChromGene had an average of 151 cancer gene sets enriched per cell type. In comparison, the baseline methods (TSS, gene average, collapsed) had 71, 72, and 70 gene sets enriched (hypergeometric test, *p* < 0.01, Bonferroni corrected for the number of combinations of 127 cell types, 12 annotations, and 967 gene sets), respectively, substantially less than for ChromGene. These results further support that ChromGene annotations are more consistent with established cancer gene sets than the baseline approaches.

### ChromGene separates genes by pLI scores

We next explored the relationship between ChromGene annotations and gene constraint, specifically those with high pLI scores (≥ 0.9) [25]. Genes with high pLI scores have strong evidence that the gene is intolerant to loss of function, i.e., haploinsufficient.

**Fig. 8:**
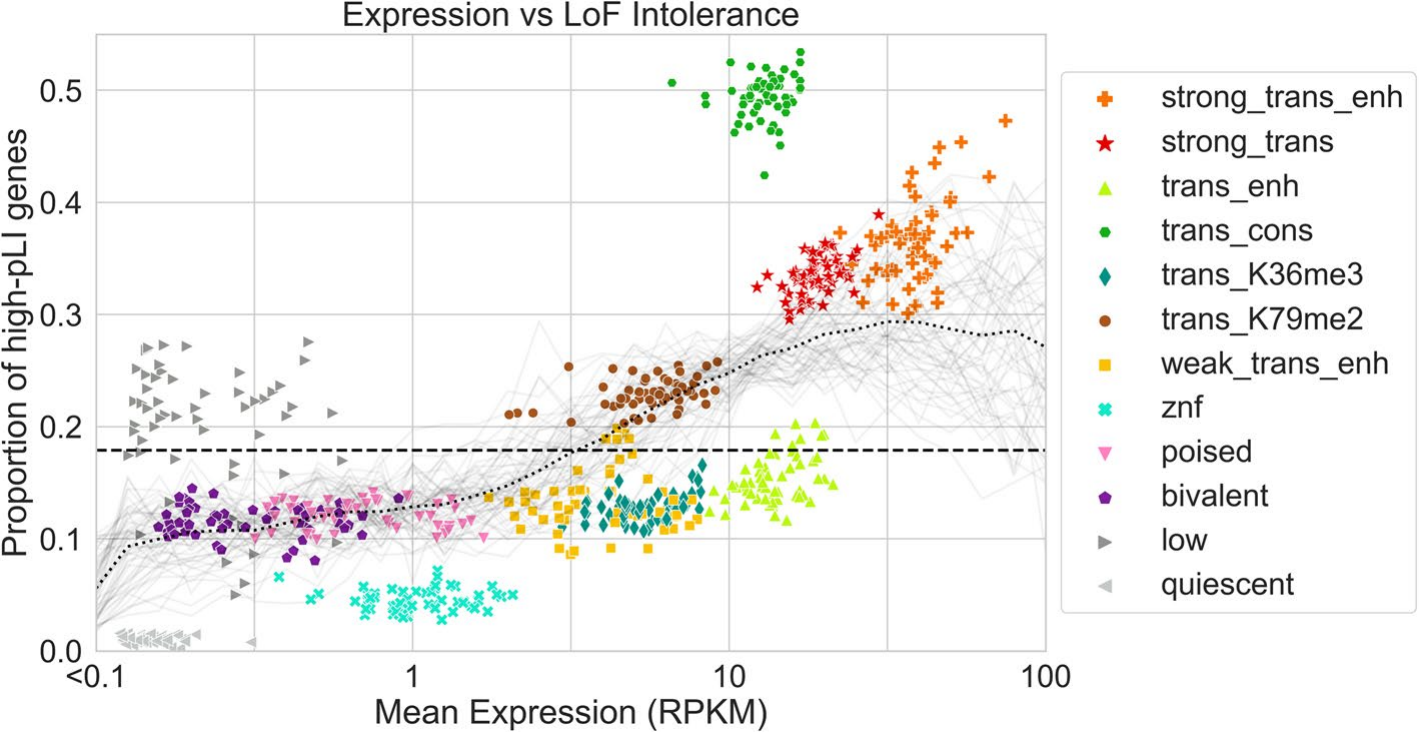
Expression and pLI scores per ChromGene annotation. Scatter plot showing gene expression (RPKM) on the *x*-axis and the proportion of high-pLI genes (pLI ≥ 0.9) on the *y*-axis. Each point corresponds to genes assigned to a specific ChromGene annotation in a single cell type. Points are colored by their ChromGene annotation. The dashed line corresponds to the overall proportion of genes with high pLI. Gray curves represent the proportion of high-pLI genes as a function of expression, one per cell type. The dotted black line is the proportion of high-pLI genes, averaged across cell types, as a function of expression (“Methods”). The figure shows that although there is a positive association between the two measures, ChromGene captures information beyond expression. For example, “trans_enh” and “trans_cons” have similar expression levels, but substantially different proportions of high-pLI genes

The three ChromGene annotations most enriched for high pLI score genes were “trans_cons,” “strong_trans_enh,” and “strong_trans” (Fig. 8), with an average of 49.6, 35.8, and 33.3% of their assigned genes having high pLI scores across cell types, respectively, compared to 18.1% expected by chance. These three annotations were also among the four annotations associated with the highest expression.

Notably, “trans_cons” had a substantially larger percentage of high-pLI genes (49.6%) than “trans_enh” (14.7%), despite a positive correlation between mean ChromGene annotation pLI score and expression (Spearman *r* from 0.18 to 0.39, mean *r* = 0.27 across cell types, all *p*-values < 10^−100^, “Methods”), and “trans_cons” having slightly lower overall expression than “trans_enh” (median RPKM = 12.73 and 13.89, respectively). This difference is consistent with the substantial difference in gene length between the “trans_cons” and “trans_enh” annotations (Fig. 5), and a previously noted positive correlation between gene length and pLI [25]. We still saw a difference in percentage of high-pLI genes between “trans_cons” and “trans_enh” after correcting for gene length, although it did decrease, with 28.8 and 20.9%, respectively, of annotated genes having high pLI scores (“Methods,” Additional File 1: Fig. S9, Additional File 2: Table S1). Another difference between “trans_cons” and “trans_enh” is that genes annotated as “trans_cons” are more likely than “trans_enh” to be assigned to the “strong_trans” or “trans_K79me2” annotations in other cell types (Fig. 6, Additional File 1: Fig. S5b).

Among the lowly expressed ChromGene annotations, “low” and “quiescent” had the greatest difference in proportion of high-pLI genes (21.7 and 1.0%, respectively), although this difference was smaller after correcting for gene length (8.9 vs 2.9%, respectively) (“Methods”). Compared to the “quiescent” annotation, the “low” annotation contained longer genes and genes more likely to be assigned in other cell types to ChromGene annotations with a greater proportion of high-pLI genes. Similar patterns also held when considering mean pLI (Additional File 1: Fig. S10). These results further show that ChromGene captures additional information beyond expression.

## Discussion

Here, we introduced ChromGene, a principled, model-based method that uses a mixture of HMMs to annotate genes based on maps of multiple epigenomic marks across genes. ChromGene’s focus on gene assignments is complementary to well-established approaches for generating per-position assignments [9, 10].

We applied ChromGene to imputed data for 12 epigenomic marks to annotate genes in over 100 cell types. We showed that ChromGene annotations frequently reflect distinct gene expression levels. In cases where ChromGene annotations had similar gene expression levels, we found they differed on other important properties such as gene set enrichments, including for high pLI score genes, reflecting that ChromGene annotations capture information beyond gene expression levels. We also showed that ChromGene yielded better agreement with gene expression data and more significant enrichments for cancer gene sets and GO terms than baseline approaches.

As ChromGene directly assigns genes to annotations, it will likely be preferable for many gene-centric analyses relative to per-position chromatin state assignments. While ChromGene annotations do correspond to patterns previously identified by per-state annotations, ChromGene annotations are more expressive in that they can associate multiple per-position patterns with the same gene-based annotation. Relating per-position annotations directly to genes has often involved using post hoc approaches mapping these annotations to individual genes. However, such approaches are subject to loss of information and can be biased by gene length. ChromGene mitigates these issues by directly providing a model-based annotation of individual genes. We note that ChromGene annotations are less likely to have advantages over per-position annotations when the object of study is not inherently gene-centric, such as studying enrichments for GWAS-identified variants.

There are multiple possible additional types of applications and extensions of ChromGene that can be investigated in future work. Although here, we applied ChromGene to protein-coding genes, ChromGene is more general and can also be applied to other pre-defined sets of genomic intervals such as long non-coding RNAs and pseudogenes. In this study, we combined our training data in a “concatenated” mode, where chromosomes across different cell types are treated as separate, and a single model is trained that applies to each epigenome. However, the input data for ChromGene can also be “stacked,” where the emissions of marks across cell types are all observed simultaneously [28, 45]. This can be used to annotate genes with patterns of variation learned across cell types. In this study, we applied ChromGene to a set of 12 imputed data sets to have a comparable set of input features across many cell types. However, ChromGene can be applied with other choices for the input data. In this study, we did not analyze ChromGene’s per-position annotation of genomic bins to individual mixture component hidden states, as we see the gene-level annotations as the main value for ChromGene relative to existing per-position annotations. However, investigating the use of ChromGene’s per-position annotations for specific applications could be a direction for future work.

## Conclusions

ChromGene generates gene-level annotations, analogous to chromatin states at the position level, by modeling the combinatorial and spatial pattern of epigenomic marks using a mixture of HMMs. We used ChromGene to assign nearly 20,000 protein-coding genes for over 100 cell types to twelve gene-level chromatin annotations. We have shown that these annotations not only capture variation in gene expression, but also provide additional information. The assignment of genes to ChromGene annotations, along with the trained ChromGene model and software we used to generate them, are publicly available [30, 31]. We expect that the ChromGene assignments we have generated will be a useful resource for gene-based analysis in and across many cell types and that the approach will be useful for gene-based annotations for data from additional marks, cell types, or species.

## Methods

### ChromGene model

For a single cell type, ChromGene uses a mixture of multivariate HMMs to model the combinatorial and spatial patterns within multiple epigenomic maps across a set of genes to derive a single assignment per gene. Each gene is assumed to be generated by one of *M* fully connected HMMs, each with *S* states. Each of these *M* HMMs is defined by three sets of parameters: (1) the initial probability for each state within the HMM, (2) the probability of transitioning from one state to another state in the same HMM, and (3) the probability of observing each of *E* binarized epigenomic mark emissions given a state in the HMM. Although not explicitly trained, each HMM’s prior probability of being chosen for a gene is equal to the sum of the initial probabilities of its states.

More formally, we denote mixture components as *m*, *1* ≤ *m* ≤ *M*, where *M* is the total number of components; we denote states as *s*, *1* ≤ *s* ≤ *S*, where *S* is the number of states per component. The total number of states is thus $M\times S$, and each hidden state is denoted *h*_*m,s*_. The initial probability, transition probabilities, and emission probabilities are defined as follows:

### Initial probabilities

The initial probability for each state, denoted ${\tau }_{m,s}$, corresponds to the probability that a randomly chosen gene starts in state *h*_*m,s*_. The sum of all the initial probabilities of states in a mixture component *m* corresponds to the initial probability of a gene starting in component *m:*${\tau }_{m}={\sum }_{s=1}^{S}{\tau }_{m,s}$ and thus the prior probability of the gene being assigned to component *m*.

### Transition probabilities

Each mixture component is modeled as a fully connected HMM. The transition parameters ${\alpha }_{ms,ms{\prime}}$ correspond to the probability of a state *s* within component *m*, *h*_*m,s*_, transitioning to a state $s'$ within the same component $m,h_{m,s'}$. Transitions from states within one component to states in another component are not allowed.

### Emission probabilities

The probability of observing a specific combination of marks in a state *s* of component *m*, *h*_*m,s*_, is calculated based on the product of independent Bernoulli random variables, with emission parameters ${\beta }_{m,s,e}$, over all marks following the approach of ChromHMM [9]. ${\beta }_{m,s,e}$ and $(1-{\beta }_{m,s,e})$ represent the probability of observing mark *e* as present or not present at a specific position in state ${h}_{m,s}$, respectively.

To apply ChromGene to multiple cell types, we apply the same modeling approach but treat data from multiple additional cell types as if they were additional genes in one cell type. This leads to a common model across cell types, but with cell type-specific gene assignments. This approach is analogous to the “concatenated” model learning approach of ChromHMM [2, 28].

### Model learning

#### Parameter initialization

The initial parameters of states across all mixture components are initialized to sum to 1 using a Dirichlet distribution with ${\alpha }_{m,s}=1$. The emission parameters are initialized for each state by first randomly assigning each gene to one of the *M* components. Then, each position in the genic region and flanks is assigned uniformly at random to one of the *S* states within that component. Finally, for each of the $M\times S$ hidden states, we set the initial emission probability of each epigenomic mark to its average frequency of being observed present across all positions assigned to the state. For each component *m* and state *s* in the component, the transition parameters from the state to each state in its component are initialized to sum to 1 using a Dirichlet distribution with ${\alpha }_{s}=1$.

#### Expectation–Maximization procedure

After initializing parameters, ChromGene trains the parameters iteratively with the Baum-Welch algorithm, a special case of the Expectation–Maximization (EM) algorithm, until convergence. In the E step, ChromGene takes the current parameter values (emission, transition, and initial probabilities) and calculates the log likelihood of the data being observed given those values, $\mathrm{log}(L(\theta ;X,H))=\mathrm{log}(p(X,H|\theta ))$, where $\theta =\{{\theta }_{\tau }, {\theta }_{\alpha }, {\theta }_{\beta }\}$, the set of initial, transition, and emission parameters, respectively, *X* is the observed data, and *H* is the set of true, but hidden state assignments. In this step, ChromGene calculates the posterior probability of each position of each gene being generated by each of the $M\times S$ hidden states.

In the M step, these posterior probabilities are used to re-estimate the parameters $\theta$ so that the new parameter values maximize the expected likelihood in the E step, i.e., ${\theta }^{t+1}={\text{argmax}}_{\theta }{E[}_{H|X, {\theta }^{t}}log(L(\theta ;X,H))]$. In our application, we trained for 200 iterations, as is default in ChromHMM, and parallelized the E step computation across 8 cores using the option `-p 8`. For faster computation, we also only evaluated on a randomly selected subset of the data in each iteration.

### Implementation on top of ChromHMM

To implement ChromGene on top of ChromHMM, we first noted that a mixture of HMMs can be defined in terms of a single HMM with certain transitions disallowed. Specifically, a set of *M* fully connected HMMs, each acting as a mixture component, can be represented with a single, larger HMM, and multiple genes within the same file can be handled with the addition of a “dummy state” and “dummy mark.” In this single HMM, each non-dummy state of the HMM can transition into and out of other states within its component and a single dummy state, but not directly to states of other HMM components (Fig. 1a,b). From the dummy state, transitioning to any component is allowed, but only through the use of the dummy mark, which forces transitions into and out of the dummy state.

We structured the input data so that all genes in a chromosome and cell type (after adding 2-kb flanks on both ends and reversing genes on the negative strand) were concatenated head to tail, but with a single “dummy position” separating the genes, and a dummy position at the beginning and end of the data. In this dummy position, the emission for all the original input marks is set to 0, but we include an emission of 1 for a single, new mark designated the “dummy mark” (Fig. 1c). This dummy mark has an emission of 0 within all extended genic regions, and 1 only for the dummy position (Fig. 1d). Upon reaching the dummy position, the model forces transition to the dummy state. From this dummy state, transition to any of the mixture components is allowed.

This structure also allowed us to compactly represent all genes without having separate files for each gene and cell type. For a single cell type, we had 23 chromosome files (chromosomes 1–22 and X). For 127 cell types, this yielded 127 × 23 = 2921 total input data files. We note that in theory, we could apply an equivalent model without dummy marks and states using ChromHMM by creating a separate file for each combination of gene and cell type. However, such a process for our training scenario would produce approximately 2.5 million files, which can be over system limits for the number of files.

We generated initial parameter files for the initial parameters described above. For *M* mixture components, *S* states per component, and the dummy state, we had a total of (*M* × *S*) + 1 states. The initial probability for each state in each component is set to 0, except for the dummy state, which is set to 1. The transition probabilities from any state in a component to each other state in the same component was allowed to be nonzero and normalized so that the sum of these probabilities was 0.95. The remaining 0.05 probability was set to be the probability of transitioning from the component’s states to the dummy state. The probability of transitions from the dummy state to each of the other *M* × *S* states corresponds to the initial parameters, which, as described above, were initialized using a Dirichlet distribution with ${\alpha }_{m,s}=1$ for all components so that they summed to 1. The transition probability from a state in a component to each state in different components was set to 0.

Finally, the emission probabilities for the non-dummy marks in the non-dummy states were initialized as described above. For the dummy state, the emission probability of the epigenomic marks was set to 0, and the emission of the dummy mark was set to 1; in the non-dummy states, the emission probabilities for the dummy mark was set to 0. All input files were generated using the script `generate_chromgene_input_files.py` with default parameters [30, 31].

We passed the data and the initialized parameter files into ChromHMM’s `LearnModel` function and used several optional flags. We used the `-e 0` and `-t 0` flags to enforce that a 0 or 1 emission or transition parameter in the initial parameter file remains at that value. We also used the `-scalebeta` flag to increase numerical stability in our setting (below, “Handling numerical stability issues”). We used the flag `-n 100` to sample 100 files for each iteration, and `-d -1` to allow the log likelihood to increase between iterations. The log likelihood can increase between iterations because different subsamples of the data are evaluated on different iterations. We trained for 200 iterations, as is default in ChromHMM. We used version 1.18 of ChromHMM.

After training the model, we used ChromHMM to generate posterior probability assignment files. We used these files to calculate the posterior probability of each of the *M* HMMs of generating each gene in each cell type; finally, we took the HMM with the highest posterior probability to derive hard assignments. These assignments were generated using the script `chromgene_posteriors_to_components.py` with default parameters [30, 31].

### Handling numerical stability issues

Implementing a mixture of HMM raised some numerical stability issues not encountered with standard, single HMMs in ChromHMM. In this setting, it is expected that for some genes, there will be specific individual HMMs that would have essentially zero probability of generating the observations for the gene. Previously, to deal with numerical stability issues, ChromHMM used a single approach to scaling, which was to rescale both the forward (alpha) variables of the HMMs and the backward (beta) variables at a specific position by the sum of the forward variables. This followed previous standard presentations of HMMs [46, 47]. However, this scaling approach led to numerical overflow in our setting. Namely, the magnitudes of the backward variables at a specific position can be vastly greater than the sum of the forward variables, and this difference can be continued to be amplified over long sequences, which in some cases can lead to numerical overflow. This likely happens specifically in this setting because the forward variables for a sequence can be dominated by one mixture component while the backward variables for a sequence can be dominated by another mixture component. To handle the overflow, we rescaled the backward variables based on the sum of the backward variables, which is valid as previously noted [48].

While rescaling the backward variables by the sum of the backward variables eliminated the numerical overflow, numerical underflow issues were then encountered. Specifically, situations were encountered in certain cases for individual genes where the product of each forward and backward variable for every state was 0, which would lead to division by 0 and no meaningful overall output. This also likely happens in certain situations in which one mixture component dominates the forward variables and another one the backward variables. To avoid the numerical underflow, if a backward variable for a state at a position fell below 10^−300^, we set its value to 10^−300^. We did the same for forward variables, except if the emission probability product for a state at the position was actually 0, in which case the forward variable was kept at 0. These changes were sufficient to lead to a numerically stable procedure.

This alternative procedure for handling numerical stability can now be accessed in ChromHMM by using the `-scalebeta` flag.

### Hyperparameter selection

In ChromGene, there are two hyperparameters that must be set: *M*, the number of mixture components, and *S*, the number of states per component. For *M*, the number of mixture components, we considered values ranging from 8 to 20 in increments of two. For *S*, the number of states per component, we considered each value ranging from 2 to 5, while *S* = 1 corresponded to the gene average baseline.

The choice of *M* trades off the ability of the model to capture additional classes of genes while maintaining meaningful distinctions between each annotation. To assess this trade-off, we calculated ChromGene’s reproducibility, defined as the percentage of identical assignments in cell types that can be considered replicates (below, Calculation of confusion matrix and contingency table), as a function of *M* and *S*. We found that reproducibility is more sensitive to the choice of *M* than *S* (Additional File 1: Fig. S1a). We found that there was a large drop in reproducibility between *M* = 12 mixture components and 14 or more. On the other hand, the drop in reproducibility from *M* = 8 and *M* = 10 to *M* = 12 was relatively minimal, suggesting 12 mixture components could provide a reasonable trade-off for the expressivity of the model without having any pair of mixture components that are largely redundant (Additional File 1: Fig. S1b).

The choice of *S* trades off the expressivity of the state representation within a mixture component with the overall interpretability of each mixture component. A mixture component with too many states could have “redundant states,” where two or more states have very similar emission parameters. To investigate this trade-off, we calculated the mean Manhattan distance between the emission parameters of the two closest states within a mixture, for values of *M* = 8 to 20, and *S* = 2 to 5 (Additional File 1: Fig. S1c). We note that *S* = 1 is excluded from this analysis because two states are needed to calculate a distance. Unlike with reproducibility, this metric was more sensitive to the choice of *S* than *M*. We found that the mean Manhattan distance in models using *S* = 4 or *S* = 5 states per mixture have a similarly low value, suggesting that these models have pairs of “redundant” states that are not well-differentiated by emission parameters (Additional File 1: Fig. S1d). However, there is still an appreciable gap between *S* = 3 and both *S* = 4 and *S* = 5, supporting the choice of *S* = 3 to trade off model expressivity while maintaining a relatively interpretable model with limited redundancy between sub-states of the model.

### ChromGene annotation visualization

To visualize ChromGene assignments across cell types and genes, we first randomly subsampled 2000 of the 19,919 protein-coding genes. For each pair of these genes, *i* and *j*, we calculated a pairwise distance between them as $1-\mathrm{mean}\left(\mathrm{I}\left({m}_{i,c}={m}_{j,c}\right)\right)$, where *m*_*g,c*_ corresponds to the ChromGene assignment for gene *g* in cell type *c*, I(*x*) is the indicator function, and the mean is across the 127 cell types. Columns, which correspond to cell types, were ordered the same way as previously [1]. Rows were ordered using scipy’s hierarchical clustering function, using optimal leaf ordering and average (UPGMA) linkage.

### Baseline model implementations

#### TSS model

To generate a 12-component model based only on gene TSSs, we took epigenomic mark binarization data across the 127 cell types for the 19,919 genes and used the data at the 200-bp bin overlapping the TSS position. As with the standard ChromGene input data, we separated these individual TSS positions by a single dummy position, then trained a 12-component model with a single state per component (*M* = 12, *S* = 1) on top of ChromHMM and followed the same procedures as above for generating assignments.

#### Gene average model

The gene average model was implemented using ChromGene using the standard procedures as above, except that for the *M* = 12 mixture components, it used a single hidden state per mixture component (i.e., *S* = 1 states per component).

#### Collapsed model

The collapsed model was identical in structure to the gene average model (*M* = 12 mixture components, *S* = 1 states per component), but was not trained directly. After training the full ChromGene model (*M* = 12 components, *S* = 3 states per component), as is used in all results presented, we calculated the prior probability of a “collapsed” component by summing the initial probabilities of states within the corresponding component of the full model. If the initial probabilities for the *S* states within a component *m* are denoted ${\tau }_{m,s}$, then the initial probability for the collapsed state $s'$ of the corresponding component $m'$ is ${\tau }_{s{\prime}}={\pi }_{m}=\sum_{s=1}^{S}{\tau }_{m,s}$, where ${\pi }_{m}$ is the empirical prior probability of component *m*. The transition probability from the dummy state to a component is this prior probability. The transition probability of the collapsed state to itself is calculated by comparing the number of observed state assignments for the corresponding component in the full model to the number of genes assigned to that component. If the number of 200 bp positions assigned to some component is denoted *x*_*m*_, and the number of genes assigned to that component is denoted *n*_*m*_, then there are (*x*_*m*_ − *n*_*m*_) transitions from the component to itself and *n*_*m*_ transitions from the component to the dummy state, for a total of *x*_*m*_ transitions. We set the transition probability of the collapsed state to itself as (*x*_*m*_ − *n*_*m*_) / *x*_*m*_ and the transition probability of the collapsed state to the dummy state as *n*_*m*_ / *x*_*m*_.

The emission probability parameters of the collapsed model are set to the weighted mean of the probabilities of the full model, weighted by the state prior probabilities. If the emission probability for a state *s* in component *m* for mark *e* is denoted ${\beta }_{m,s,e}$, the emission probability of the corresponding collapsed component $m'$ with the single collapsed state $s'$ is ${\beta }_{{m}{\prime},{s}{\prime},e}=\sum_{s=1}^{S}{\pi }_{m,s}{\beta }_{m,s,e}$, where ${\pi }_{m,s}$ is the empirical prior probability of state *s* in component *m*, which is equal to the fraction of 200-bp bins assigned to state *s* across all cell types and genes. These parameters were all set in the ChromHMM model file and were used directly without training to generate assignments for all genes and cell types.

### Assignment of genes to mixture components

After training, ChromGene assigns each position along a gene a posterior probability of being in each of the hidden states $p({H}_{m,s}|X)$, where *H*_*m,s*_ is the assignment to mixture component *m* and hidden state *s*, and *X* is the observed data. From this, ChromGene calculates the posterior probability of a gene being generated by each mixture component: $P(m|X)={\sum }_{s=1}^{S}p({H}_{m,s}|X)$ based on any position. We note that all positions within a gene have the same component posterior probabilities; for example, if the first position of a gene has a posterior probability of 0.4 of being generated by component 1, then all positions within the gene have a posterior probability of 0.4 for component 1. This is a consequence of transitions between components being disallowed. ChromGene assigns each gene to the component $1\le {m}^{*}\le M$ with the highest total posterior probability of generating the gene, i.e., ${m}^{*}={\text{argmax}}_{m\in M}P(m|X)$. We used these hard assignments of genes to components for all presented results.

### Training data

To generate input data for ChromGene, we first defined our genes of interest. For this application, we used 19,919 protein-coding genes as defined by Ensembl v65 / GENCODE v10 for hg19, as the epigenomic data we used had matching gene-level expression estimates across 56 cell types based on these gene annotations. For each gene, we took the TSS and TES of the gene as previously defined [1, 16, 49]. Genes on the negative strand were reversed to align with genes on the positive strand so all genes had the same orientation in the model. We rounded the TSS upstream (in the 5′ direction) and the TES downstream (in the 3′ direction) to the next position divisible by 200 bp. We added an additional flank of 2 kb upstream of the TSS and downstream of the TES to capture additional spatial information around the TSS and TES. We then binned the entire region (gene and flanks) into 200-bp bins so that the boundaries of each bin were divisible by 200. Overlapping genes were considered separately with their overlapping regions repeated. We then extracted the epigenomic marks, as described next.

To generate a single ChromGene model that would be comparable across a large number of cell types and marks, we used imputed data for a set of 12 marks (H3K36me3, H3K4me1, H3K4me2, H3K4me3, H3K9me3, H3K27ac, H3K27me3, H3K9ac, H3K79me2, H4K20me1, H2A.Z, and DNase) across 127 reference epigenomes [1, 27], which for our purposes we treated and referred to as different cell types, except for calculation of the confusion matrix and contingency table, as described below. This imputed data was previously used to train a 25-state ChromHMM model [27]. The use of imputed data allowed us to annotate more cell types with the same set of marks compared to using directly observed data. We used the same binarization for the imputed data as previously generated [27, 50].

### Gene expression analysis

We downloaded gene expression data for 56 cell types from the Roadmap Epigenomics Consortium [1, 51]. We took the provided RPKM values, added a pseudocount of 0.1, and took the log_10_ transform of the value.

To determine whether median gene expression variance within annotations was significantly smaller than across annotations, we performed a permutation test. Each median log_10_(RPKM + 0.1) expression value for a cell type was randomly assigned to one of the ChromGene annotations, while maintaining 56 cell types per ChromGene annotation. We then calculated the mean within-annotation variance of the median expression values for each permutation and for the true ChromGene annotation. To compute a *p*-value, we counted the fraction of 10,000 permutations in which the mean variance based on the permuted values was less than that of the true ChromGene annotations.

To assess if there was a significant difference in ChromGene’s AUROCs for predicting expressed genes to those of the baseline methods, we used a one-sided binomial test, where we counted how often the AUROC for ChromGene was higher than that of the baseline method, where each observation is a cell type. Based on these counts, we calculated a *p*-value, where the null hypothesis is that the baseline method is more likely to produce the higher of the AUROCs, *p* = 0.5, and *n* = 56 cell types with expression data as observations.

### Calculation of mutual information of assignment given gene length

To calculate mutual information of gene assignments given gene lengths, for each gene, we first calculated its log_10_(*length*), which ranged from 2.06 to 6.73, where *length* is in bp. We binned the log_10_(*length*) into 0.05-increment intervals to create a discretized distribution. Then, for each cell type, we calculated the mutual information of ChromGene assignment and the length bins. We repeated the procedure for each baseline method. To compare to the baseline methods, we used the binomial test described above, using mutual information instead of AUROC and *n* = 127 cell types.

### Calculation of confusion matrix and contingency table

To generate a confusion matrix (Additional File 1: Fig. S5a), we took our matrix of ChromGene assignments with entries *m*_*g,c*_, where each of *g* = *1,…,19919* corresponds to a gene and each of *c* = *1,…,127* corresponds to a cell type (Fig. 2, genes subsampled and ordered for visualization). For each gene *g*, we calculated the conditional probability *P(m*_*g,c*_* | m*_*g,c’*_*)*, where $c\ne c{\prime}$ correspond to pairs of epigenomes that were originally annotated as the same cell type but of different individuals (“Rectal Mucosa Donor 29” and “Rectal Mucosa Donor 31”, “Foreskin Fibroblast Primary Cells skin01” and “Foreskin Fibroblast Primary Cells skin02”, “Foreskin Melanocyte Primary Cells skin01” and “Foreskin Melanocyte Primary Cells skin03”, “Foreskin Keratinocyte Primary Cells skin02” and “Foreskin Keratinocyte Primary Cells skin03”, “Skeletal Muscle Female” and “Skeletal Muscle Male”, “Primary hematopoietic stem cells G-CSF-mobilized Male” and “Primary hematopoietic stem cells G-CSF-mobilized Female”, “Fetal Brain Female” and “Fetal Brain Male”). In short, we sought to answer the question: “given an entry in the assignment matrix is mixture component *i*, what is the probability that the same gene is assigned to component *j* in a replicate cell type?” We then calculated overall conditional probabilities by averaging over all genes *g*. We represented these conditional probabilities so that the component conditioned on corresponds to a row and each row sums to 1.

To generate the gene contingency table (Additional File 1: Fig. S5b), we performed the same process as for the confusion matrix, but instead of calculating probabilities for pairs of “replicate” cell types, we calculated them for non-replicate cell types.

### Cell type specificity

We defined the “cell type specificity” of each ChromGene annotation by dividing the diagonal of the contingency table (across non-replicate cell types) by the confusion matrix diagonal, then subtracting this value from 1 to obtain a “cell type specificity score” so that higher values correspond to more cell type-specific annotations.

### Calculation of co-assignment matrix enrichment

To generate the co-assignment matrix enrichment (Fig. 6), we first generated an expected co-assignment matrix, where we assumed independence between assignments. For each ChromGene annotation, *m*, we calculated an empirical prior probability of observing the annotation assignment for a random cell type and gene ${\pi }_{m}=\mathrm{P}(m)={\sum }_{c=1}^{127}{\sum }_{g=1}^{19919}\mathrm{I}({m}_{g,c}=m)/(127\times 19919)$, where ${\mathrm{I}(m}_{g,c}=m)$ denotes the indicator function applied to the ChromGene assignment for gene *g* in cell type *c* being *m*. We took these prior probabilities and calculated an expected co-assignment matrix, where each entry (*i, j*) was found by multiplying the prior probabilities of mixture components *m*_*i*_ and *m*_*j*_: P(*m*_*i*_) × P(*m*_*j*_). We calculated the observed co-assignment matrix by counting the frequency with which a gene was assigned to component *m*_*i*_ in one cell type and *m*_*j*_ in the other, averaging over all genes and all pairs of cell types that were not considered replicates. We then normalized this matrix by the sum of its values to form a probability distribution. To calculate enrichments, we divided the observed co-assignment matrix (Additional File 1: Fig. S5b) by the expected co-assignment matrix. Finally, we took the log_2_ of these values to show enrichments and depletions.

### Calculation of differences in gene expression as a function of ChromGene assignment

To calculate expression changes as a function of changes in ChromGene expression, we first took all 19,919 genes across the 56 cell types with expression quantified. For each pair of distinct cell types *i* and *j*, we took each gene’s expression and assignment in cell types *i* and *j*, then divided the expression of the gene in cell type *j* by that of its expression in cell type *i*. We then put this value in a bin indexed by the gene’s ChromGene assignment in cell types *i* and *j* (a total of 12 × 12 bins). Finally, we took the mean of the log_2_ expression ratios in each bin and plotted the result as a heatmap (Additional File 1: Fig. S7a). Additionally, we plotted the mean expressions of the genes in cell type *j* conditioned on the assignment in cell type *i* (Additional File 1: Fig. S7d), which are the denominators of the ratios used in Additional File 1: Fig. S7a.

### ChromGene assignments in hg38-based gene annotations

As the Roadmap Epigenomics data and gene annotations (v10) were based on the hg19 assembly, we used the same assembly and gene annotation for all analyses here [1, 16]. However, we also generated ChromGene assignments by applying our previously trained ChromGene model with the lift over of a more recent hg38-based protein-coding gene annotation. Specifically, we used the version of the hg38-based GENCODE v41 annotation that had previously been lifted over to hg19 [16, 52]. We found that when comparing common genes based on gene name, ChromGene assignments were 93.7% concordant. The 6.3% that were not concordant were due to differences in the annotated TSS or TES position; 46% of discordant genes (total of 2.9%) had a start or end position that was more than 10 kb away between the two annotations.

### Olfactory and housekeeping gene annotations

We downloaded olfactory [34] and housekeeping gene annotations [39]. We matched the gene names to the GENCODE annotation [16] to label each gene as olfactory or not olfactory and as housekeeping or not housekeeping.

### ChromHMM state enrichment

For each ChromGene mixture component, we took all genes and cell types assigned to the mixture component, and counted the total observed counts of each ChromHMM state in a previously described 25-state model [27], thus yielding a matrix of *M* ChromGene components by 25 ChromHMM states. We then added a pseudocount of 1 to these counts and normalized the rows (ChromGene components) to unit probability. We divided these probabilities by the genome-wide ChromHMM state assignment proportions across all cell types to generate an enrichment, and finally, calculated the log_2_ of these enrichments (Additional File 1: Fig. S6).

### Gene set enrichments

To calculate the fold enrichments for ZNF-named genes, housekeeping genes, constitutively unexpressed genes (RPKM < 1 in all 56 cell types with matched expression available), constitutively expressed genes (RPKM > 1 in all 56 cell types with matched expression available), and olfactory genes for a mixture component, we divided the mean proportion of genes from the set assigned to the component across all cell types by the empirical prior probability of a gene being assigned to the component. To calculate a median *p*-value for a gene set and component across cell types, we took each cell type and calculated a *p*-value using a hypergeometric test (`scipy.stats.hypergeom`, with *X* = [number of genes in component and gene set], *M* = [total number of genes], *n* = [total number of genes in gene set], *N* = [number of genes in component]), and then took the median of those values.

To calculate significant gene set enrichments for “biological process” GO terms [35] (Fig. 7), we first found the overlap of a gene set with each of the ChromGene annotations for a given cell type. Next, we calculated *p*-values using a hypergeometric test, as for the individual gene sets above. The heatmap in (Fig. 7a,b) shows unadjusted *p*-values, marked with an asterisk when significant after accounting for multiple testing with a Bonferroni correction. For the “expression only” column, we controlled only for the number of GO terms tested, while in the remaining columns, signifying expression and some ChromGene annotation, we controlled for both the number of GO terms and ChromGene annotations tested. We chose to test the minimal set of ChromGene annotations that contained at least 75% of tested genes, starting with the lowest expressed annotation for RPKM < 1 and highest for RPKM > 100. In Fig. 7c, the bar chart shows the number of gene sets significantly enriched after correcting for the number of GO terms, ChromGene annotations, and cell types (adjusted *p* < 0.01).

We calculated GO term enrichments for each baseline method following the process above and repeated the process for cancer gene sets (Fig. 7d) [37]. The gene sets for “biological process” GO terms and cancer gene sets were downloaded from the Enrichr database [38].

To generate the GO term enrichment median *p*-value heatmap (Additional File 1: Fig. S8a), we followed the procedure above, but without correcting *p*-values for the number of cell types. We removed rows where the median adjusted *p*-value was greater than our significance threshold of 0.01, and clustered rows using `seaborn.clustermap`, which uses a Euclidean metric and `average` linking. Finally, we − log_10_ transformed the *p*-values for visualization.

To compare the number of GO terms enriched when splitting unexpressed (or highly expressed) genes by ChromGene annotation to splitting randomly (Additional File 1: Fig. S8b,c), we first took the set of all genes with < 1 RPKM (or > 100 RPKM). As above, we then took the minimal set of ChromGene annotations, ordered by expression, that contained at least 75% of the tested genes as the annotations tested, and for each, calculated gene set enrichments for all GO terms. We then took the set of all the genes tested based on expression alone, split them randomly into groups of the same size as the ChromGene annotations, and determined significant gene set enrichments as above. We repeated this process 100 times and took the mean number of significant enrichments. We repeated the process for each cell type and represented each one as a point in the scatter plot.

### pLI score analysis

We obtained pLI scores for each gene from gnomAD [25, 53] using the “exac_pLI” column. We then correlated the expression value of genes across 56 cell types [1] with their corresponding pLI scores using spearman correlation.

To calculate the proportion of high-pLI genes (Fig. 8) and mean of pLI scores (Additional File 1: Fig. S10) as a function of expression for a given cell type, we took each gene’s expression, added a pseudocount of 0.1 RPKM to it, and took the log_10_ of the result (gray curves). We then looked at 30 equally sized bins of this transformed expression value, from −1 to 2, corresponding to 0 and ≈100 RPKM, respectively. For each bin, we took all genes falling into it, and calculated both the proportion of these genes with pLI ≥ 0.9 and the mean pLI of these genes for the two figures, respectively. We plotted the curves by linearly interpolating between bins. We then repeated the process for all cell types simultaneously (black dotted curve).

To show that the difference in percentage of high-pLI genes among ChromGene annotations (Fig. 8) is not simply due to differences in gene length, for each cell type, we first took all genes assigned to a ChromGene annotation, then split them into bins based on the log_10_ of their length, where the log_10_ length ranged from 3 (1000 bp) to 6 (1Mbp), and each bin spanned 0.2 log_10_ length, for a total of 15 bins. We used the range of 1 kb–1 Mb as there are very few genes outside this range (394 genes with length < 1 kb, 63 genes with length > 1 MB, which we discarded for this analysis). For each length bin and ChromGene annotation, we counted the number of genes in the bin with high pLI score (≥ 0.9) across all cell types. We then normalized the resulting values to yield the proportion of genes with high pLI score for each ChromGene annotation and length bin (Additional File 1: Fig. S9). To explicitly compare the differences in pLI scores across annotations controlling for gene length, we first binned all gene lengths by their log_10_ length as above, normalized so the distribution summed to 1, and used this as the reference gene length density. Then, we took the proportions of genes with high pLI scores per ChromGene annotation across the bins, as described above, and multiplied them by their respective reference gene length density. Finally, for each ChromGene annotation, we summed the resulting values to obtain a gene length-normalized proportion of high-pLI score genes (Additional File 2: Table S1).

### Supplementary Information

**Additional file 1: Fig. S1.** Model reproducibility and state similarity as a function of hyperparameters. **Fig. S2.** ChromGene state transitions. **Fig. S3.** Median Expression for each ChromGene assignment, separated by cell type. **Fig. S4.** Mutual information of annotation method and gene length. **Fig. S5.** ChromGene confusion matrix and contingency table. **Fig. S6.** Log_2_ enrichments of ChromHMM states for each ChromGene assignment. **Fig. S7.** Comparison of gene expression as a function of ChromGene annotations across pairs of cell. **Fig. S8.** Median GO term enrichment across all cell types. **Fig. S9.** Proportion of high-pLI genes per ChromGene annotation, conditioned on gene length types. **Fig. S10.** Mean pLI vs mean expression. **Table S2.** Performance of ChromGene compared to baseline methods at predicting expression.

**Additional file 2:** **Table S1.** Description of ChromGene annotation enrichments, state emissions and enrichments, and transition probabilities. **Annotation enrichments tab** – The columns on this tab after the annotation colors and numbers are as follows: Mnemonic: short identifying name of ChromGene annotation. Description: short description of ChromGene annotation. Overall percentage: percentage of gene-cell type combinations assigned to annotation. Median expression: median expression (RPKM) of genes assigned to annotation across 56 cell types with expression [1]. Median length (kb): median length (kb) of genes assigned to annotation across all cell types, not including flanking regions. Percentage of high-pLI genes (pLI ≥ 0.9): percentage of genes across cell types that have a pLI score ≥ 0.9. Percentage of high-pLI genes (pLI ≥ 0.9), conditioned on gene length: percentage of genes across cell types that have a pLI score ≥ 0.9 in a gene length matched distribution (“Methods”). Contingency table diagonal / confusion matrix diagonal: percentage consistency of ChromGene assignments across non-replicate cell types divided by percentage consistency of assignments across replicate cell types. Cell type specificity: 1 - (contingency table diagonal / confusion matrix diagonal), a metric of cell type specificity. # Housekeeping gene: gene annotated as housekeeping [39]. Housekeeping gene percentage: percentage of gene-cell type combinations annotated as a housekeeping gene. Housekeeping gene enrichment: fold enrichment of housekeeping genes compared to overall percentage. Housekeeping gene log_2_ enrichment: log_2_ fold enrichment of housekeeping genes. Housekeeping gene enrichment median enrichment *p*-value: median *p*-value of housekeeping gene enrichment across cell types. # Constitutively unexpressed gene: gene that has RPKM < 1 across 56 cell types with expression [1]. Constitutively unexpressed gene percentage: percentage of gene-cell type combinations annotated as constitutively unexpressed. Constitutively unexpressed gene enrichment: fold enrichment of constitutively unexpressed genes compared to overall percentage. Constitutively unexpressed gene log_2_ enrichment: log_2_ fold enrichment of constitutively unexpressed genes. Constitutively unexpressed gene median enrichment *p*-value: median *p*-value of constitutively unexpressed gene enrichment across cell types. # Constitutively expressed gene: gene that has RPKM> 1 across 56 cell types with expression [1]. Constitutively expressed gene percentage: percentage of gene-cell type combinations annotated as constitutively expressed. Constitutively expressed gene enrichment: fold enrichment of constitutively expressed genes compared to overall percentage. Constitutively expressed gene log_2_ enrichment: log_2_ fold enrichment of constitutively expressed genes. *Constitutively expressed gene median* enrichment *p*-value: median *p*-value of constitutively expressed gene enrichment across cell types. # Olfactory gene: gene annotated as olfactory [34]. Olfactory gene percentage: percentage of gene / cell type combinations annotated as olfactory. Olfactory gene enrichment: fold enrichment of olfactory genes compared to overall percentage. Olfactory gene log_2_ enrichment: log_2_ fold enrichment of olfactory genes. Olfactory gene median enrichment *p*-value: median *p*-value of olfactory gene enrichment across cell types. # ZNF gene: gene starts with "ZNF". ZNF gene percentage: percentage of gene / cell type combinations annotated as ZNF. ZNF gene enrichment: fold enrichment of ZNF genes compared to overall percentage. ZNF gene log_2_ enrichment: log_2_ fold enrichment of ZNF genes. ZNF gene median enrichment *p*-value: median *p*-value of ZNF gene enrichment across cell types. Cancer gene sets enriched (adj *p* < 0.01): the number of cancer gene sets enriched across all cell types for given annotation. Cancer gene sets enriched percentage: the percentage of cancer gene sets enriched across all cell types for given annotation. BP GO terms enriched (adj *p* < 0.01): the number of 'Biological Process' GO term gene sets enriched across all cell types for given annotation. BP GO terms enriched percentage: the percentage of 'Biological Process' GO term gene sets enriched across all cell types for given annotation. Color (hex): hex color for ChromGene annotation. Matplotlib color name: color used in matplotlib for ChromGene annotation [54]. **State emissions and enrichments tab** – The first column gives a color and number for each annotation. The second column gives the annotation mnemonic. The third column gives a number to each individual state of the mixture component. The next 12 columns give the emission probabilities for each epigenomic mark as indicated. The next two columns give the maximum and minimum emission probabilities represented as percentages. The next column gives the enrichment of the individual states for annotated TSS. Individual states within each mixture are ordered in decreasing value of this enrichment. The next column gives the initial probability of starting in the state overall. The last column gives the initial probability of the state given the component. **Transition probabilities tab** – This tab shows the transition probability, which indicates the probability, when in the state of the row, of transitioning to the state of the column. Probabilities are shown for individual states of the model, which are ordered and colored based on the component to which they belong, as indicated.

**Additional file 3.** ChromGene assignments. An Excel spreadsheet containing the ChromGene assignments for the 127 cell types. These assignments are reported in four tabs, two based on the hg19 assembly and gene annotation (ENSEMBL v65/GENCODE v10) and two based on an hg38-based gene annotation (GENCODE v41) lifted over to hg19. For each assembly, we provide one tab using the Roadmap Epigenomics Consortium “Standardized Epigenome names” for designating the cell type, and another tab using the Epigenome IDs (EIDs) [1]. Each row after the header row corresponds to one gene. The first five columns from left to right are the chromosome of the gene, the left-most coordinate of the gene, the right-most coordinate of the gene, the gene symbol, and strand of the gene. The remaining 127 columns correspond to different cell types, and the entries to ChromGene assignments.

**Additional file 4.** Review history.

## Data Availability

The ChromGene annotations generated in this study and the ChromGene software are available under the MIT license in the ErnstLab ChromGene GitHub repository [30] and Zenodo [31]. We used ChromHMM version 1.18 to train the ChromGene model. No other scripts or software were used. ChromGene assignments are also available in Additional file 3. Data used to generate input for ChromGene is also publicly available [1, 27, 50].

## References

[1] Kundaje A, Meuleman W, Ernst J, Bilenky M, Yen A, Heravi-Moussavi A (2015). Integrative analysis of 111 reference human epigenomes. Nature.

[2] Ernst J, Kheradpour P, Mikkelsen TS, Shoresh N, Ward LD, Epstein CB (2011). Mapping and analysis of chromatin state dynamics in nine human cell types. Nature.

[3] Barski A, Cuddapah S, Cui K, Roh T-Y, Schones DE, Wang Z (2007). High-resolution profiling of histone methylations in the human genome. Cell.

[4] ENCODE Project Consortium (2012). An integrated encyclopedia of DNA elements in the human genome. Nature.

[5] Boyle AP, Song L, Lee B-K, London D, Keefe D, Birney E (2011). High-resolution genome-wide in vivo footprinting of diverse transcription factors in human cells. Genome Res.

[6] Buenrostro JD, Giresi PG, Zaba LC, Chang HY, Greenleaf WJ (2013). Transposition of native chromatin for fast and sensitive epigenomic profiling of open chromatin, DNA-binding proteins and nucleosome position. Nat Methods.

[7] Stunnenberg HG, Hirst M, International Human Epigenome Consortium (2016). The international human epigenome consortium: a blueprint for scientific collaboration and discovery. Cell..

[8] Ernst J, Kellis M (2010). Discovery and characterization of chromatin states for systematic annotation of the human genome. Nat Biotechnol.

[9] Ernst J, Kellis M (2012). ChromHMM: automating chromatin state discovery and characterization. Nat Methods.

[10] Hoffman MM, Buske OJ, Wang J, Weng Z, Bilmes JA, Noble WS (2012). Unsupervised pattern discovery in human chromatin structure through genomic segmentation. Nat Methods.

[11] Claussnitzer M, Dankel SN, Kim K-H, Quon G, Meuleman W, Haugen C (2015). FTO obesity variant circuitry and adipocyte browning in humans. N Engl J Med.

[12] Libbrecht MW, Chan RCW, Hoffman MM (2021). Segmentation and genome annotation algorithms for identifying chromatin state and other genomic patterns. PLOS Comput Biol.

[13] Wang Z, Gerstein M, Snyder M (2009). RNA-Seq: a revolutionary tool for transcriptomics. Nat Rev Genet.

[14] Mortazavi A, Williams BA, McCue K, Schaeffer L, Wold B (2008). Mapping and quantifying mammalian transcriptomes by RNA-Seq. Nat Methods.

[15] Eisen MB, Spellman PT, Brown PO, Botstein D (1998). Cluster analysis and display of genome-wide expression patterns. Proc Natl Acad Sci.

[16] Frankish A, Diekhans M, Ferreira A-M, Johnson R, Jungreis I, Loveland J (2019). GENCODE reference annotation for the human and mouse genomes. Nucleic Acids Res.

[17] Su D, Wang X, Campbell MR, Song L, Safi A, Crawford GE (2015). Interactions of chromatin context, binding site sequence content, and sequence evolution in stress-induced p53 occupancy and transactivation. PLOS Genet.

[18] Zhu W, Hu B, Becker C, Doğan ES, Berendzen KW, Weigel D (2017). Altered chromatin compaction and histone methylation drive non-additive gene expression in an interspecific Arabidopsis hybrid. Genome Biol.

[19] Kharchenko PV, Alekseyenko AA, Schwartz YB, Minoda A, Riddle NC, Ernst J (2011). Comprehensive analysis of the chromatin landscape in Drosophila melanogaster. Nature.

[20] Sahu A, Li N, Dunkel I, Chung H-R (2020). EPIGENE: genome-wide transcription unit annotation using a multivariate probabilistic model of histone modifications. Epigenetics Chromatin.

[21] Marco E, Meuleman W, Huang J, Glass K, Pinello L, Wang J (2017). Multi-scale chromatin state annotation using a hierarchical hidden Markov model. Nat Commun.

[22] Jaschek R, Tanay A (2009). Spatial clustering of multivariate genomic and epigenomic information.

[23] Larson JL, Huttenhower C, Quackenbush J, Yuan G-C (2013). A tiered hidden Markov model characterizes multi-scale chromatin states. Genomics.

[24] Ge X, Zhang H, Xie L, Li WV, Kwon SB, Li JJ (2019). EpiAlign: an alignment-based bioinformatic tool for comparing chromatin state sequences. Nucleic Acids Res.

[25] Lek M, Karczewski KJ, Minikel EV, Samocha KE, Banks E, Fennell T (2016). Analysis of protein-coding genetic variation in 60,706 humans. Nature.

[26] Robinson JT, Thorvaldsdóttir H, Winckler W, Guttman M, Lander ES, Getz G (2011). Integrative genomics viewer. Nat Biotechnol.

[27] Ernst J, Kellis M (2015). Large-scale imputation of epigenomic datasets for systematic annotation of diverse human tissues. Nat Biotechnol.

[28] Ernst J, Kellis M (2017). Chromatin-state discovery and genome annotation with ChromHMM. Nat Protoc.

[29] Heintzman ND, Stuart RK, Hon G, Fu Y, Ching CW, Hawkins RD (2007). Distinct and predictive chromatin signatures of transcriptional promoters and enhancers in the human genome. Nat Genet.

[30] Jaroszewicz A, Ernst J. ChromGene github site. https://github.com/ernstlab/ChromGene/. Accessed 28 Mar 2023.

[31] Jaroszewicz A, Ernst J. ChromGene: gene-based modeling of epigenomic data. Zenodo. 10.5281/zenodo.8303613. 10.1186/s13059-023-03041-5 PMC10486095 37679846

[32] Lesch BJ, Page DC (2014). Poised chromatin in the mammalian germ line. Dev Camb Engl.

[33] Heintzman ND, Hon GC, Hawkins RD, Kheradpour P, Stark A, Harp LF (2009). Histone modifications at human enhancers reflect global cell-type-specific gene expression. Nature.

[34] Barnes IHA, Ibarra-Soria X, Fitzgerald S, Gonzalez JM, Davidson C, Hardy MP (2020). Expert curation of the human and mouse olfactory receptor gene repertoires identifies conserved coding regions split across two exons. BMC Genomics.

[35] Ashburner M, Ball CA, Blake JA, Botstein D, Butler H, Cherry JM (2000). Gene Ontology: tool for the unification of biology. Nat Genet.

[36] Kuleshov MV, Jones MR, Rouillard AD, Fernandez NF, Duan Q, Wang Z (2016). Enrichr: a comprehensive gene set enrichment analysis web server 2016 update. Nucleic Acids Res.

[37] Barretina J, Caponigro G, Stransky N, Venkatesan K, Margolin AA, Kim S (2012). The Cancer Cell Line Encyclopedia enables predictive modelling of anticancer drug sensitivity. Nature.

[38] Chen EY, Tan CM, Kou Y, Duan Q, Wang Z, Meirelles GV (2013). Enrichr: interactive and collaborative HTML5 gene list enrichment analysis tool. BMC Bioinformatics.

[39] Eisenberg E, Levanon EY (2013). Human housekeeping genes, revisited. Trends Genet.

[40] Bernstein BE, Mikkelsen TS, Xie X, Kamal M, Huebert DJ, Cuff J (2006). A bivalent chromatin structure marks key developmental genes in embryonic stem cells. Cell.

[41] Botía JA, Vandrovcova J, Forabosco P, Guelfi S, D’Sa K, Hardy J (2017). An additional k-means clustering step improves the biological features of WGCNA gene co-expression networks. BMC Syst Biol.

[42] Costa IG, Roepcke S, Hafemeister C, Schliep A (2008). Inferring differentiation pathways from gene expression. Bioinformatics.

[43] Chaffer CL, Marjanovic ND, Lee T, Bell G, Kleer CG, Reinhardt F (2013). Poised chromatin at the ZEB1 promoter enables breast cancer cell plasticity and enhances tumorigenicity. Cell.

[44] Bernhart SH, Kretzmer H, Holdt LM, Jühling F, Ammerpohl O, Bergmann AK (2016). Changes of bivalent chromatin coincide with increased expression of developmental genes in cancer. Sci Rep.

[45] Vu H, Ernst J (2022). Universal annotation of the human genome through integration of over a thousand epigenomic datasets. Genome Biol.

[46] Rabiner LR (1989). A tutorial on hidden Markov models and selected applications in speech recognition. Proc IEEE.

[47] Durbin R, Eddy SR, Krogh A, Mitchison G (1998). Biological sequence analysis: probabilistic models of proteins and nucleic acids.

[48] Murphy KP. Hidden semi-Markov models (HSMMs). 2002. https://www.cs.ubc.ca/~murphyk/Papers/segment.pdf. Accessed 28 Mar 2023.

[49] Frankish A, Diekhans M, Ferreira A-M, Johnson R, Jungreis I, Loveland J, et al. GENCODE V41 Annotation. Nucleic Acids Research. https://egg2.wustl.edu/roadmap/data/byDataType/rna/expression/Ensembl_v65.Gencode_v10.ENSG.gene_info. Accessed 28 Mar 2023.

[50] Roadmap Epigenomics Consortium. Roadmap Epigenomics Consortium ChromHMM Imputed Data. https://egg2.wustl.edu/roadmap/data/byFileType/chromhmmSegmentations/binaryChmmInput/imputed12marks/binaryData/. Accessed 28 Mar 2023.

[51] Roadmap Epigenomics Consortium. Roadmap Epigenomics Consortium Gene Expression Data. https://egg2.wustl.edu/roadmap/data/byDataType/rna/expression/57epigenomes.RPKM.pc.gz. Accessed 28 Mar 2023.

[52] Frankish A, Diekhans M, Ferreira A-M, Johnson R, Jungreis I, Loveland J, et al. GENCODE V41 Annotation hg19 to hg38 Liftover. Nucleic Acids Research. https://ftp.ebi.ac.uk/pub/databases/gencode/Gencode_human/release_41/GRCh37_mapping/gencode.v41lift37.basic.annotation.gtf.gz. Accessed 28 Mar 2023.

[53] Lek M, Karczewski KJ, Minikel EV, Samocha KE, Banks E, Fennell T, et al. gnomAD Browser pLI Scores. https://storage.googleapis.com/gcp-public-data--gnomad/release/2.1.1/constraint/gnomad.v2.1.1.lof_metrics.by_gene.txt.bgz. Accessed 28 Mar 2023.

[54] Munroe R. XKCD Colors. https://xkcd.com/color/rgb/. Accessed 28 Mar 2023.

