# Supplementary material for: ChromGene: gene-based modeling of epigenomic data
Source: Genome Biol. 2023 Sep 7;24:203. doi: 10.1186/s13059-023-03041-5 (PMC10486095; doi:10.1186/s13059-023-03041-5)
Supplement: Supplementary file 1 — Additional file 1: Fig. S1. Model reproducibility and state similarity as a function of hyperparameters. Fig. S2. ChromGene state transitions. Fig. S3. Median Expression for each ChromGene assignment, separated by cell type. Fig. S4. Mutual information of annotation method and gene length. Fig. S5. ChromGene confusion matrix and contingency table. Fig. S6. Log2 enrichments of ChromHMM states for each ChromGene assignment. Fig. S7. Comparison of gene expression as a function of ChromGene annotations across pairs of cell. Fig. S8. Median GO term enrichment across all cell types. Fig. S9. Proportion of high-pLI genes per ChromGene annotation, conditioned on gene length types. Fig. S10. Mean pLI vs mean expression. Table S2. Performance of ChromGene compared to baseline methods at predicting expression. [file 13059_2023_3041_MOESM1_ESM.pdf]

ChromGene: gene-based modeling of epigenomic data

Artur Jaroszewicz and Jason Ernst

Figures S1-10

Table S2

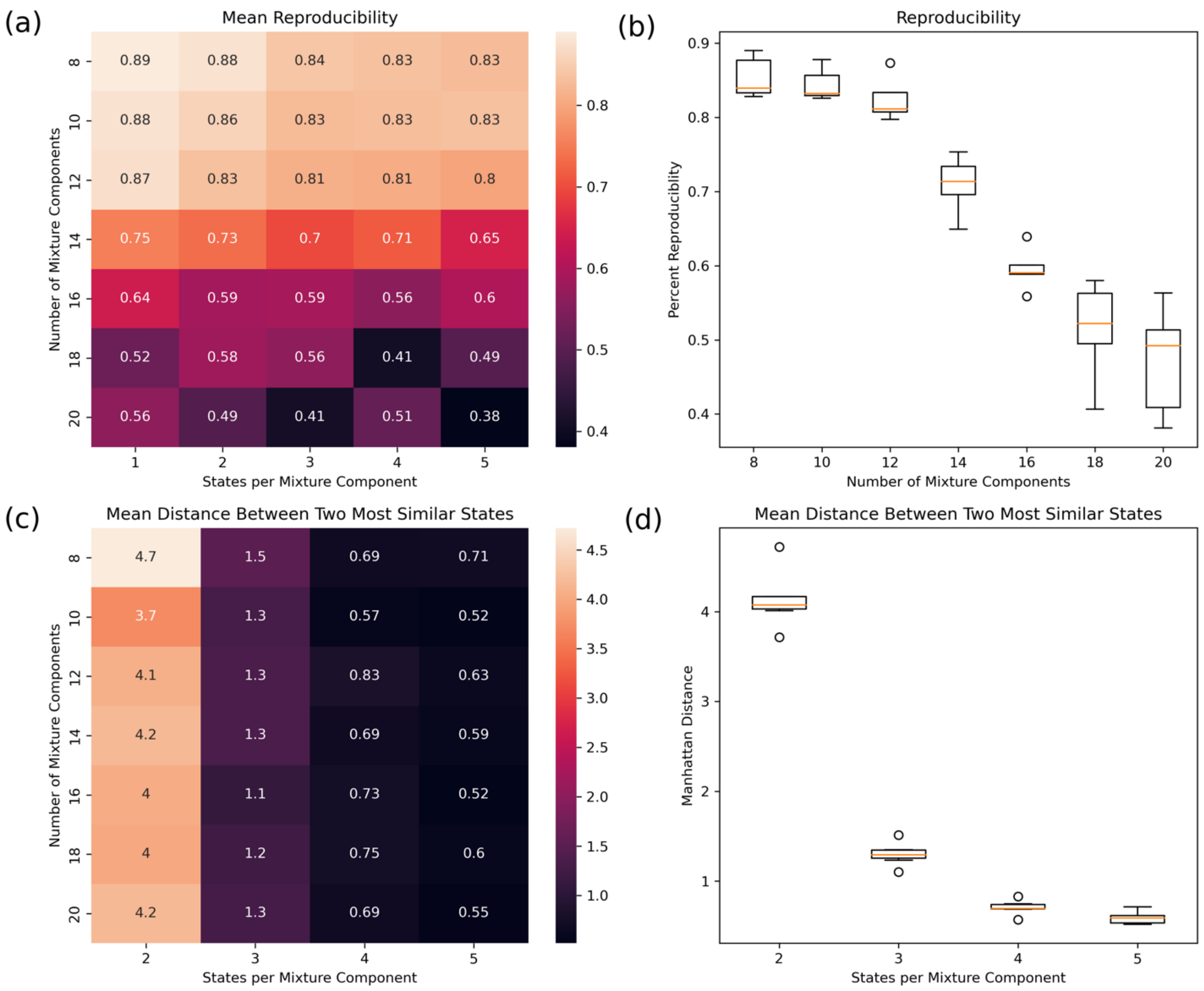

Fig. S1: Model reproducibility and state similarity as a function of hyperparameters

(a) Reproducibility of ChromGene assignments as a function of *M* (the number of mixture components) and *S* (the number of states per component) (**Methods**). Reproducibility is more sensitive to the choice of *M* than *S*.

(b) Reproducibility as a function of *M*, represented as a boxplot over values of *S*.

(c) Manhattan distance between two closest states within a mixture, for values of *M*=8 to 20, and *S*=2 to 5. Distance is more sensitive to the choice of *S* than *M*.

(d) Manhattan distance between two closest states within a mixture as a function of *S*, represented as a boxplot over values of *M*.

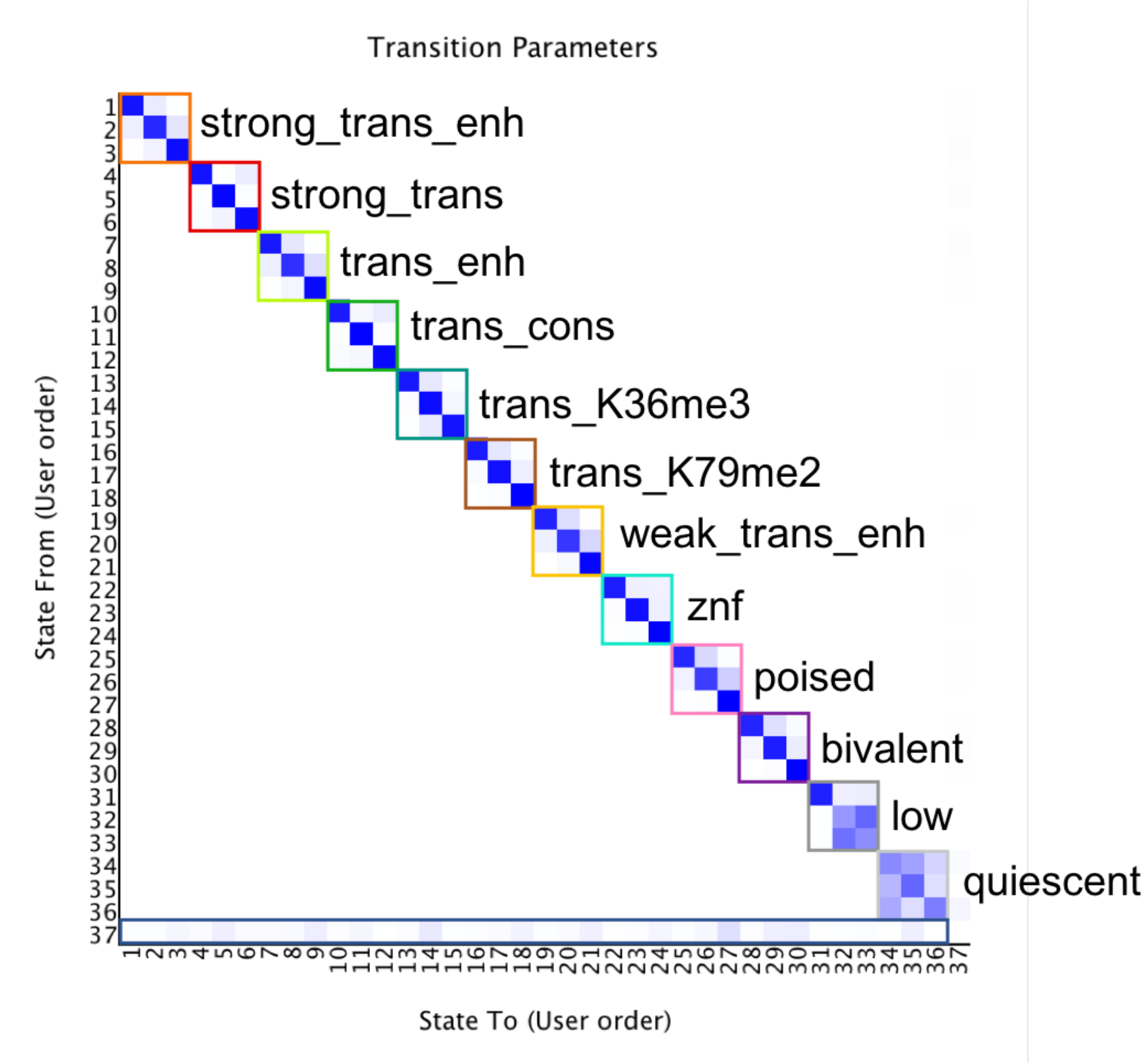

Fig. S2: ChromGene state transitions

Transition parameters between states within each component are marked in 3x3 squares. The bottom bar represents the initial probability of each state, independent of component. The component prior probabilities are the sums of the initial probabilities for their corresponding states. Transition probabilities are reported in (**Additional File 2: Table S1**).

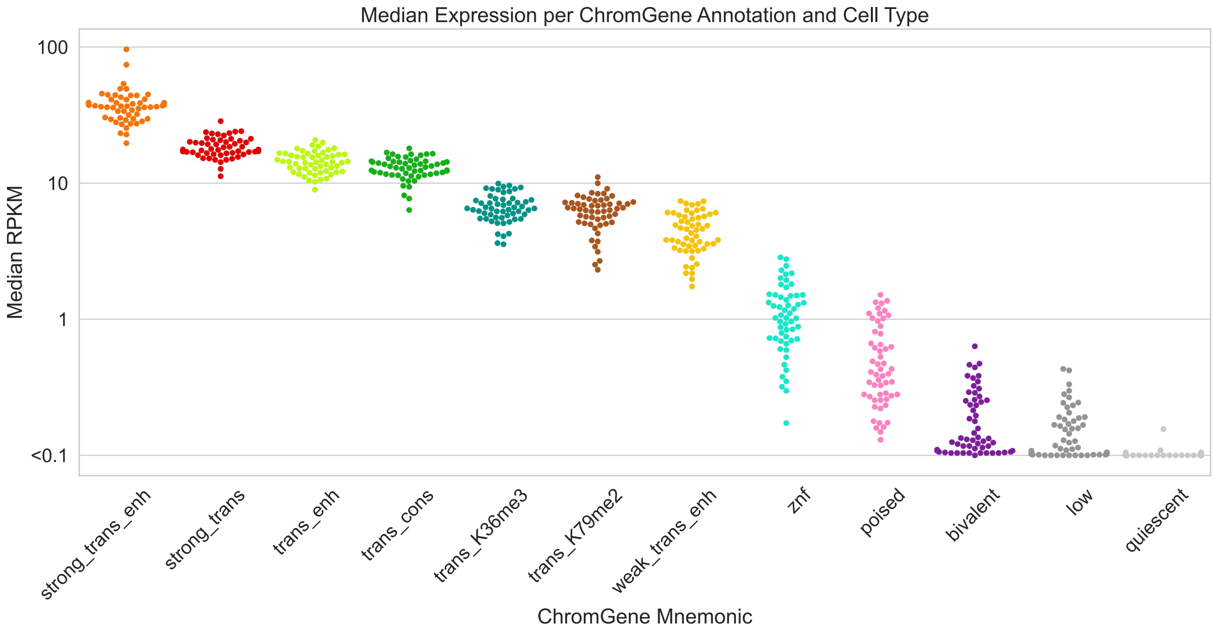

Fig. S3: Median Expression for each ChromGene assignment, separated by cell type

Distribution of median expression (RPKM) per ChromGene assignment over 56 cell types with expression data available, where each point represents the median expression of all genes in a single cell type assigned to a given ChromGene annotation. Most points for the 'quiescent' annotation had values < 0.1 RPKM and were not plotted due to space.

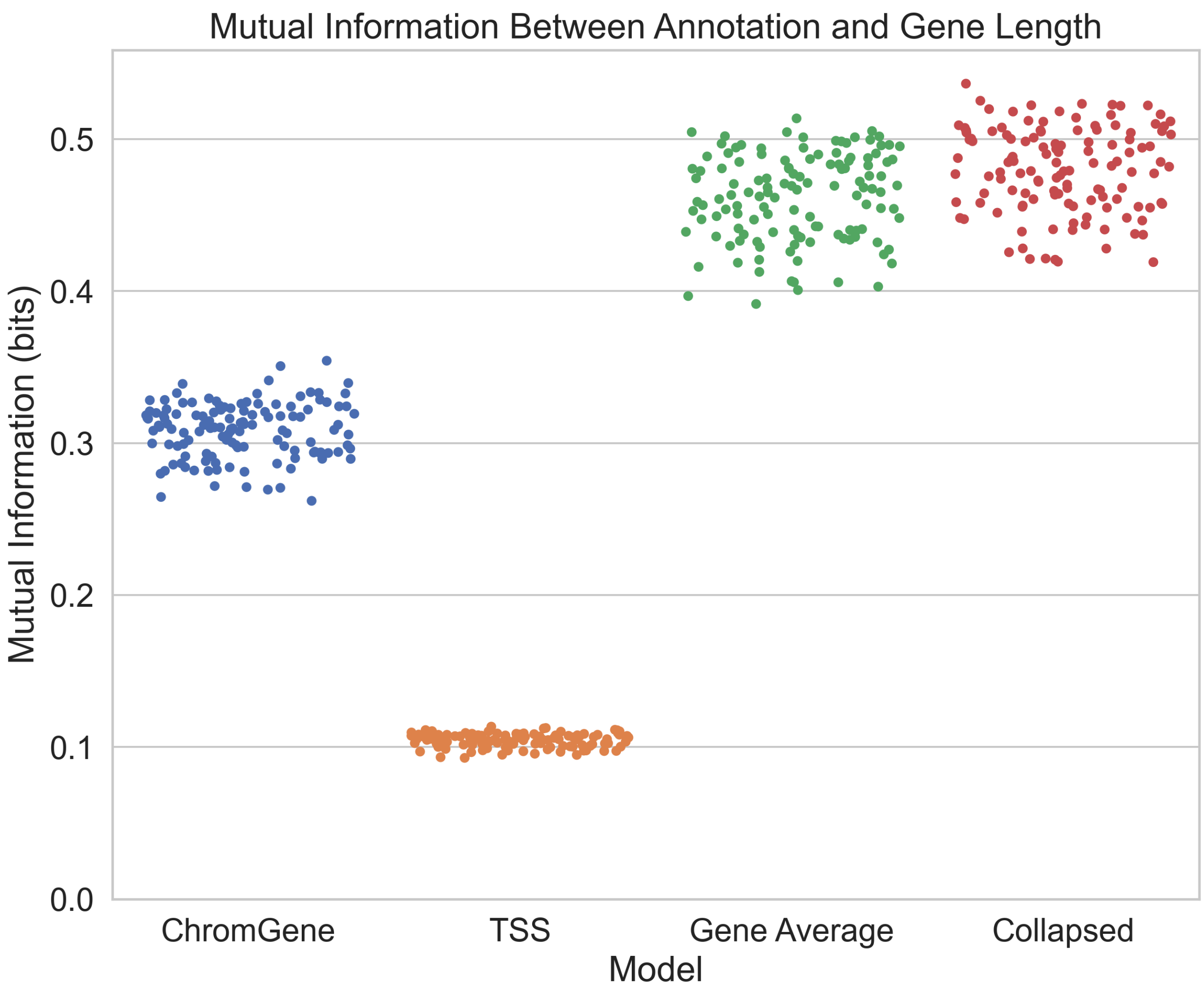

Fig. S4: Mutual information of annotation method and gene length

The mutual information of annotation method and gene lengths, where each point represents a cell type. Horizontal axis: ChromGene and three baselines: TSS, Gene Average, and Collapsed Models. ChromGene assignments have less information shared with the gene length than baseline methods that incorporate information from the whole gene (Gene Average, Collapsed) ($p < 10^{-30}$, paired binomial test, **Methods**), indicating that ChromGene annotations are less likely to directly reflect information about gene length. Annotations from the TSS model have the lowest mutual information with gene length, as expected, given it only uses information at the TSS, defined here as a single 200bp bin.

(a)

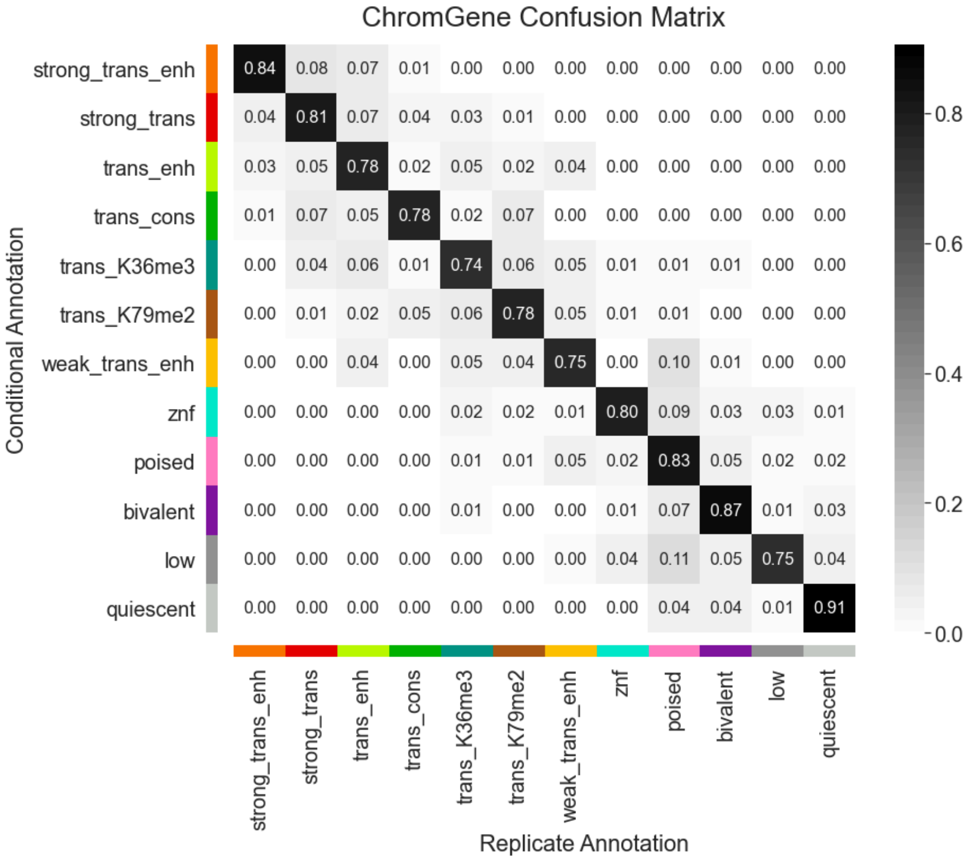
ChromGene Confusion Matrix
Conditional Annotation
Replicate Annotation
strong_trans_enh 0.84 0.08 0.07 0.01 0.00 0.00 0.00 0.00 0.00 0.00 0.00 0.00
strong_trans 0.04 0.81 0.07 0.04 0.03 0.01 0.00 0.00 0.00 0.00 0.00 0.00
trans_enh 0.03 0.05 0.78 0.02 0.05 0.02 0.04 0.00 0.00 0.00 0.00 0.00
trans_cons 0.01 0.07 0.05 0.78 0.02 0.07 0.00 0.00 0.00 0.00 0.00 0.00
trans_K36me3 0.00 0.04 0.06 0.01 0.74 0.06 0.05 0.01 0.01 0.01 0.00 0.00
trans_K79me2 0.00 0.01 0.02 0.05 0.06 0.78 0.05 0.01 0.01 0.00 0.00 0.00
weak_trans_enh 0.00 0.00 0.04 0.00 0.05 0.04 0.75 0.00 0.10 0.01 0.00 0.00
znf 0.00 0.00 0.00 0.00 0.02 0.02 0.01 0.80 0.09 0.03 0.03 0.01
poised 0.00 0.00 0.00 0.00 0.01 0.01 0.05 0.02 0.83 0.05 0.02 0.02
bivalent 0.00 0.00 0.00 0.00 0.01 0.00 0.00 0.01 0.07 0.87 0.01 0.03
low 0.00 0.00 0.00 0.00 0.00 0.00 0.00 0.04 0.11 0.05 0.75 0.04
quiescent 0.00 0.00 0.00 0.00 0.00 0.00 0.00 0.00 0.04 0.04 0.01 0.91
strong_trans_enh strong_trans trans_enh trans_cons trans_K36me3 trans_K79me2 weak_trans_enh znf poised bivalent low quiescent
0.8
0.6
0.4
0.2
0.0

(b)

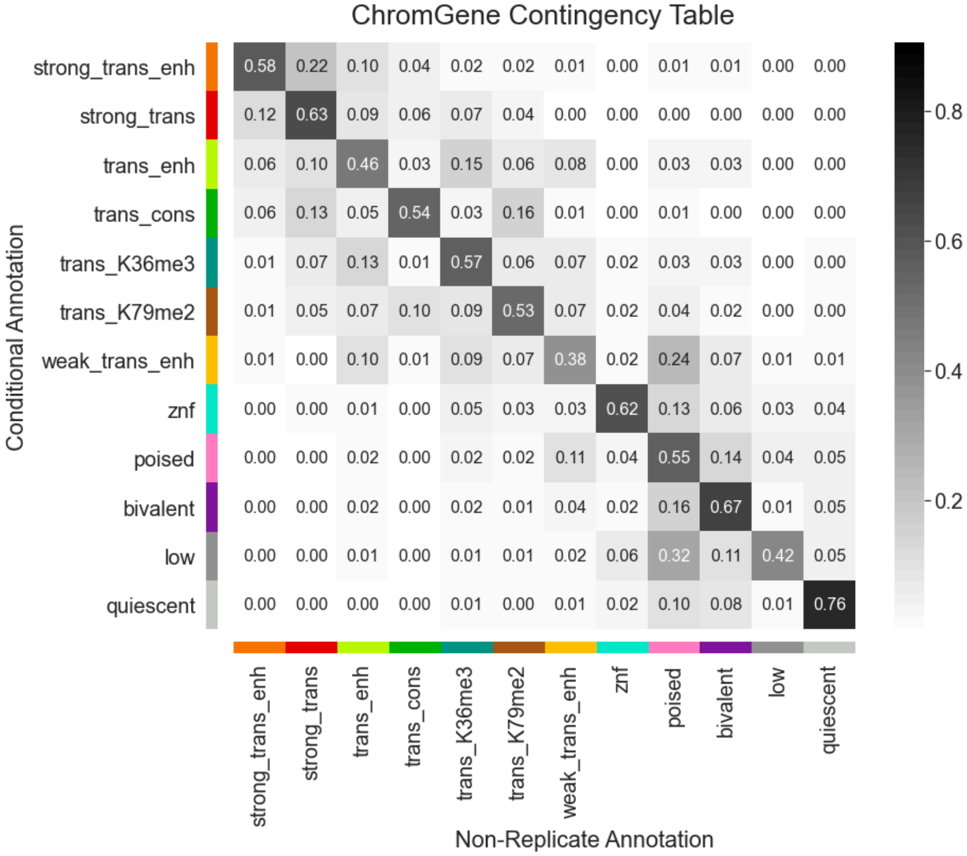
ChromGene Contingency Table
Conditional Annotation
Non-Replicate Annotation
strong_trans_enh 0.58 0.22 0.10 0.04 0.02 0.02 0.01 0.00 0.01 0.01 0.00 0.00
strong_trans 0.12 0.63 0.09 0.06 0.07 0.04 0.00 0.00 0.00 0.00 0.00 0.00
trans_enh 0.06 0.10 0.46 0.03 0.15 0.06 0.08 0.00 0.03 0.03 0.00 0.00
trans_cons 0.06 0.13 0.05 0.54 0.03 0.16 0.01 0.00 0.01 0.00 0.00 0.00
trans_K36me3 0.01 0.07 0.13 0.01 0.57 0.06 0.07 0.02 0.03 0.03 0.00 0.00
trans_K79me2 0.01 0.05 0.07 0.10 0.09 0.53 0.07 0.02 0.04 0.02 0.00 0.00
weak_trans_enh 0.01 0.00 0.10 0.01 0.09 0.07 0.38 0.02 0.24 0.07 0.01 0.01
znf 0.00 0.00 0.01 0.00 0.05 0.03 0.03 0.62 0.13 0.06 0.03 0.04
poised 0.00 0.00 0.02 0.00 0.02 0.02 0.11 0.04 0.55 0.14 0.04 0.05
bivalent 0.00 0.00 0.02 0.00 0.02 0.01 0.04 0.02 0.16 0.67 0.01 0.05
low 0.00 0.00 0.01 0.00 0.01 0.01 0.02 0.06 0.32 0.11 0.42 0.05
quiescent 0.00 0.00 0.00 0.00 0.01 0.00 0.01 0.02 0.10 0.08 0.01 0.76
strong_trans_enh strong_trans trans_enh trans_cons trans_K36me3 trans_K79me2 weak_trans_enh znf poised bivalent low quiescent
0.8
0.6
0.4
0.2

Fig. S5: ChromGene confusion matrix and contingency table

(a) Confusion matrix for ChromGene annotations between pairs of cell types that can be treated as biological replicates.

(b) Contingency table of probabilities between pairs of cell types that are not biological replicates (**Methods**). In both the confusion and contingency matrices, rows correspond to the annotation being conditioned on and sum to unity probability.

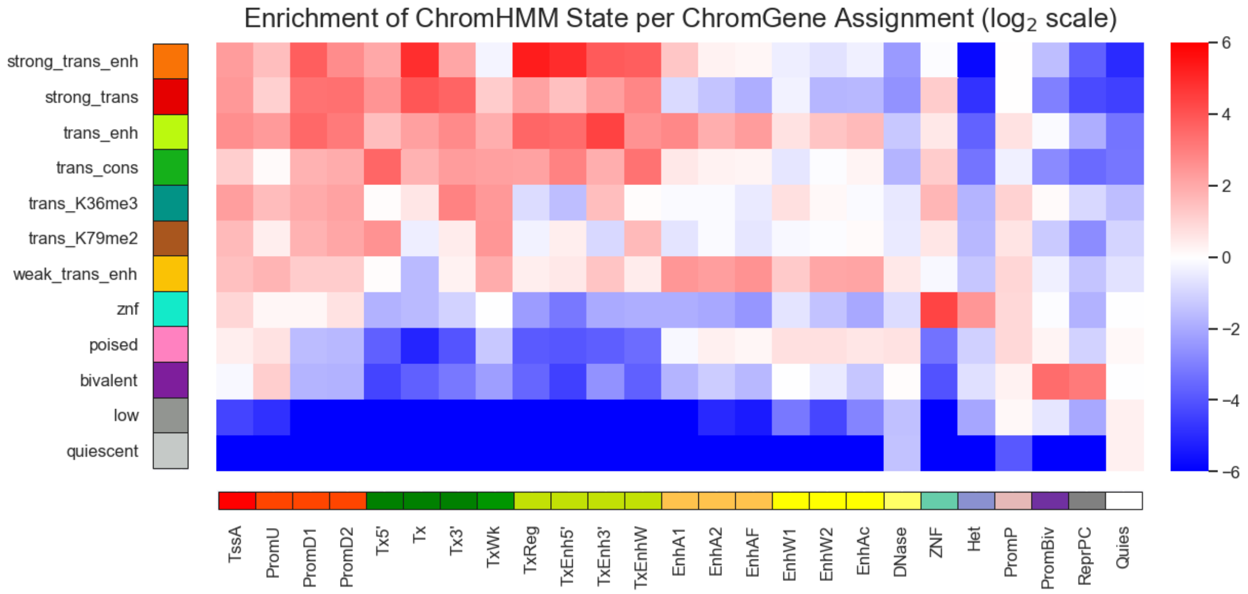

Fig. S6: Log$_2$ enrichments of ChromHMM states for each ChromGene assignment

Heatmap where the rows correspond to different ChromGene assignments and the columns to different cell type-specific chromatin states from a previous ChromHMM 25-state model based on the same imputed data [28]. Heatmap values indicate log$_2$ enrichments for the matched chromatin state annotation in the ChromGene annotation. We set the minimum log$_2$ enrichment to -6 to prevent washing out of the color scale due to highly negative values.

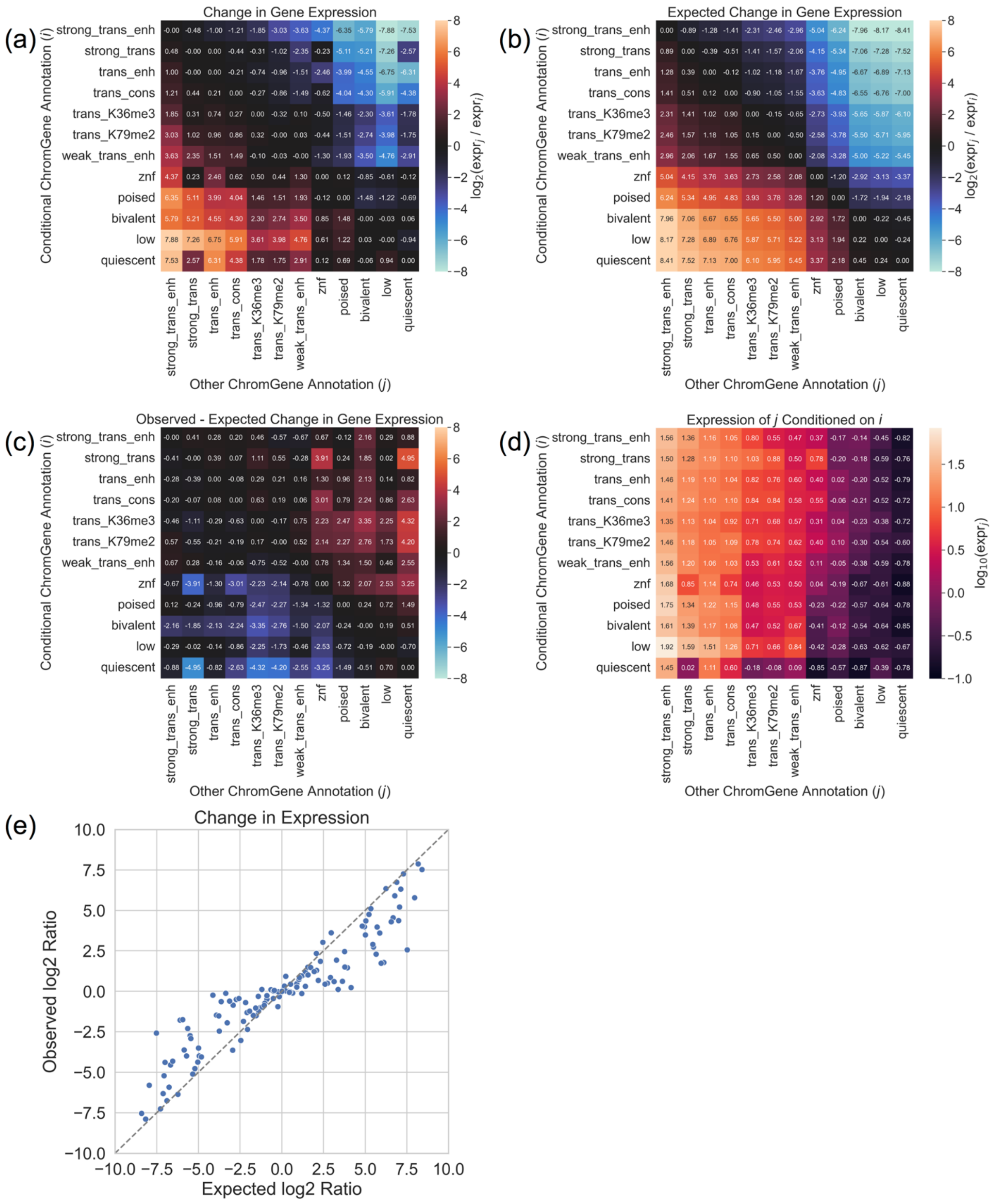

Fig. S7: Comparison of gene expression as a function of ChromGene annotations across pairs of cell types

(a) A heatmap showing $\log_2$ ratio in expression [$\log_2((RPKM_j + 0.1) / (RPKM_i + 0.1))$] for genes in ChromGene annotation *i* in the conditional cell type (rows) and ChromGene annotation *j* in the "other" cell type (columns), averaged over combinations of genes and cell type pairs. Positive values correspond to a higher expression of the gene in the latter cell type and ChromGene annotation. Certain pairs of ChromGene annotations had less variation in expression than expected based on individual expression distributions ('znf'-'strong_trans', 'quiescent'-'strong_trans', 'quiescent'-'trans_K36me3', 'quiescent'-'trans_K79me2'). For example, if a gene was assigned to 'znf' in cell type *i* and 'strong_trans' in cell type *j*, then the expression only increased on average by 1.2 fold ($\log_2$ fold change of 0.23), as compared to an expected change of 19.4 fold ($\log_2$ fold change of 4.27) as determined by dividing the median expressions of those annotations across all cell types. These results are unlikely to be artifacts of low counts, as we found 11094, 570, 25410, and 16803 such changes in ChromGene assignment across all pairs of cell types, respectively. In this scenario, the mean expression of genes in the 'strong_trans' annotation for cell type *j* (7.08 RPKM) was lower than the overall mean expression of 'strong_trans' (18.24 RPKM), while genes in the 'znf' annotation were higher (6.03 RPKM) than overall (0.94 RPKM) (**Additional File 1: Fig. S7d**).

(b) The expected $\log_2$ ratio in expression [$\log_2(RPKM_j / RPKM_i)$], represented as in (**Additional File 1: Fig. S7a**). Values were calculated based on median expression for each ChromGene annotation (**Fig 3**, **Additional File 2: Table S1**, **Methods**).

(c) The observed minus expected $\log_2$ ratio in expression, taken by subtracting the matrix represented in (**Additional File 1: Fig. S7b**) from that in (**Additional File 1: Fig. S7a**).

(d) A heatmap showing the mean expression [$\log_{10}(RPKM)$] for genes in the ChromGene annotation *j* in the "other" cell type (columns) conditioned on the same gene being assigned to ChromGene annotation *i* in another cell type (rows).

(e) A scatterplot showing the observed minus expected $\log_2$ ratio in expression between pairs of ChromGene annotations. Each point is a pair of ChromGene annotations.

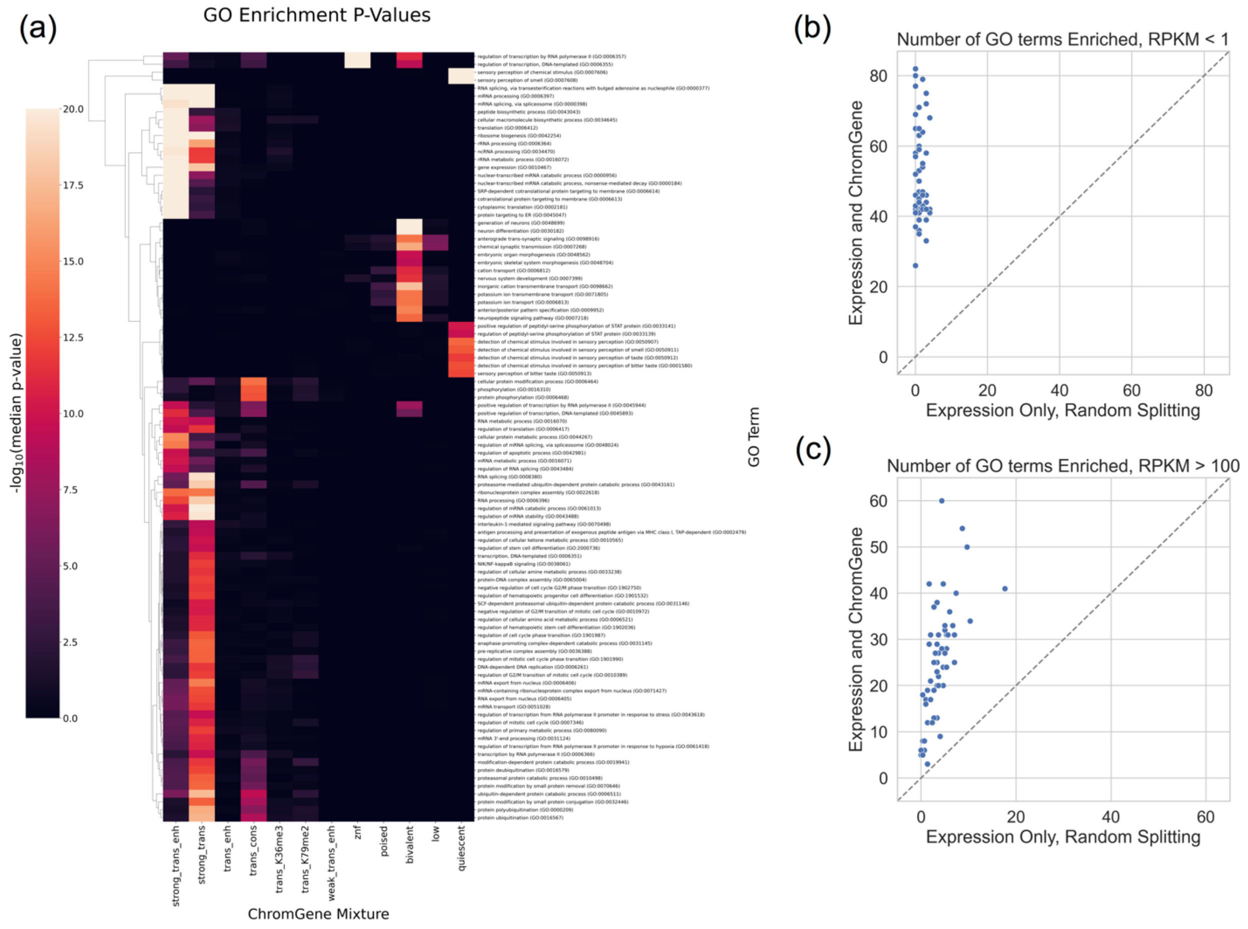

Fig. S8: Median GO term enrichment across all cell types

(a) Heatmap where the rows correspond to GO terms, columns to ChromGene annotations, and values to the median enrichment -$\log_{10}$(p-value) across all cell types. Rows are filtered so that at least one ChromGene annotation is significant (adjusted p-value < 0.01, Bonferroni corrected for the number of combinations of 12 ChromGene annotations and 6036 GO terms) and are ordered using hierarchical clustering. Maximum -$\log_{10}$(p-value) is set to 20 to prevent washing out of the color scale.

(b) The number of GO terms significantly enriched, conditioned on unexpressed genes (< 1 RPKM), when splitting randomly (horizontal axis) vs splitting by ChromGene annotations (vertical axis).

(c) The same as in (b), but for highly expressed genes (> 100 RPKM).

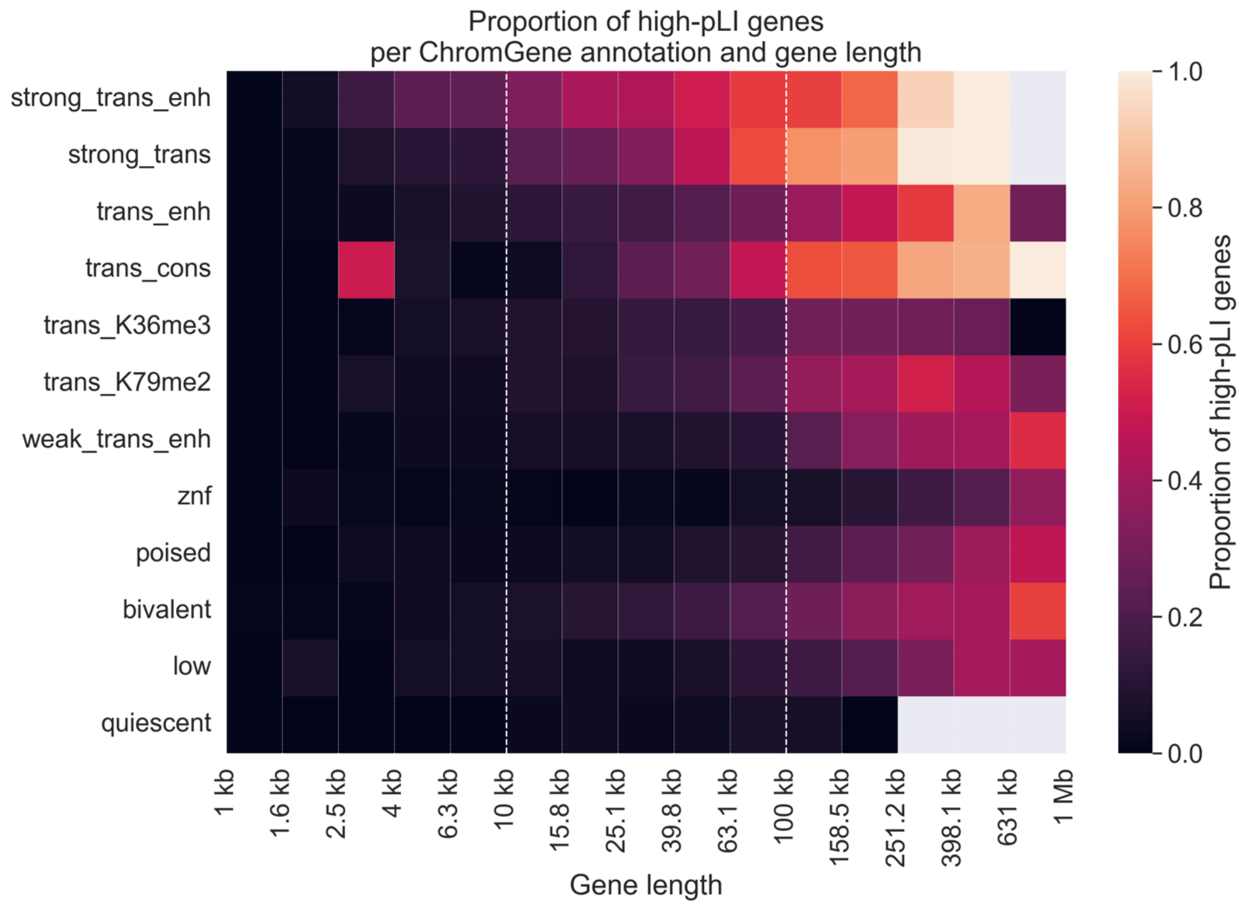

Fig. S9: Proportion of high-pLI genes per ChromGene annotation, conditioned on gene length

A heatmap showing the proportion of high-pLI (≥ 0.9) genes across all cell types for each ChromGene annotation (rows), conditioned on gene length (columns). We divided the total number of high-pLI genes for each bin by the total number of genes in that bin. Bins with 0 counts are colored grey.

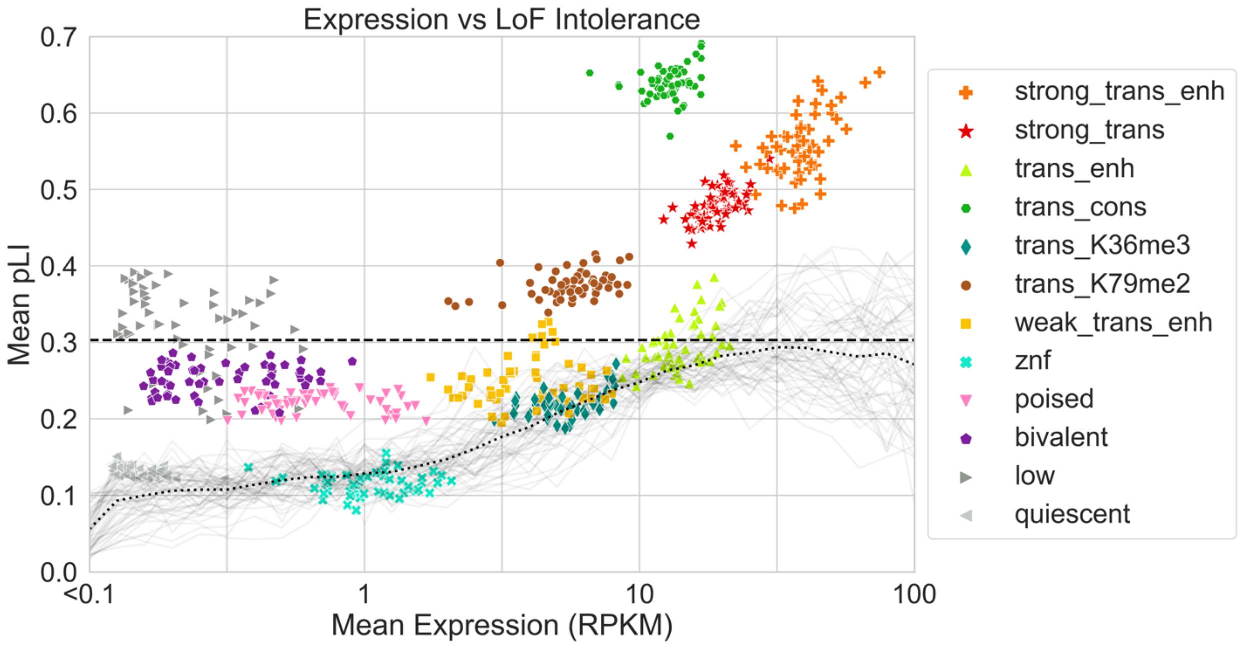

Fig. S10: Mean pLI vs mean expression

For each cell type and each ChromGene annotation combination, the mean expression (RPKM) in $\log_{10}$ scale (x-axis) and mean pLI (y-axis) are plotted. Each point represents a cell type and is colored by its ChromGene annotation. The dashed line corresponds to the mean pLI across all protein-coding genes. Gray curves represent each cell type's mean pLI score of genes as a function of expression. The dotted black line is the mean pLI averaged across cell types as a function of expression.

Metric	ChromGene	TSS	Gene Average	Collapsed
AUROC	**0.893**	0.818	0.889	0.888
MSE	**0.418**	0.685	0.429	0.438
Pearson R	**0.763**	0.601	0.757	0.753

Table S2: Performance of ChromGene compared to baseline methods at predicting expression. The rows correspond to the metric and the columns the method. The best performance for each metric is shown in bold.
