# Supplementary material for: ChromGene: gene-based modeling of epigenomic data
Source: Genome Biol. 2023 Sep 7;24:203. doi: 10.1186/s13059-023-03041-5 (PMC10486095; doi:10.1186/s13059-023-03041-5)
Supplement: Supplementary file 4 — Additional file 4. Review history. [file 13059_2023_3041_MOESM4_ESM.docx]

Review History

**First round of review**

**Reviewer 1**

**Were you able to assess all statistics in the manuscript, including the appropriateness of statistical tests used?**

No.

**Were you able to directly test the methods?**

Yes.

**Comments to author:**

The manuscript under consideration entitled "ChromGene: Gene-Base Modeling of Epigenomic Data" by Jaroszewicz and Ernst deals with a computational approach to classify genes into 12 groups based on the combinatorial and spatial patterns of multiple epigenomic marks across the gene body and flanking regions. This is achieved by learning a mixture of ChromHMM-like Hidden Markov Models across 127 cell types using the "concatenated" approach. The authors compare their (cell-type-specific) grouping in terms of gene expression, loss-of-function tolerance and additional gene annotations provided by the gene ontology and by cancer-related gene sets.

Overall, the methodological progress is rather limited as ChromGene is at its core the ChromHMM algorithm with predefined regions (protein coding transcripts/genes) and a mixture model. Although the authors provide analyses to prove the usefulness of ChromGene, I do not see how "ChromGene annotations […] will be a resource for gene-based epigenomic analyses […]" (page 4; lines 78-79) beyond the general understanding of the functional impact of the epigenome on transcription. In summary, the manuscript under consideration uses standard models to yield results that do not or only minutely so provide insights beyond the standard model of epigenomic patterns.

Specific remarks:

The annotation is based on hg19 - a genome version that has been updated to hg38 in 2013 - and a gene annotation based on Ensembl v65/GENCODE v10 (page 27; line 509). A more up-to-date data basis seems to be warranted.

The gene expression analyses reveal that genes with epigenomic patterns consistent with transcription (a state with high H3K4me3/H3K27ac = active promoter + a state with H3K36me3 or H3K79me2 = active elongation) are expressed and genes with either no histone modifications or repressed chromatin (states with high H3K9me3 or H3K27me3) show little to no transcription. Since histone modifications are correlated with gene expression, which in turn is cell-type-specific, it is not surprising to see that also the annotation of genes into one of the 12 groups is cell-type-specific. What surprised me was the strong enrichment of genes in the group "strong_trans" in cell-type-specific genes (Figure 6) given that genes in this group are enriched in "houskeeping genes" (page 18; line 309), which are defined to be not cell-type-specific.

The gene-set-enrichment analyses reveals nothing new, e.g. ZNFs are known to be decorated with a specific set of histone modifications H3K36me3 and H3K9me3 high. So it is not surprising to see an enrichment of these genes in a group of genes that is characterized by this specific combination. Furthermore, I do not see how "These results suggest that ChromGene annotations better correspond to existing biological knowledge as reflected in 'biological process' GO terms than the baseline methods." (Page 19; lines 337-338) based on the observation that more terms are annotated by ChromGene gene sets compared to the baseline methods (TSS, Gene Average, Collapsed). Such a claim would require to show that the ChromGene gene sets for a certain cell-type correspond to genes with specific functions in this cell type.

The analysis regarding the loss-of-function tolerance genes shows that "haploinsufficient" genes should be generally expressed (to the right in Figure 7) and/or expressed in many cell types, which is expected for "haploinsufficient" genes.

The repeated claim that "ChromGene captures information beyond [gene] expression." should be justified by comparing against grouping of genes based on gene expression.

**Reviewer 2**

**Were you able to assess all statistics in the manuscript, including the appropriateness of statistical tests used?**

Yes: I do not recommend additional statistical review.

**Were you able to directly test the methods?**

No.

**Comments to author:**

This manuscript presents ChromGene as an approach to assign an annotation to each gene based on its epigenetic profiles. ChromGene is built on the principles of ChromHMM, a widely used tool for genome segmentation and annotation (SAGA). However, ChromHMM and other SAGA tools assign epigenetic states to defined intervals (e.g. 200 bp bin) along chromosomes, but they do not assign entire genes to a state or annotation determined by the epigenetic profile. ChromGene learns a group of hidden Markov Models (HMMs), each with a set of states reflecting common co-occurrences of epigenetic features such as histone modifications and chromatin accessibility. It then assigns an annotation to each gene based on the likelihood of that gene's epigenetic profile being generated from the set of states from one of the group of HMMs. The genes assigned to each HMM (also called a mixture, see below), i.e. with the same ChromGene annotation, capture different genomic and biological features which provide considerable information beyond the transcription level, as detailed in several analyses in the manuscript. These annotations should be useful in many applications. The ChromGene approach should be capable of extension to other groups of genomic elements, such as non-protein-coding genes.

Several aspects of the presentation need to be improved and clarified.

(1) The ChromGene model needs to be explained more completely in the Results. Currently, one has to read the descriptions in three different sections (Results, Methods, and Supplementary Methods) to start to understand what is being done. All the essential information needed to understand the ChromGene model should be in the Results, with details in the Methods.

(2) From the initial presentation in the Results, it sounded like each gene was being assigned an annotation from some vague global matching of the epigenetic profiles (from 5' flank through the gene to the 3' flank). I kept wondering if the 200bp bins were still being assigned to epigenetic states, and indeed the Supplementary Methods finally tells the reader that each position within a gene is assigned to a state within one of the M HMMs. That step should be clarified in the initial presentation and in an additional figure panel. Based on my understanding of the information now in Supplemental Methods, I would suggest something like showing an example of state assignments throughout a gene, e.g. from two or so HMMs, and then show that one of the HMMs does a better job of generating the observed data, and that HMM is the basis for the assigned annotation. Such a diagram could also clarify how much of the 5' and 3' flanks are included, which is not stated until the Supplementary Methods. Again, such a figure panel should be part of the main presentation.

(3) Supplemental Figure S1 is much more informative than Figure 1. The innovation of using a dummy state and dummy variable to generate the M HMMs (mixtures) helps clarify what was done. Also, the Results section seems to imply that the M HMMs were generated independently, but the Supplemental Methods and Figure shows the model as a large HMM with forced divisions into smaller units (sub-HMMs? mixtures of states?).

(4) The presentation needs to clarify the distinction between "mixture" and "HMM". The legend to Figure 1 uses the term "mixture" for the entity that is referred to as "HMM" in the main text. The Results say that the data are modeled as being generated by one of M HMMs (line 88), whereas the legend says that the gene (should that be data for each gene?) is generated by one of M mixtures. Elsewhere, the manuscript states that ChromGene is a mixture of HMMs (line 84). After wondering for a while if "mixture" might refer to a group of genes, as implied by Fig. 1 ("Gene Mixture A"), I eventually settled on the mixture being a set of states (3 states per mixture for the results shown here), but the reader should not have to guess about the meaning.

(5) The manuscript should explain the evaluation that led to the choice "To balance model interpretability while capturing major biological distinctions, we chose the model with M=12 mixtures and S=3 states per mixture for all presented results." [lines 493-394 and earlier in Results]

(6) The manuscript should explain the mnemonics more thoroughly. Some inferences are likely to be correct (e.g. "trans" = transcribed), but is "cons" constitutive or conserved or perhaps something else. Does "enh" mean that a sufficient fraction of intervals within the gene unit are assigned to an enhancer-like state? Do genes in that annotation category contain intragenic enhancers?

(7) The manuscript should clarify whether position-specific information is incorporated. For instance, is the observation of higher H3K4me3 around the TSS of active genes used in the annotation (it does not appear to be), or is it the fact that some fraction of 200bp bins within the gene unit are in a H3K4me3-high state?

(8) If genes are overlapping, are the epigenetic profiles just repeated for the overlapping portions?

(9) Lines 236-240: Need to juxtapose the values for evaluating predictive capacity of ChromGene with those for the three baseline models, either as a small Table or in the text.

**Authors Response**

**Point-by-point responses to the reviewers’ comments:**

Reviewer #1:

The manuscript under consideration entitled "ChromGene: Gene-Base Modeling of Epigenomic Data" by Jaroszewicz and Ernst deals with a computational approach to classify genes into 12 groups based on the combinatorial and spatial patterns of multiple epigenomic marks across the gene body and flanking regions. This is achieved by learning a mixture of ChromHMM-like Hidden Markov Models across 127 cell types using the "concatenated" approach. The authors compare their (cell-type-specific) grouping in terms of gene expression, loss-of-function tolerance and additional gene annotations provided by the gene ontology and by cancer-related gene sets.

*Reply: We thank the reviewer for the summary and taking the time to review our manuscript. In response to the reviewer’s feedback we have made revisions that we feel strengthened the manuscript.*

*Overall, we feel in some of the reviewer’s comments below there were some misunderstandings about our manuscript. This may have been due to issues with the presentation in our initial submission that we now recognize. We have tried to substantially improve the presentation in the revised version. We apologize for any confusion because parts of our initial submission were unclear.*

*We provide our response to each of the reviewer’s comments below.*

Overall, the methodological progress is rather limited as ChromGene is at its core the ChromHMM algorithm with predefined regions (protein coding transcripts/genes) and a mixture model.

*Reply: We apologize that the methodological contributions of our work were not clear from our presentation in our initial submission as we view them to be highly significant and respectfully disagree with the characterization of them being ‘rather limited’. We have made substantial revisions to better highlight and improve our presentation of the methodological innovations in part based on the constructive feedback from Reviewer 2 in that regard.*

*We view ChromGene as offering significant methodological contributions along three major dimensions:*

*(1) One dimension is the conceptual level with our focus on gene-based epigenome modeling and annotations in contrast to per-position modeling and annotations. While many methods have been published focusing on per-position annotations from epigenomic data, often with incremental differences between them, our modeling goal is fundamentally different with our focus on genes. Since we are focusing on a fundamentally different task than previous modeling methods we do not consider this work to be an incremental advance. We approach modeling using a mixture of HMMs, which is also general, and can be applied to any predefined set of intervals of interest.*

*(2) Another innovation, as noted by reviewer 2, was our use of ‘dummy’ marks and states with restricted transitions to implement the mixture of HMMs on top of a per-position HMM modeling framework. This insight is important as it demonstrates how at least in theory the existing perposition SAGA/chromatin state modeling frameworks, for which there has already been an extensive investment by the community into their development, can be leveraged to also do gene level modeling. While ChromGene is built on top of ChromHMM, if one prefers the assumptions of a different per-position modeling framework, in principle that modeling framework could be leveraged to do gene level modeling in a similar manner.*

*(3) At a third more technical dimension, to actually get the mixture of HMMs to work in this setting, which has not been previously demonstrated, required us to understand and handle complex and subtle issues related to numerical stability of HMMs. Reflective of the technical depth of this work and the subtle issues we had to understand in order to learn the mixture of HMMs, this work led us to identify an incorrect statement in one of the primary textbook presentations of HMMs in the field of genomics (Durbin, Eddy, Krogh, and Mitchison “Probabilistic models of proteins and nucleic acids”) that has been present for more than 20 years, and for which Sean Eddy has now issued an errata (http://eddylab.org/cupbook_errata.html). We thus view actually bringing the mixture of HMM approach into practice in this setting to be a significant methodological contribution. We realize in our initial submission we omitted some of the details related to the numerical stability issues and what we did to overcome them making it difficult for the reviewer to appreciate this aspect of our contribution and we apologize for that.*

*More specifically, in this mixture setting, there will be cases where the forward-backward algorithm is applied to sequences and individual HMMs in which specific individual HMMs should have essentially zero probability of generating the observation sequence. This is especially the case in our setting as we often have relatively long sequences and low emission probabilities at a given position from considering the joint outputs of multiple marks. Due to the imprecision of floating point representation without carefully handling the numerical stability issues can lead to numerical underflow or overflow and could prevent any meaningful model output. Handling this required extensive effort from us even with one of the authors having previously written the ChromHMM code.*

*Prior to our work here, to deal with numerical stability issues ChromHMM had supported a single scaling approach that was to rescale the forward (alpha) variables of the HMMs and the backward (beta) variables at a specific position both by the sum of the forward variables at the position. This approach was consistent with that of a prominent HMM tutorial (Rabiner, 1989) which is the basis for many other presentations of HMMs including in the book Durbin, Eddy, Krogh, and Mitchison “Probabilistic models of proteins and nucleic acids”, which is a prominent reference for HMMs in genomics. However, we discovered that this scaling approach led to numerical overflow in our setting here. Namely the magnitudes of the backward variable’s at a specific position can be vastly different than the sum of the forward variables and that this can be continued to be amplified over long sequences which in some cases can lead to numerical overflow. This is likely happening specifically in this setting since the forward variables for a sequence can be based on one mixture component while the backward variables for a sequence can be dominated based on another mixture component.*

*To handle the overflow we rescaled the backward variables based on the sum of the backward variables. This is valid as Kevin Murphy previously pointed out (https://www.cs.ubc.ca/~murphyk/Papers/segment.pdf), but reflective of the subtly involved the Durbin, Eddy, Krug, and Mitchison had a statement implying having the backward variables scaled differently than the forward variables would not be valid. Based on personal communications with Sean Eddy an errata has been issued that it is not in general necessary to use the same scaling for the forward and backward variables (http://eddylab.org/cupbook_errata.html).*

*However, while rescaling the backward variables by the sum of the backward variables eliminated the numerical overflow, numerical underflow issues were then encountered. Specifically, situations were encountered in certain cases for individual genes where the product of each forward and backward variable for every state was 0 which would lead to division by 0 and no meaningful overall output. This is also likely happening in situations in which one mixture component is being used by the forward variables and another one by the backward variables. To avoid the numerical underflow if a backward variable for a state at a position fell below 10^- 300 we set its value to 10^-300. We did the same for forward variables except if the emission probability product for a state at the position was actually 0, in which case the forward variable was kept at 0. These changes were sufficient to lead to a numerically stable procedure.*

*In response to the comment and the comments on reviewer 2 on our methodological contributions we have made the following changes:*

*1. We have revised the introduction to now state: “ChromGene is distinguished from other methods in that its focus is assigning a single annotation for each gene, which can span an arbitrary length, as opposed to an annotation for each position in the genome.”*

*2. We have expanded main figure 1 and the text describing the method in the results section to highlight how per-position modeling approaches can be leveraged to generate per-gene annotations, also shown here*

*3. We have expanded our description of the method in the results section: “ChromGene models the set of epigenomic data across genes with a mixture of HMMs (Fig. 1) by extending ChromHMM [9] (Methods, Fig. 1). The set of epigenomic data for each gene along with a flanking region at each end (default 2kb) is binarized at fixed-width bins (default 200bp), indicating observations of each epigenomic mark, before being input to ChromGene. The data for a given gene in a cell type is modeled as being generated by one of M HMMs, each with S “hidden states” (or simply “states”) (black boxes, Fig. 1a). There are no constraints on allowed transitions between states of one HMM, but transitions between states of different HMMs are not allowed (Fig. 1b). There is also an initial state distribution, or the probability of seeing a state at the first position of the gene flanking region, over states of the HMMs. We note that otherwise, ChromGene does not use position-specific information in the modeling. The prior probability that a gene belongs to a specific mixture component, that is, an individual HMM, corresponds to the sum of initial probabilities of the states of that component. The emission distribution is modeled with a product of independent Bernoulli random variables following ChromHMM (Fig. 1c). For given values of M and S, the parameters of the model are learned from the data by an expectation-maximization approach aiming to maximize the likelihood of the model parameters given the data (Fig. 1d). Once a model is trained, ChromGene computes the posterior probability of each of M mixture components generating each gene’s data in a given cell type. For that cell type, each gene is then assigned to the component for which it has the greatest posterior probability (Fig. 1e). We note that while ChromGene also assigns individual bins to hidden states based on the per-state posterior probability assignments (Fig. 1e), they are not our focus here, as there are effective model-based methods for bin-level annotations.*

*To model data from multiple cell types, ChromGene can be applied analogously to the ‘concatenated’ approach of ChromHMM [2, 26], which gives cell type-specific assignments based on a common model. To do this, ChromGene treats the same gene in different cell types as if it were different genes in the same cell type when learning the model. Using this common model, a gene is independently assigned a component for each cell type.”*

*4. We have eliminated the supplementary methods and integrated all content into the main text.*

*5. We have made a separate subsection of the methods discussing numerical stability issues, which reads:*

*“Handling numerical stability issues Implementing a mixture of HMM raised some numerical stability issues not encountered with standard, single HMMs in ChromHMM. In this setting, it is expected that for some genes, there will be specific individual HMMs that would have essentially zero probability of generating the observations for the gene. Previously, to deal with numerical stability issues, ChromHMM used a single approach to scaling, which was to rescale the forward (alpha) variables of the HMMs and the backward (beta) variables at a specific position both by the sum of the forward variables. This followed previous standard presentations of HMMs [41, 42]. However, this scaling approach led to numerical overflow in our setting. Namely, the magnitudes of the backward variables at a specific position can be vastly greater than the sum of the forward variables, and this difference can be continued to be amplified over long sequences, which in some cases can lead to numerical overflow. This likely happens specifically in this setting because the forward variables for a sequence can be dominated by one mixture component while the backward variables for a sequence can be dominated by another mixture component. To handle the overflow we rescaled the backward variables based on the sum of the backward variables, which is valid as previously noted [43].*

*While rescaling the backward variables by the sum of the backward variables eliminated the numerical overflow, numerical underflow issues were then encountered. Specifically, situations were encountered in certain cases for individual genes where the product of each forward and backward variable for every state was 0, which would lead to division by 0 and no meaningful overall output. This also likely happens in certain situations in which one mixture component dominates the forward variables and another one the backward variables. To avoid the numerical underflow, if a backward variable for a state at a position fell below 10^-300 we set its value to 10^-300. We did the same for forward variables, except if the emission probability product for a state at the position was actually 0, in which case the forward variable was kept at 0. These changes were sufficient to lead to a numerically stable procedure.*

*This alternative procedure for handling numerical stability can now be accessed in ChromHMM through the use of the -scalebeta flag.”*

Although the authors provide analyses to prove the usefulness of ChromGene, I do not see how "ChromGene annotations […] will be a resource for gene-based epigenomic analyses […]" (page 4; lines 78-79) beyond the general understanding of the functional impact of the epigenome on transcription.

*Reply: We may not be understanding the reviewer’s comment here, but the reviewer appears to be acknowledging we have “provided analyses to prove the usefulness of ChromGene”, which would appear to justify calling a compendium of ChromGene annotations a ‘resource’. The use case of ChromGene annotations is specifically for when one wants to analyze genes in terms of epigenomic data which we feel is consistent with ‘gene-based epigenomic analyses’. We are not making claims about the specific applications for which it will be used. Even if it is just useful for a ‘general understanding of the functional impact of the epigenome on transcription’ we think that would already be a significant contribution, but the analyses one can use ChromGene for are agnostic to how the gene sets of interest are defined so could be used for other analyses that are not directly transcription based. We demonstrate this in the manuscript through comparing ChromGene gene annotations to various non-expression based gene annotations.*

*More generally we see potentially broad and diverse uses of ChromGene annotations given compendia of ChromHMM or similar per-position annotation methods are already used for many diverse applications and the pervasive nature of gene-based analyses in genomics. While ChromHMM and other per-position annotations can be directly used for per-position analyses they are less natural for gene-based analyses since they are not defined at a gene level. A researcher wanting to conduct gene-based analyses with ChromHMM or similar per-position annotations must define and implement a post-hoc way to relate those annotations to genes. We see analyzing genes with epigenome annotations defined at a gene level through a modelbased framework to be more natural and principled. We thus expect the compendia of annotations we created to be a useful resource for diverse applications.*

In summary, the manuscript under consideration uses standard models to yield results that do not or only minutely so provide insights beyond the standard model of epigenomic patterns.

*Reply: While ChromGene builds on top of ChromHMM the resulting model is in no way standard. We view this work as methodologically innovative for multiple reasons as explained above. ChromGene, by producing gene-based models and annotations, is completing a fundamentally different task than ChromHMM or other standard epigenomic pattern methods that provide perposition annotations. Currently, to use per-position annotations for gene-based analyses, one must resort to post-hoc procedures, which have limitations. To illustrate the purpose of ChromGene, we have expanded Fig. 1 to include a browser track, which includes raw data, perposition annotations from ChromHMM, and per-gene annotations from ChromGene.*

*We also respectfully disagree with the characterization that this provides no or only minute insights. Notably ChromGene’s model parameters directly provide a characterization of epigenomic patterns associated with different classes of genes in terms of different combinations of marks seen across the gene, which is not something directly seen in other modeling approaches. We then demonstrated how these gene-based annotations can be used for a variety of downstream analyses that position-based annotations are not designed for.*

*We believe that ChromGene annotations have advantages over any other approach for gene based epigenome analyses and thus expect it will be useful for many applications.*

Specific remarks:

The annotation is based on hg19 - a genome version that has been updated to hg38 in 2013 - and a gene annotation based on Ensembl v65/GENCODE v10 (page 27; line 509). A more up-to-date data basis seems to be warranted.

*Reply: We thank the reviewer for the comment. The reason we applied this to data on hg19 was that it was the assembly in which the Roadmap Epigenomics reference epigenomes were defined and imputed epigenomic data was available. We used Ensembl v65/GENCODE v10 to be consistent with the gene expression quantification with Roadmap Epigenomics. In response to this comment, we also applied our current model to protein coding genes from GENCODE v41 which previously had coordinates lifted from hg38 to hg19. In total, of the protein genes common between the two versions, 93.7% were assigned to exactly the same ChromGene annotation, with the remaining 6.3% being due to either different locations of gene TSSs and TESs.*

*Applying ChromGene directly to hg38 data would be a massive undertaking that would be outside the time frame of the revisions, as generating this data would require an extensive and computationally intensive set of data processing, mapping, and imputation tasks. Even after such an effort and redoing all analyses, we expect it would make little, if any difference, on end results, as our output is genes, and not genomic positions. We are currently collaborating with a large consortium which has been processing a large set of epigenomic data on hg38 and currently plan to apply ChromGene to that resource in the future.*

*We have updated the Methods sections with the following text:*

***“ChromGene annotations in hg38-based gene annotations***

*As the Roadmap Epigenomics data and gene annotations (v10) were based on the hg19 assembly, we used the same assembly and gene annotation for all analyses here [1, 16]. However, we also generated ChromGene annotations for a more recent hg38- based gene annotation, specifically the GENCODE v41 annotation [https://ftp.ebi.ac.uk/pub/databases/gencode/Gencode_human/release_41/GRCh37_ma pping/gencode.v41lift37.basic.annotation.gtf.gz], which had previously been lifted over to hg19 [16], by applying our previously-trained ChromGene model to annotate proteincoding genes. We found that when comparing common genes based on gene name, ChromGene annotations were 93.7% concordant. The 6.3% that were not concordant were due to differences in the annotated TSS or TES position; 46% of discordant genes (total of 2.9%) had a start or end position that was more than 10kb away between the two annotations.”*

*We have also added an additional supplementary file with the ChromGene annotations for GENCODE v41.*

The gene expression analyses reveal that genes with epigenomic patterns consistent with transcription (a state with high H3K4me3/H3K27ac = active promoter + a state with H3K36me3 or H3K79me2 = active elongation) are expressed and genes with either no histone modifications or repressed chromatin (states with high H3K9me3 or H3K27me3) show little to no transcription. Since histone modifications are correlated with gene expression, which in turn is cell-type-specific, it is not surprising to see that also the annotation of genes into one of the 12 groups is cell-type-specific. What surprised me was the strong enrichment of genes in the group "strong_trans" in cell-type-specific genes (Figure 6) given that genes in this group are enriched in "houskeeping genes" (page 18; line 309), which are defined to be not cell-type-specific.

*Reply: We thank the reviewer for this comment. We first note we have updated Fig. 6 in our revised version. We realized that we were previously showing log ((P(B|A)/(P(A)xP(B)) but that it would make more sense to show log(P(A,B)/(P(A)xP(B)) where A is the conditional annotation and B is the non-replicate annotation. We are now plotting log(P(A,B)/(P(A)xP(B)) and are clearer and more consistent in describing that.*

*We are unclear exactly what the reviewer is referring to in terms of ‘cell-type-specific’ genes, but we note the ‘strong_trans’ annotation had the second lowest cell type specificity scores as we quantified it (see ‘ChromGene annotations capture cell type specific patterns’) and displayed in Fig. 3, which was unaffected by the changes in Fig. 6. Based on our updated Fig. 6 the two states which showed pairwise enrichment for the ‘strong_trans’ annotation are ‘strong_trans_enh’ and ‘trans_cons’ neither of which had particularly high cell type specific scores. We apologize for any confusion caused by our previous Fig. 6.*

The gene-set-enrichment analyses reveals nothing new, e.g. ZNFs are known to be decorated with a specific set of histone modifications H3K36me3 and H3K9me3 high. So it is not surprising to see an enrichment of these genes in a group of genes that is characterized by this specific combination.

*Reply: ChromGene provides a principled model-based approach to annotate genes based on multiple epigenomic marks across the entire gene. The ChromGene model is also more expressive about the patterns associated with a gene than a single combinatorial pattern by allowing more than one state to describe the combinatorial patterns of marks across the gene. Modeling genes with more than one combinatorial pattern makes biological sense. For instance, one would expect to see a different pattern of marks at the promoter region compared to across the gene body. As an example of this, in the ZNF case the H3K9me3+H3K36me3 pattern only corresponds to one sub-state of the ZNF mixture component, while another state is associated with specific subsets of promoter/enhancers marks.*

*While it is the case that per-position chromatin states associated with H3K9me3 and H3K36me3 have been found to be enriched with ZNF genes as we cite in our manuscript, there currently lacks any existing resource which annotates what genes are associated with such a ‘ZNF’ pattern systematically across cell and tissue types. This is the case since existing epigenome annotations are based on combinatorial patterns at the position level and not the gene level. Also, as previous chromatin state enrichments of the ‘ZNF’ state for ZNF genes were only conducted at the positional level, this only provides indirect information about gene set enrichments. More generally, positional annotations can be biased by a variety of factors. For example, a small number of long genes could drive positional chromatin state enrichments. Gene set enrichments have also been conducted previously based on the chromatin state at the TSS (e.g. Ernst and Kellis, 2010), which gives each gene equal weight consistent with the baseline, but this involves significant loss of information about the epigenomic marks over the rest of the gene.*

*As the ChromGene model is new, we consider any results characterizing the individual mixture components, which we do in part by gene-set enrichment analyses, to be new here. That we see mixture components learned de novo correspond to classes of genes with mark patterns previously associated with them serves as a strong validation of the method. While there are ways to annotate genes post-hoc based on per position chromatin state (e.g. state at TSS), we consider annotating genes directly in a model based framework to conceptually be a more natural and principled analysis approach. Our comparisons with the baseline methods also show quantitatively that we can discover more biological enrichments through this modeling approach as compared to other ways to relate epigenomic annotations to genes.*

*We also did not think it was obvious a priori which ChromGene annotations would show enrichments for the cancer gene set analysis. To better highlight these results, we have made the supplementary figure on the frequency of gene set enrichments a main figure.*

*We have also revised the text surrounding the ZNF result to also contrast this work with previous work:*

*“Annotation ‘znf’ is notable in that the H3K36me3 co-occurs with the repressive mark H3K9me3. This annotation shows 11-fold enrichment for Zinc finger named (ZNF) genes on average. This is consistent with previous findings for enrichments for per-position chromatin states associated with this combination of marks for overlapping ZNF genes [8, 28], but in contrast to previous work, we are also able to provide a direct model-based annotation of all genes associated with an epigenomic pattern highly enriched in ZNF genes.”*

*In addition, we have revised and expanded our discussion to better clarify the value of ChromGene annotations relative to existing gene annotations in the following paragraph:*

*“As ChromGene directly annotates genes, it will likely be preferable for many gene-centric analyses relative to per-position chromatin state annotations. While ChromGene annotations do correspond to patterns previously identified by per-state annotations, the ChromGene annotations are more expressive in that they can associate multiple per-position patterns with the same genebased annotation. Comparing per-position annotations directly to gene annotations is subject to biases such as the differing lengths of different genes, and thus previous gene enrichment analyses using per-position annotations have often resorted to using a post-hoc approach to map per-position annotations to individual genes. ChromGene annotations avoid these issues by directly providing a model-based annotation of individual genes. We note that ChromGene annotations are likely less useful for when the object of study is not inherently gene centric, such as studying enrichments for GWAS identified variants.”*

Furthermore, I do not see how "These results suggest that ChromGene annotations better correspond to existing biological knowledge as reflected in 'biological process' GO terms than the baseline methods." (Page 19; lines 337-338) based on the observation that more terms are annotated by ChromGene gene sets compared to the baseline methods (TSS, Gene Average, Collapsed). Such a claim would require to show that the ChromGene gene sets for a certain cell-type correspond to genes with specific functions in this cell type.

*Reply: We thank the reviewer for raising this point. As there is no “ground truth” to compare to with gene set enrichments, it is difficult to quantitatively assess the GO enrichments beyond the number of terms enriched. We have updated our language to only claim we find more enriched terms. The revised sentence now reads:*

*“These results demonstrate that ChromGene annotations yield more ‘biological process’ GO term enrichments than the baseline methods”*

The analysis regarding the loss-of-function tolerance genes shows that "haploinsufficient" genes should be generally expressed (to the right in Figure 7) and/or expressed in many cell types, which is expected for "haploinsufficient" genes.

*Reply: We thank the reviewer for the comment. While there is a general trend that ChromGene annotations associated with higher expression are more likely to be “haploinsufficient”, a main point with Figure 7 is that we also see ChromGene annotations associated with similar expression associated with different pLI levels (Low vs. Quiescent and TransCons vs. TransEnh). This analysis is only considering chromatin data within a cell type and its gene expression in that cell type. It is not reasoning about the cell type specificity of a given gene or ChromGene annotation. That we observe such large differences in pLI among multiple pairs of ChromGene annotations with similar expression levels in a given cell type we consider to be a non-obvious insight. We previously had text making this point in the results section ‘ChromGene separates by pLI scores’.*

*To better emphasize this, we now illustrate in Fig. 8 (previously Fig. 7) the expected relationship between gene expression and pLI. We directly grouped genes based on their expression level, which is related to the next point, and have added lines plotting the expected pLI score as a function of gene expression. This shows how some ChromGene annotations are associated with pLI scores either well above or below the expected level based on average of genes grouped by expression level.*

*We show the updated figure and its legend below:*

*We have added the following text to the methods section to explain the process for plotting the per-celltype curves:*

*“To calculate the proportion of high pLI genes (Fig. 8) and mean of pLI scores (Supp. Fig. 8) as a function of expression for a given cell type, we took each gene’s expression, added a pseudocount of 0.1 RPKM to it, and took the log10 of the result (gray curves). We then looked at 30 equally sized bins of this transformed expression value, from -1 to 2, corresponding to 0 and ≈100 RPKM, respectively. For each bin, we took all genes falling into it, and calculated the proportion of these genes with pLI > 0.9 and the mean pLI of these genes for the two figures, respectively. We plotted the curves by linearly interpolating between bins. We then repeated the process for all cell types simultaneously (black dotted curve).”*

The repeated claim that "ChromGene captures information beyond [gene] expression." should be justified by comparing against grouping of genes based on gene expression.

*Reply: We thank the reviewer for this suggestion. We first clarify that our claim was that ChromGene provides additional information beyond gene expression, so the conditional analysis on gene expression we were presenting is sufficient for that point. There can also be cases where gene expression may capture information not contained in ChromGene annotations.*

*However, we do recognize it would be informative to directly compare to groupings based on gene expression. As mentioned above, we have now grouped genes based on gene expression level, and we report their pLI in the updated Fig. 8. In addition, we have also conducted a GO gene set enrichment analysis with low expressed genes and ChromGene annotations restricted to unexpressed or minimally-expressed genes in ‘Brain Germinal Matrix’. This analysis shows the more specific enrichments observed with additional information from ChromGene annotations. Specifically, we have added the following main figure panel and associated text in the results section:*

*“To more directly investigate whether ChromGene provides information beyond expression in the context of GO term enrichments, we compared GO term enrichments for gene sets defined based on gene expression to those defined based on their joint gene expression and ChromGene annotation. Specifically, we first computed GO term enrichment for unexpressed or minimally expressed genes (“inactive genes”, RPKM < 1.0) in ‘Brain Germinal Matrix’ using the same procedure as above, correcting for the number of GO terms (Methods), then found the most enriched term. Then, for each of the ChromGene annotations associated with the lowest expression (‘znf’, ‘poised’, ‘bivalent’, ‘low’, and ‘quiescent’), we took the subset of the inactive genes that also had the ChromGene annotation. For each subset, we repeated the GO term enrichment analysis, correcting for the number of GO terms and five ChromGene annotations (Methods), and found the most enriched GO term. We took the most enriched GO term for each of these sets, then analyzed the enrichment p-values in all the other sets considered in this analysis (Fig. 7a). We found that the GO term most enriched in inactive genes, ‘sensory perception of smell (GO:0007608)’, was substantially more enriched when subsetting to ‘quiescent’ genes despite having fewer genes in the set (2.5 fold vs 9.1 fold, p < 1E-24 vs p < 1E-52). This subset of ‘quiescent’ genes was also enriched in this GO term when using the set of all inactive genes as the background (3.1 fold, p < 1E-24). Additionally, we found that for each of the other ChromGene component subsets, its most enriched GO term had a more significant p-value than for all inactive genes. For example, the ‘bivalent’ subset was significantly enriched in ‘anterior/posterior pattern specification (GO:0009952)’ (5.4 fold, p < 1E14), but the group of all inactive genes was not (1.8 fold, p < 1E-1.8), consistent with the role of bivalent genes in development [37]. This further supports that ChromGene annotations provide additional biologically relevant information than expression alone.”*

Reviewer #2:

This manuscript presents ChromGene as an approach to assign an annotation to each gene based on its epigenetic profiles. ChromGene is built on the principles of ChromHMM, a widely used tool for genome segmentation and annotation (SAGA). However, ChromHMM and other SAGA tools assign epigenetic states to defined intervals (e.g. 200 bp bin) along chromosomes, but they do not assign entire genes to a state or annotation determined by the epigenetic profile. ChromGene learns a group of hidden Markov Models (HMMs), each with a set of states reflecting common co-occurrences of epigenetic features such as histone modifications and chromatin accessibility. It then assigns an annotation to each gene based on the likelihood of that gene's epigenetic profile being generated from the set of states from one of the group of HMMs. The genes assigned to each HMM (also called a mixture, see below), i.e. with the same ChromGene annotation, capture different genomic and biological features which provide considerable information beyond the transcription level, as detailed in several analyses in the manuscript. These annotations should be useful in many applications. The ChromGene approach should be capable of extension to other groups of genomic elements, such as non-protein-coding genes.

*Reply: We thank the reviewer for the summary and recognizing the value of the approach. We also thank the reviewer for the constructive review.*

Several aspects of the presentation need to be improved and clarified.

*Reply: We thank the reviewer for these comments which helped us to significantly improve and clarify our presentation. We describe our specific changes in response to each comment below.*

(1) The ChromGene model needs to be explained more completely in the Results. Currently, one has to read the descriptions in three different sections (Results, Methods, and Supplementary Methods) to start to understand what is being done. All the essential information needed to understand the ChromGene model should be in the Results, with details in the Methods.

*Reply: We thank the reviewer for these suggestions. We have expanded our description of the method in the results and moved any details in the methods. We no longer have a separate supplementary methods section.*

*Our section of the results introducing the model now reads as follows:*

*“ChromGene models the set of epigenomic data across genes with a mixture of HMMs (Fig. 1) by extending ChromHMM [9] (Methods, Fig. 1). The set of epigenomic data for each gene along with a flanking region at each end (default 2kb) is binarized at fixed-width bins (default 200bp), indicating observations of each epigenomic mark, before being input to ChromGene. The data for a given gene in a cell type is modeled as being generated by one of M HMMs, each with S “hidden states” (or simply “states”) (black boxes, Fig. 1a). There are no constraints on allowed transitions between states of one HMM, but transitions between states of different HMMs are not allowed (Fig. 1b). There is also an initial state distribution, or the probability of seeing a state first at the beginning of the gene flanking region, over states of the HMMs. We note that otherwise, ChromGene does not use position-specific information in the modeling. The prior probability that a gene belongs to a specific mixture component, that is, an individual HMM, corresponds to the sum of initial probabilities of the states of that component. The emission distribution is modeled with a product of independent Bernoulli random variables following ChromHMM (Fig. 1c). For given values of M and S, the parameters of the model are learned from the data by an expectation-maximization approach aiming to maximize the likelihood of the model parameters given the data (Fig. 1d). Once a model is trained, ChromGene computes the posterior probability of each of M mixture components generating each gene’s data in a given cell type. For that cell type, each gene is then assigned to the component for which it has the greatest posterior probability (Fig. 1e). We note that while ChromGene also assigns individual bins to hidden states based on the per-state posterior probability assignments (Fig. 1e), they are not our focus here, as there are effective model-based methods for bin-level annotations.*

*To model data from multiple cell types, ChromGene can be applied analogously to the ‘concatenated’ approach of ChromHMM [2, 26], which gives cell type-specific assignments based on a common model. To do this, ChromGene treats the same gene in different cell types as if it were different genes in the same cell type when learning the model. Using this common model, a gene is independently assigned a component for each cell type.*

*To implement ChromGene, we leveraged that a mixture of HMMs can be equivalently expressed using a single, specially constructed HMM. In this single HMM, there is a “dummy state” (purple) that is associated with the emission of a “dummy mark”, which is only present before or after genes and their included flanking regions (Fig. 1a, Methods). All states within mixture components are initialized to allow transitions to and from this dummy state and between states of the same mixture component, but transitions between different mixture components are disallowed by enforcing those transition probabilities to be 0. Given the equivalence to a single HMM, we trained the ChromGene model by using ChromHMM after making enhancements to ChromHMM to better handle some technical numerical stability issues in this setting (Methods). We note that this strategy of reducing a mixture of HMMs to a single per-position HMM is general, and it could in principle be used to extend other software designed for per-position annotations, which may differ from ChromHMM in modeling assumptions, to provide gene- or other region-based annotations.”*

(2) From the initial presentation in the Results, it sounded like each gene was being assigned an annotation from some vague global matching of the epigenetic profiles (from 5' flank through the gene to the 3' flank). I kept wondering if the 200bp bins were still being assigned to epigenetic states, and indeed the Supplementary Methods finally tells the reader that each position within a gene is assigned to a state within one of the M HMMs. That step should be clarified in the initial presentation and in an additional figure panel. Based on my understanding of the information now in Supplemental Methods, I would suggest something like showing an example of state assignments throughout a gene, e.g. from two or so HMMs, and then show that one of the HMMs does a better job of generating the observed data, and that HMM is the basis for the assigned annotation. Such a diagram could also clarify how much of the 5' and 3' flanks are included, which is not stated until the Supplementary Methods. Again, such a figure panel should be part of the main presentation.

*Reply: We thank the reviewer for these suggestions.*

*We have clarified our description of the methods in the results section. We clarify that each 200bp bin can be assigned to a sub-state. However, we also note our focus with ChromGene is on gene-based annotation as we see the main value of this work relative to existing per-position annotations is in the gene level annotations, and thus did not analyze the bin level annotations.*

*We have also added text to the discussion discussing this point and mention further investigation of ChromGene’s per-position annotation could be a direction for future work.*

*We have also modified figure 1 to better illustrate the method and inputs. We show this both schematically including incorporating content that was previously in Supplementary Figure S1 and with a new browser screenshot with actual data. The browser shot shows the individual input mark tracks along with the ChromGene gene and per-position assignments for a region of the genome with multiple genes having different ChromGene assignments for each gene.*

*We also now mention the amount of flanking region in the main results and methods and indicate flanking regions as part of the input in Fig. 1.*

*The updated Fig. 1 is shown here:*

(3) Supplemental Figure S1 is much more informative than Figure 1. The innovation of using a dummy state and dummy variable to generate the M HMMs (mixtures) helps clarify what was done. Also, the Results section seems to imply that the M HMMs were generated independently, but the Supplemental Methods and Figure shows the model as a large HMM with forced divisions into smaller units (sub-HMMs? mixtures of states?).

*Reply: We thank the reviewer for the comment. The two perspectives of M independent HMMs and a single HMM with divisions into smaller units are mathematically equivalent. We originally presented the former in the main as we felt it was easier to explain while the latter had implementation advantages as it could better take advantage of the existing ChromHMM software. However, reflecting on this we do feel an important methodological innovation of our work is showing how existing chromatin state/SAGA software in general, not limited to ChromHMM, can in principle be leveraged for gene or region level-annotations. We have thus moved the material from Fig S1 into Fig 1 and explicitly note the equivalence of these two perspectives in the results (see revised text provided in response to comment #1).*

(4) The presentation needs to clarify the distinction between "mixture" and "HMM". The legend to Figure 1 uses the term "mixture" for the entity that is referred to as "HMM" in the main text. The Results say that the data are modeled as being generated by one of M HMMs (line 88), whereas the legend says that the gene (should that be data for each gene?) is generated by one of M mixtures. Elsewhere, the manuscript states that ChromGene is a mixture of HMMs (line 84). After wondering for a while if "mixture" might refer to a group of genes, as implied by Fig. 1 ("Gene Mixture A"), I eventually settled on the mixture being a set of states (3 states per mixture for the results shown here), but the reader should not have to guess about the meaning.

*Reply: We thank the reviewer for raising this issue.*

*We recognize there were issues with our previous terminology. The reviewer was correct previously when we were referring to a ‘Mixture’ we were referring to a HMM with 3-states, but more proper terminology would be to call it a ‘Mixture Component’, as the overall model of all the HMMs is more properly referred to as the mixture. We have changed our use of ‘Mixture’ to ‘Mixture Component’ or ‘Component’ for brevity wherever we were referring to an individual HMM and clarified this terminology.*

(5) The manuscript should explain the evaluation that led to the choice "To balance model interpretability while capturing major biological distinctions, we chose the model with M=12 mixtures and S=3 states per mixture for all presented results." [lines 493-394 and earlier in Results]

*Reply: We thank the reviewer for this comment. We have added additional text explaining and a supplementary figure with analyses illustrating the trade-off for the number of mixture components and states per mixture component in the hyperparameter section of the methods. Our revised text and added supplementary figure are as follows:*

*“Hyperparameter selection In ChromGene, there are two hyperparameters that must be set: M, the number of mixture components, and S, the number of states per component. For M, the number of mixture components, we considered values ranging from 8 and 20 in increments of two. For S, the number of states per component, we considered each value ranging from 2 to 5, while S=1 corresponded to the Gene Average baseline.*

*The choice of M trades-off the ability of the model to capture additional classes of genes while maintaining meaningful distinctions between each annotation. To assess this tradeoff, we calculated ChromGene’s reproducibility, defined as the percentage of identical assignments in cell types that can be considered replicates (below, Calculation of confusion matrix and contingency table), as a function of M and S. We found that reproducibility is more sensitive to the choice of M than S (Supp. Fig. 1a). We found that there was a large drop in reproducibility between M=12 mixture components and 14 or more. On the other hand, the drop in reproducibility from M=8 and M=10 to M=12 was relatively minimal, suggesting 12 mixture components could provide a reasonable tradeoff for the expressivity of the model without having any mixture components that are largely redundant (Supp. Fig. 1b).*

*The choice of S trades off the expressivity of the state representation within a mixture component with the overall interpretability of each mixture component. A mixture component with too many states could have “redundant states”, where two or more states have very similar emission parameters. To investigate this tradeoff, we calculated the mean Manhattan distance between the emission parameters of the two closest states within a mixture, for values of M=8 to 20, and S=2 to 5 (Supp. Fig. 1c). We note that S=1 is excluded from this analysis because two states are needed to calculate a distance. Unlike with reproducibility, this metric was more sensitive to the choice of S than M. We found that the mean Manhattan distance in models using S=4 or S=5 states per mixture have a similarly low value, suggesting that these models have pairs of “redundant” states that are not well-differentiated by emission parameters (Supp. Fig. 1d). However, there is still an appreciable gap between S=3 and both S=4 and S=5, supporting the choice of S=3 to trade off model expressivity while maintaining a relatively interpretable model with limited redundancy between sub-states of the model.*

*Supplementary Figure 1: Model reproducibility and parameter similarity as a function of hyperparameters (a) Reproducibility of ChromGene annotations as a function of M and S (Methods). Reproducibility is more sensitive to the choice of M than S. (b) Reproducibility as a function of M, represented as a boxplot over values of S. (c) Manhattan distance between two closest states within a mixture, for values of M=8 to 20, and S=2 to 5. (d) Manhattan distance between two closest states within a mixture as a function of S, represented as a boxplot over values of M.*

(6) The manuscript should explain the mnemonics more thoroughly. Some inferences are likely to be correct (e.g. "trans" = transcribed), but is "cons" constitutive or conserved or perhaps something else. Does "enh" mean that a sufficient fraction of intervals within the gene unit are assigned to an enhancer-like state? Do genes in that annotation category contain intragenic enhancers?

*Reply: We thank the reviewer for the suggestion. We have clarified our mnemonics more thoroughly. The ‘cons’ was for constrained. The ‘enh’ mnemonic was used for mixture components which had a substate associated with the enhancer mark H3K4me1 but not associated with H3K4me3. As we are only considering a limited region of flanking for genes we can assume that these are largely candidate intragenic enhancers. We have revised the text to now include the abbreviations to mnemonics:*

*(‘trans’: ‘transcribed’, ‘enh’: ‘enhancer’, ‘cons’: ‘constrained’)*

*We have also edited our sentence describing the enhancer associated ChromGene annotations to now read:*

*Three of the annotations (‘strong_trans_enh’, ‘trans_enh’, and ‘weak_trans_enh’) had a state with H3K4me1 being the mark with the greatest frequency (>78% in all cases) and limited H3K4me3 (<5% of positions), consistent with previously described putative enhancers [8, 27, 28], suggesting these ChromGene annotations contain intragenic enhancers.*

(7) The manuscript should clarify whether position-specific information is incorporated. For instance, is the observation of higher H3K4me3 around the TSS of active genes used in the annotation (it does not appear to be), or is it the fact that some fraction of 200bp bins within the gene unit are in a H3K4me3-high state?

*Reply: We thank the reviewer for the question. Positional information is used to define the start and end of regions considered along with a +/- 2kb flanking region. There can be a weak contribution through the initial probabilities to favor finding the H3K4me3 near the TSS, but in general we expect it will be more driven that some fraction of the 200bp bins are in the H3K4me3 high state.*

*To clarify we have added an additional sentence in the main text after our sentence describing the initial state distribution:*

*“There is also an initial state distribution, or the probability of seeing a state at the first position of the gene flanking region, over states of the HMMs. We note that otherwise, ChromGene does not use position-specific information in the modeling.”*

(8) If genes are overlapping, are the epigenetic profiles just repeated for the overlapping portions?

*Reply: We thank the reviewer for the question. Yes, they are. To clarify this point, we have added a sentence to the Methods section under Training data:*

*“Overlapping genes were considered separately with their overlapping regions repeated.”*

(9) Lines 236-240: Need to juxtapose the values for evaluating predictive capacity of ChromGene with those for the three baseline models, either as a small Table or in the text.

*Reply: We thank the reviewer for the suggestion. We have now juxtaposed the values in the comparison in the text as follows:*

*We implemented each baseline method to have an identical number of clusters as our ChromGene model. ChromGene annotations were significantly more predictive of gene expression than the three baseline methods (Supp. Table 2, AUROC = 0.893 vs 0.818, 0.889, and 0.888; MSE = 0.418 vs 0.685, 0.429, and 0.438; Pearson r = 0.763 vs 0.601, 0.757, and 0.753; p < 10-4 for all comparisons, paired binomial test, for ChromGene vs TSS, Gene Average, and Collapsed Models, respectively, Methods).*

*We have also juxtaposed the values in an added Supplementary Table (shown below)*

**Second round of review**

**Reviewer 1**

This is the first revised version of the manuscript “ChromGene: Gene-Base Modeling

of Epigenomic Data” by Jaroszewicz and Ernst.

In the response to my objections the authors try to explain the innovations of ChromGene.

1. I agree that the idea to provide a “gene” annotation is useful

The remaining explanations are straightforward. However, these ideas have not been implemented in this manner.

But this can easily be done by defining the appropriate transition matrix as indicated in Figure 1b along side with the corresponding emission matrix.

2. I do not understand the utility and innovative character of the dummy state: Starting from the observation vector (consecutive 200 bp bins along a gene, including 2 kb up- and downstream of the gene) for each gene in each cell type (concatenate) the HMM can be forced to transition from a “begin” state at the start of the observation vector to any state of any of the 12 “mixtures” and forced to transition back to an “end” state at the end of the observation vector. This procedure is pretty standard.

3. the numerical instability has obviously been solved by Eddy et al.

I did not understand Figure 6 and I still don’t. What is the purpose of this Figure?

This is the text, which refers to Figure 6:

Page 17 top: "We next assessed how ChromGene assignments for the same gene vary across pairs of cell types. We first conducted enrichment analyses of ChromGene annotations across pairs of cell types relative to the number of genes assigned to each annotation (Fig. 6), excluding pairs of cell types that could be considered essentially biological replicates."

Which does not explain why I should look at Figure 6 at all. The section is about that "ChromGene annotations capture cell type-specific patterns".

The whole “Brain Germinal Matrix” analysis leaves me completely baffled

Page 20 line 375+: "Specifically, we first computed GO term enrichment for unexpressed or minimally expressed genes (“inactive genes”, RPKM < 1.0) in ‘Brain Germinal Matrix’ [...] We found that the GO term most enriched in inactive genes, ‘sensory perception of smell (GO:0007608)’, was substantially more enriched when subsetting to ‘quiescent’ genes […] For example, the ‘bivalent’ subset was significantly enriched in ‘anterior/posterior pattern specification (GO:0009952)’ (5.4 fold, adjusted p < 1E-14), but the group of all inactive genes was not (1.8 fold, adjusted p < 1E-1.8), consistent with the role of bivalent genes in development [37]."

So what do we learn from this? In “Brain Germinal Matrix” unexpressed genes carry functions in the sensory perception of smell. And the enrichment of these genes becomes more acute when subsetting on the "quiescent" genes. So "Brain Germinal Matrix" unexpressed AND quiescent genes are olfactory receptors, which is probably true for most cell types/tissues.

The authors as well as me also expect that “bivalent” genes in any cell type should be enriched in “anterior/posterior pattern specification”.

It would be nice to see whether additional biologically RELEVANT information can be gained that link the chromatin state of genes of cells to their functions rather than to what genes that these cells do not express do.

The whole GO Term analysis section now demonstrates that “These results demonstrate that ChromGene annotations yield more enriched ‘biological process’ GO terms than the baseline methods.” So in my opinion this section is now unnecessary as it does not show why ChromGene is useful,

I suggest that the authors should do a differential analysis between two cell types and changing ChromGene annotations, e.g. genes that maintain the ChromGene annotation and genes that change it and compare that to changes in gene expression. If then something interesting surfaces then I would be convinced that something has been gained.

In reading the manuscript again, I still feel that the correlation of the "annotations" (= mixture components) by ChromGene with other "annotations" (GO annotation, cancer gene set annotations etc.) are due to confounding with other attributes of the underlying genes, e.g.

Page 24, top: "Notably, 'trans_cons' had a substantially larger percentage of high-pLI genes (46.8%) than 'trans_enh' (13.0%), despite a positive correlation between pLI score and expression (spearman r from 0.18 to 0.39, mean r=0.27 across cell types, all p-values < 10-100, Methods), and 'trans_cons' having slightly lower overall expression than 'trans_enh' (median RPKM = 12.73 and 13.89, respectively). This difference is consistent with the substantial difference in gene length between the 'trans_cons' and 'trans_enh' annotations (Fig. 5), and a previously noted positive correlation."

In other words: that trans_cons' had a substantially larger percentage of high-pLI genes (46.8%) than 'trans_enh' (13.0%) can be explained by the substantial difference in gene length between the 'trans_cons' and 'trans_enh' annotations. Thus, the association between trans_cons and high-pLI genes could be indirect via the gene length.

Specific remarks:

Page 19, line 351+:

In contrast, a set of previously defined housekeeping genes, based on broad and constant expression levels [36], were most enriched (3.0 fold, p < 10-300) in the 'strong_trans' annotation, which was simultaneously 99% depleted for constitutively unexpressed genes.

What is the meaning of 99% depleted for constitutively unexpressed genes?

Page 21, line 389+:

For example, the 'bivalent' subset was significantly enriched in 'anterior/posterior pattern specification (GO:0009952)' (5.4 fold, adjusted p < 1E-14), but the group of all inactive genes was not (1.8 fold, adjusted p < 1E-1.8), consistent with the role of bivalent genes in development [37].

"p < 1E-1.8" is probably a typo.

**Reviewer 2**

The manuscript has been revised extensively to clarify all the points raised in the initial review. This revised manuscript is a clear presentation of an important new method that provides a gene-based annotation from epigenetic profiles. The advantages of the ChromGene approach to multiple types of analyses are demonstrated and discussed well.

**Authors Response**

**Point-by-point responses to the reviewers’ comments:**

Reviewer #1:

This is the first revised version of the manuscript “ChromGene: Gene-Base Modeling

of Epigenomic Data” by Jaroszewicz and Ernst.

*Reply: We thank the reviewer for taking the time to review our manuscript again and for providing additional comments that we feel have improved the manuscript.*

In the response to my objections the authors try to explain the innovations of ChromGene.

1. I agree that the idea to provide a “gene” annotation is useful

*Reply: We thank the reviewer for recognizing the utility of providing “gene” annotations.*

The remaining explanations are straightforward. However, these ideas have not been implemented in this manner.

But this can easily be done by defining the appropriate transition matrix as indicated in Figure 1b along side with the corresponding emission matrix.

*Reply: As we explain below, we do not consider actually bringing this approach into practice easy at a technical level. However, regardless of one’s assessment of the technical challenge involved, we feel doing something conceptually novel and useful should already be sufficient to merit publication.*

2. I do not understand the utility and innovative character of the dummy state: Starting from the observation vector (consecutive 200 bp bins along a gene, including 2 kb up- and downstream of the gene) for each gene in each cell type (concatenate) the HMM can be forced to transition from a “begin” state at the start of the observation vector to any state of any of the 12 “mixtures” and forced to transition back to an “end” state at the end of the observation vector. This procedure is pretty standard.

*Reply: We apologize that the utility of the dummy state was not clear. The utility of the dummy state relative to having separate start and end states without a direct transition from the end state to the start state, is that it allows us to place multiple protein coding genes in the same file. This is important since we are handling approximately 20,000 separate protein coding genes for 127 cell types, and if we used an implementation that required placing each gene and each cell type in a separate file this would require creating 2.5 million files. Such a large number of files can put us over operating system limits for the number of files. A transition from the end state to the start state should produce equivalent results, but a single dummy state has an advantage of a reduction in model complexity.*

*We view the main innovation here as the overall conceptualization of how existing chromatin state frameworks can be used to produce gene-level annotations using a mixture of HMMs with minimal changes, which we are not aware of being previously done. In addition to adding dummy mark/states, this also included adding transition restrictions. While our solution might appear intuitive with the benefit of hindsight, it was not obvious to us when we started this project and Reviewer 2 also previously noted the innovation in this aspect.*

*To make clearer how the dummy state allows us to include many genes in the same file, we have added the following to the text under the Methods section:*

*“We note that in theory, we could apply an equivalent model without dummy marks and states by creating a separate file for each combination of gene and cell type. However, such a process for our training scenario would produce approximately 2.5 million files, which can be over system limits for the number of files.”*

3. the numerical instability has obviously been solved by Eddy et al.

*We apologize if our previous response was unclear on this point, since our point before was that the scaling approach to handle numerical instability is not actually solved by following the approach presented in the Durbin, Eddy, Krogh, Mitchison book. Prior to our work here, ChromHMM previously only implemented the scaling approach to handle numerical instability that was consistent with the presentation of HMMs in Durbin, Eddy, Krogh, Mitchison as well many other presentations. In all our previous applications of ChromHMM, we found that procedure was sufficient to be numerically stable. However, we found that the procedure as presented was insufficient for our application here with ChromGene. Moreover, the book had a statement that implied the solution that we developed (described in ‘Handling Numerical Stability Issues’ in our Methods section and our previous response) would not actually be valid. Specifically, the book in Chapter 3 had stated:*

*“The b variables have to be scaled with the same variables”. In response to our communications, Sean Eddy has now issued an errata stating: “change ‘have to be scaled’ to ‘can be scaled’”. He has also credited us for pointing this out (see* [*http://eddylab.org/cupbook_errata.html*](http://eddylab.org/cupbook_errata.html)*).*

*We note that the Durbin, Eddy, Krogh, Mitchison book does also present an alternative logbased approach to handle numerical stability, but in our previous experience that approach is far too slow for the types of applications we are considering, and this approach is not implemented in ChromHMM.*

I did not understand Figure 6 and I still don’t. What is the purpose of this Figure?

This is the text, which refers to Figure 6:

Page 17 top: "We next assessed how ChromGene assignments for the same gene vary across pairs of cell types. We first conducted enrichment analyses of ChromGene annotations across pairs of cell types relative to the number of genes assigned to each annotation (Fig. 6), excluding pairs of cell types that could be considered essentially biological replicates."

Which does not explain why I should look at Figure 6 at all. The section is about that "ChromGene annotations capture cell type-specific patterns".

*Reply: We thank the reviewer for the feedback and we apologize that the purpose of Figure 6 was not clear. The purpose of Figure 6 is to show which pairs of states a gene is most likely to transition between across cell types. For example, the figure shows the ‘trans_cons’ annotation is very unlikely to be in the ‘quiescent’ annotation in another cell type where the expectation is computed from the prior probability of genes being assigned to each annotation. The prior probabilities are based on the empirical overall frequency of assignments to each annotation. This is consistent with the expectation given that the ‘trans_cons’ annotation is associated with housekeeping genes. Overall, this analysis allows us to further characterize the different ChromGene annotations and in particular their relationship across cell types.*

*To clarify, we have revised the section header to state “Characterizing ChromGene assignments across cell types”*

*We have also added the following text to clarify our observations:*

*“We found that certain pairs of ChromGene annotations are strongly depleted for the same gene across pairs of cell types. For example, genes assigned in one cell type to ‘trans_cons’, which is highly enriched with housekeeping genes, are strongly depleted for the ‘quiescent’ annotation in other cell types (176 fold depletion compared to expected, corresponding to a -7.46 log2 ratio), consistent with the ‘quiescent’ annotation’s enrichment for olfactory genes. On the other hand, we also found certain pairs of annotations enriched for the same gene across cell types. For example, genes assigned to ‘poised’ in some cell type were enriched for both ‘weak_trans_enh’ and ‘low’ annotations in others, reflecting this annotation’s association with genes that can be activated or repressed in other cell types.”*

The whole “Brain Germinal Matrix” analysis leaves me completely baffled

*Reply: We thank the reviewer for the feedback and we apologize for any confusion. As discussed below, we have tried to clarify the analysis.*

Page 20 line 375+: "Specifically, we first computed GO term enrichment for unexpressed or minimally expressed genes (“inactive genes”, RPKM < 1.0) in ‘Brain Germinal Matrix’ [...] We found that the GO term most enriched in inactive genes, ‘sensory perception of smell (GO:0007608)’, was substantially more enriched when subsetting to ‘quiescent’ genes […] For example, the ‘bivalent’ subset was significantly enriched in ‘anterior/posterior pattern specification (GO:0009952)’ (5.4 fold, adjusted p < 1E-14), but the group of all inactive genes was not (1.8 fold, adjusted p < 1E-1.8), consistent with the role of bivalent genes in development [37]."

So what do we learn from this? In “Brain Germinal Matrix” unexpressed genes carry functions in the sensory perception of smell. And the enrichment of these genes becomes more acute when subsetting on the "quiescent" genes. So "Brain Germinal Matrix" unexpressed AND quiescent genes are olfactory receptors, which is probably true for most cell types/tissues.

*We apologize that the point of the analysis was unclear. The point we were trying to make here is that by incorporating ChromGene annotation information, we can identify a smaller set of unexpressed genes that correspond to specific biological classes of genes than when using gene expression alone.*

*To clarify this, we have updated the text to read:*

*“Together, these results suggest that using ChromGene in tandem with expression information can be used to identify subsets of genes with more specific biological roles than by expression alone. Further, they show that ChromGene can provide useful biological information even in the absence of significant expression.”*

The authors as well as me also expect that “bivalent” genes in any cell type should be enriched in “anterior/posterior pattern specification”.

It would be nice to see whether additional biologically RELEVANT information can be gained that link the chromatin state of genes of cells to their functions rather than to what genes that these cells do not express do.

*Reply: We thank the reviewer for the suggestion. We have now added an additional analogous analysis based on highly-expressed genes that links the genes to their cell types.*

*However, as noted above, we do feel the analysis based on unexpressed genes is informative as it demonstrates how ChromGene annotations can identify subsets of unexpressed genes with specific important biological functions, thus highlighting the additional information they have beyond gene expression.*

*In response to this comment, we have added a new figure, and revised the text to read:*

*“We repeated the analysis with highly expressed genes (RPKM > 100) using the ChromGene annotations ‘strong_trans_enh’, ‘strong_trans’, and ‘trans_enh’ and again found increased significance of notable GO terms when using ChromGene annotations to subset these expressed genes. For example, in the ‘Fetal Brain Female’ cell type, we found the ChromGene annotation ‘trans_enh’ was significantly enriched for ‘neural tube development (GO:0021915)’ (61.5 fold, adjusted p < 0.01), but was not significantly enriched using expression only (Fig. 7b). We note that when using a background of highly expressed genes instead of all genes, we observed a marginally significant enrichment (4.7 fold enrichment, p < 0.002).”*

*7: GO term and cancer gene set enrichment (a) Heatmap showing enrichment -log10(p-values) of top GO terms (rows) for six gene groups (columns): all “inactive genes” (< 1.0 RPKM), and subsets of those genes assigned to each of five ChromGene annotations associated with lower expression. Asterisks denote significant enrichments after multiple testing correction (Methods). Square brackets denote the number of genes in each GO term and ChromGene annotation. The first row corresponds to the most enriched GO term based on expression only. The second through sixth rows correspond to the most enriched GO term for each of the subsets corresponding to the ChromGene annotations listed in the second through sixth columns, respectively. ‘Sensory perception of smell’ is listed twice since it was most enriched based on both expression only and the subset with the ‘quiescent’ ChromGene annotation. (b) As in (a), but for highly expressed genes (> 100 RPKM), and using the three ChromGene annotations with the highest overall expression, ‘strong_trans_enh’, ‘strong_trans’, and ‘trans_enh’. (c) Count of how often each ChromGene annotations yielded significant p-values (adjusted p < 0.01, Bonferroni corrected for the number of combinations of 127 cell types, 12 components, and gene sets) across 6036 biological process GO term gene sets. (d) As in (c), but for 967 cancer gene sets.*

The whole GO Term analysis section now demonstrates that “These results demonstrate that ChromGene annotations yield more enriched ‘biological process’ GO terms than the baseline methods.” So in my opinion this section is now unnecessary as it does not show why ChromGene is useful,

*Reply: We thank the reviewer for raising this point. To clarify our position: while we cannot definitively claim ChromGene is better based on the number of GO terms, we do in general expect methods that show a greater number of GO terms enriched to more likely capture relevant biology. This is an assumption that has made in other previous papers in the field. We acknowledge there are limitations to this type of analysis, but do we feel it still has some value.*

*In response to this comment, we have updated the manuscript to read:*

*“To quantitatively confirm that ChromGene annotations provide additional information for finding enrichments, we compared the number of GO terms enriched when splitting genes by ChromGene assignments to splitting genes randomly. We found that for both the unexpressed (< 1 RPKM) and highly expressed (> 100 RPKM) genes, for all 56 cell types tested, ChromGene yielded more enriched GO terms (p < 10-16, paired binomial test) (Supp. Fig. 8b,c, Methods). As previous studies have assumed that increased GO significance corresponds to greater biological significance [40, 41], these results would suggest increased biological relevance of using ChromGene annotations. However, we note that it is possible a method’s annotations could yield more significant GO enrichments while not necessarily being more biologically relevant, which would be difficult to determine without inherent ground truth. Together, these results suggest that using ChromGene in tandem with expression information can be used to identify subsets of genes with more specific biological roles than by expression alone.”*

I suggest that the authors should do a differential analysis between two cell types and changing ChromGene annotations, e.g. genes that maintain the ChromGene annotation and genes that change it and compare that to changes in gene expression. If then something interesting surfaces then I would be convinced that something has been gained.

*Reply: We thank the reviewer for the suggestion. We have performed an additional analysis showing how changes in ChromGene annotation across cell types corresponds to changes in gene expression. We have added supplementary figure 7, added our analysis methodology under the “Methods” section, and added the following text under the section “Characterizing ChromGene assignments across cell types”:*

*“We next evaluated the extent to which changes in a gene’s ChromGene assignment across pairs of cell types were reflected (on average) in a change in expression of that gene (Supp. Fig. 7a). We found that for most pairs of ChromGene annotations, changes in expression were largely consistent with their overall individual expression patterns (Fig. 4, Supp. Fig. 7c,e). For example, if a gene was assigned in cell type i to an annotation associated with low expression, such as ‘quiescent’, and a high-expression annotation in cell type j, such as ‘strong_trans_enh’, then the expression of that gene in cell type j would typically be substantially higher than in i (Methods). Interestingly, we found a few pairs of ChromGene annotations for which expression did not vary as much as expected based on individual patterns (Supp. Fig. 7c,e). Together, these results show that while changes in ChromGene assignment across cell types are typically reflected in expression changes for their associated annotations, some genes do not follow this pattern.”*

*Supplementary Figure 7: Comparison of gene expression as a function of ChromGene annotations across pairs of cell types (a) A heatmap showing log ratio in expression [log2(RPKMj / RPKMi)] for genes in ChromGene annotation i in the conditional cell type (rows) and ChromGene annotation j in the “other” cell type (columns), averaged over combinations of genes and cell type pairs. Positive values correspond to a higher expression of the gene in the latter cell type and ChromGene annotation. Certain pairs of ChromGene annotations had less variation in expression than expected based on individual expression distributions (‘znf’-‘strong_trans’, ‘quiescent’-‘strong_trans’, ‘quiescent’- ‘trans_K36me3’, ‘quiescent’-‘trans_K79me2’). For example, if a gene was assigned to ‘znf’ in cell type i and ‘strong_trans’ in cell type j, then the expression only increased on average by 1.2 fold (log2 fold change of 0.23), as compared to an expected change of 19.4 fold (log2 fold change of 4.27) as determined by dividing the median expressions of those annotations across all cell types. These results are unlikely to be artifacts of low counts, as we found 11094, 570, 25410, and 16803 such changes in ChromGene assignment across all pairs of cell types, respectively. In this scenario, the mean expression of genes in the ‘strong_trans’ annotation for cell type j (7.08 RPKM) was lower than the overall mean expression of ‘strong_trans’ (18.24 RPKM), while genes in the ‘znf’ annotation were higher (6.03 RPKM) than overall (0.94 RPKM) (Supp. Fig. 7d). (b) The expected log ratio in expression [log2(RPKMj / RPKMi)], represented as in (Supp. Fig. 7a). Values were calculated based on median expression for each ChromGene annotation (Fig 3, Supp. Table 1, Methods). (c) The observed minus expected log ratio in expression, taken by subtracting the matrix represented in (Supp. Fig. 7b) from that in (Supp. Fig. 7a). (d) A heatmap showing the mean expression [log10(RPKM)] for genes in the ChromGene annotation j in the “other” cell type (columns) conditioned on the same gene being assigned to ChromGene annotation i in another cell type (rows). (e) A scatterplot showing the observed minus expected log ratio in expression between pairs of ChromGene annotations. Each point is a pair of ChromGene annotations.*

In reading the manuscript again, I still feel that the correlation of the "annotations" (= mixture components) by ChromGene with other "annotations" (GO annotation, cancer gene set annotations etc.) are due to confounding with other attributes of the underlying genes, e.g.

Page 24, top: "Notably, 'trans_cons' had a substantially larger percentage of high-pLI genes (46.8%) than 'trans_enh' (13.0%), despite a positive correlation between pLI score and expression (spearman r from 0.18 to 0.39, mean r=0.27 across cell types, all p-values < 10-100, Methods), and 'trans_cons' having slightly lower overall expression than 'trans_enh' (median RPKM = 12.73 and 13.89, respectively). This difference is consistent with the substantial difference in gene length between the 'trans_cons' and 'trans_enh' annotations (Fig. 5), and a previously noted positive correlation."

In other words: that trans_cons' had a substantially larger percentage of high-pLI genes (46.8%) than 'trans_enh' (13.0%) can be explained by the substantial difference in gene length between the 'trans_cons' and 'trans_enh' annotations. Thus, the association between trans_cons and high-pLI genes could be indirect via the gene length.

*Reply: We thank the reviewer for raising this point. We have performed an additional analysis to show that the difference between ‘trans_cons’ and ‘trans_enh’ percentages of high-pLI genes goes beyond what can be explained by gene length. We have added the following text to the manuscript:*

*“We still saw a difference in percentage of high-pLI genes between ‘trans_cons’ and ‘trans_enh’ after correcting for gene length, although it did decrease, with 28.8% and 20.9%, respectively, of annotated genes having high pLI scores (Methods, Supp. Fig. 9, Supp. Table 1). … Among the lowly expressed ChromGene annotations, ‘low’ and ‘quiescent’ had the greatest difference in proportion of high-pLI genes (21.7% and 1.0%, respectively), although this difference was smaller in a gene length-matched distribution (8.9% vs 2.9%, respectively) (Methods)”*

*We have also added the following paragraph to the methods section under the heading “pLI score analysis”:*

*“To show that the difference in percentage of high-pLI genes among ChromGene annotations (Fig. 8) is not simply due to differences in gene length, for each cell type, we first took all genes assigned to a ChromGene annotation, then split them into bins based on the log10 of their length, where the log10 length ranged from 3 (1000bp) to 6 (1Mbp), and each bin spanned 0.2 log10 length, for a total of 15 bins. We used the range of 1kb-1Mb as there are very few genes outside this range (394 genes with length < 1kb, 63 genes with length > 1MB). For each length bin and ChromGene annotation, we counted the number of genes in the bin with high pLI score (≥ 0.9) across all cell types. We then normalized the resulting values to yield the proportion of genes with high pLI score for each ChromGene annotation and length bin (Supp. Fig. 9). To explicitly compare the differences in pLI scores across annotations controlling for gene length, we first binned all gene lengths by their log10 length as above, normalized so the distribution summed to 1, and used this as the reference gene length density. Then, we took the proportions of genes with high pLI scores per ChromGene annotation across the bins, as described above, and multiplied by the reference gene length density. Finally, for each ChromGene annotation, we summed the resulting values to obtain a gene length-normalized proportion of high-pLI score genes (Supp. Table 1).”*

Specific remarks:

Page 19, line 351+:

In contrast, a set of previously defined housekeeping genes, based on broad and constant expression levels [36], were most enriched (3.0 fold, p < 10-300) in the 'strong_trans' annotation, which was simultaneously 99% depleted for constitutively unexpressed genes.

What is the meaning of 99% depleted for constitutively unexpressed genes?

*Reply: We thank the reviewer for pointing out this was unclear. We have clarified this sentence to say: “...which had a 131 fold depletion for constitutively expressed genes”.*

Page 21, line 389+:

For example, the 'bivalent' subset was significantly enriched in 'anterior/posterior pattern specification (GO:0009952)' (5.4 fold, adjusted p < 1E-14), but the group of all inactive genes was not (1.8 fold, adjusted p < 1E-1.8), consistent with the role of bivalent genes in development [37].

"p < 1E-1.8" is probably a typo.

*Reply: We thank the reviewer for pointing this out. The value is correct but the text around it should have been worded differently. We are also providing a decimal value instead of a value in scientific notation. We have revised the text to now read:*

*“For example, the ‘bivalent’ subset was strongly enriched in ‘anterior/posterior pattern specification (GO:0009952)’ (5.4 fold, adjusted p < 1E-14), but the group of all inactive genes was only marginally enriched (1.8 fold, adjusted p = 0.016).”*

Reviewer #2:

The manuscript has been revised extensively to clarify all the points raised in the initial review. This revised manuscript is a clear presentation of an important new method that provides a gene-based annotation from epigenetic profiles. The advantages of the ChromGene approach to multiple types of analyses are demonstrated and discussed well.

*Reply: We thank the reviewer for the positive comment and providing constructive feedback in the previous round that led us to improve the manuscript.*

**Third round of review**

**Reviewer 1**

**The authors have addressed all remaining points in my second review.**

**I apologize for the late response to the review request.**
